# Three reversibly interconvertible redox states of boradigermaallyl: syntheses of radical allyl anion and allyl dianion

**DOI:** 10.1039/d6sc00727a

**Published:** 2026-03-02

**Authors:** Stefan F. Miehe, Klaus Eichele, Hartmut Schubert, Holger F. Bettinger, Christian P. Sindlinger, Lars Wesemann

**Affiliations:** a Institut für Anorganische Chemie, Eberhard Karls Universität Tübingen Auf der Morgenstelle 18 72076 Tübingen Germany lars.wesemann@uni-tuebingen.de; b Institut für Organische Chemie, Eberhard Karls Universität Tübingen Auf der Morgenstelle 18 72076 Tübingen Germany holger.bettinger@uni-tuebingen.de; c Institut für Anorganische Chemie, Universität Stuttgart Pfaffenwaldring 55 70569 Stuttgart Germany christian.sindlinger@iac.uni-stuttgart.de

## Abstract

A methyl derivate of our previously published chloro-boradigermaallyl, which features a borylene unit stabilized by a chelating bis-germylene ligand, is synthesized by addition of MeBBr_2_ to the bis(germylene) A followed by KC_8_ reduction. Both derivatives, the BCl (1a) and BMe (1b) boradigermaallyls, feature an allyl-type delocalized Ge–B–Ge 2π-electron system. In this work, the reversible two step reduction of both compounds to the persistent radical anions and dianions is presented. EPR data and particularly hyperfine coupling constants to the ^11^B and ^73^Ge nuclei confirm allyl-type delocalization of the radicals. Computed spin densities illustrate the structural analogy to the organic allyl radical. Cyclic voltammetry measurements of the boradigermaallyl compounds exhibit one reduction wave within the accessible electrochemical window indicating a reduction potential of the dianionic species beyond −2.2 V [*vs.* (Ag/Ag^+^)].

## Abstract 2

Radical anion and dianion were characterized as products of a reversible stepwise two-electron reduction of boradigermaallyl.

## Graphical abstract

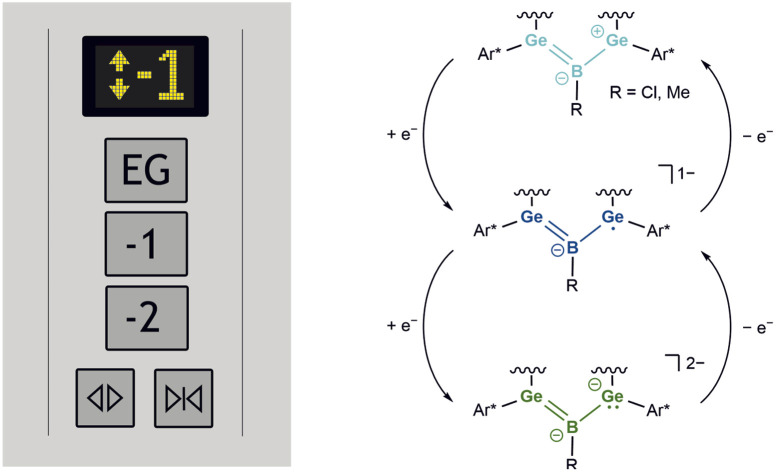

## Introduction

The allyl cation, radical and anion are small resonance stabilized organic compounds that represent examples of fundamental structural building blocks in organic matter.^1–5^ With three aligned p-orbitals occupied with two, three or four π-electrons, these molecules belong to the group of elementary conjugated systems. As intermediates in organic chemistry, the allyl cation and allyl radical play an important role in many synthetic transformations.^6–9^ The allyl anion on the other hand, which is used as allyl lithium or allyl magnesium halide for example, is a versatile starting material for organic and organometallic syntheses.^10–14^ In heavier group-14 element chemistry the search for heavy analogues of unsaturated organic molecules is a field of major interest (for example alkenes,^15–21^ allenes,^22^ alkynes,^23–25^ cyclopropenes,^26^ benzene,^27,28^ vinylidene^29,30^).^26,31–33^ A variety of group-15 element analogues of the allyl anion has been published.^34–38^ Heavy analogues of allyl cations have been presented for bismuth, silicon and germanium.^39–42^ Schleyer and coworkers studied the structures of the trisilaallyl anion [Si_3_H_5_]^−^ using quantum chemical computations and discussed hyperconjugation as a substantial stabilization for the nonplanar structures.^43^ Sekiguchi and coworkers synthesized an allyl-type radical and anion of silicon reducing the cyclotetrasilenylium cation [(*t*-Bu_2_MeSiSi)_3_Si*t*-Bu_2_]^+^ with alkali metals Li, Na or potassium graphite (KC_8_), *vide infra*.^44–47^

**Scheme 1:**
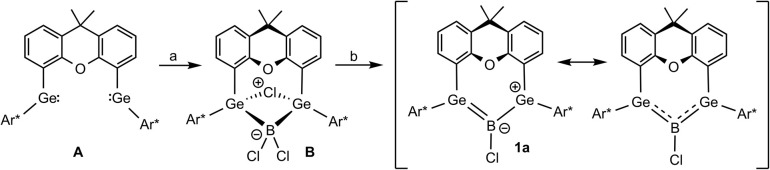
Synthesis of boradigermaallyl [(a) Me_2_S·BCl_3_, (b) {(MesNacnac)Mg}_2_] [Ar* = C_6_H_3_-2,6-(Trip)_2_, Trip = 2,4,6-C_6_H_2_-*i*Pr_3_, MesNacnac = {[(Mes)NC(Me)]_2_CH}^−^, Mes = 2,4,6-C_6_H_2_Me_3_].^48,49^

With the insertion of boron trichloride into a bis(germylene) A (Scheme 1), followed by a two-electron reduction of the addition product B, we have recently synthesized an allyl cation analogue 1a featuring delocalization of two electrons in the Ge–B–Ge π-system generated by linear combination of the three vacant p-orbitals.^48^

**Scheme 2:**
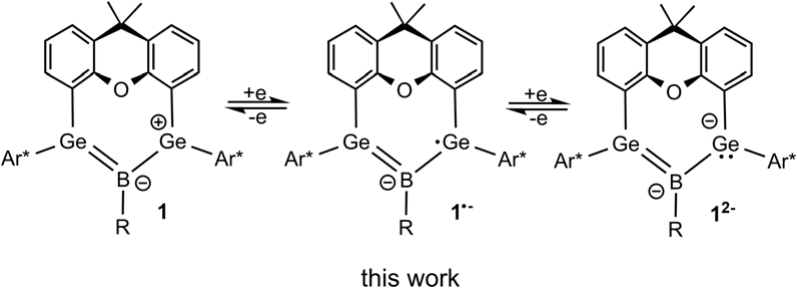
Three isolable redox states of boradigermaallyl (1a R = Cl, 1b R = Me).

With the aim of reductively breaking the remaining B–Cl bond in 1a, we discovered that 1a can be reduced (Scheme 2). This led us to investigate the redox properties of boradigermaallyl.

**Scheme 3:**
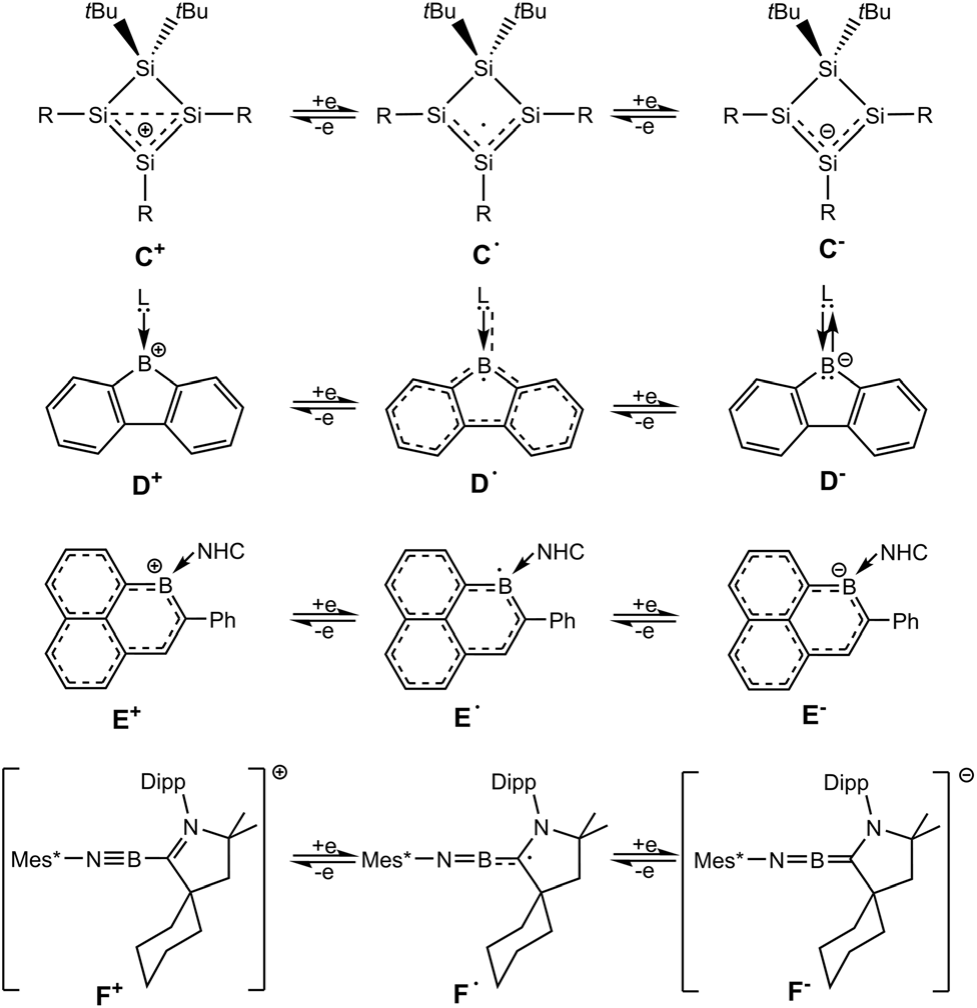
Selected known compounds featuring interconvertible redox states. [R = SiMe*t*-Bu_2_, L = CAAC: (2,6-diisopropylphenyl)-4,4-diethyl-2,2-dimethyl-pyrrolidin-5-ylidene; 1,3-bis(2,6-diisopropylphenyl)imidazol-2-ylidene, NHC = 1,3-diisopropyl-4,5-dimethylimidazol-2-ylidene, Mes* = 2,4,6-tri*t*-butylphenyl].

Molecular compounds that can be transferred reversibly into three isolable redox states are recently of interest in main group element chemistry.^37,50–64^ However, because of redox instabilities, compounds accessible in three redox states are rare. The cyclotetrasilenylium cation (C^+^, Scheme 3) was reduced by Sekiguchi and co-workers treating the cation with Li, Na or KC_8_ to give the red-purple coloured radical.^46^ The subsequent reduction of the cyclotetrasilenyl radical was carried out using lithium as reducing agent. The reversibility of the reduction has been demonstrated treating the anion with [Et_3_Si(C_6_H_6_)][B(C_6_F_5_)_4_] to give the radical as the product of a one-electron oxidation and finally the radical is oxidized after addition of [Ph_3_C][B(C_6_F_5_)_4_].^45,46^

Reduction of a CAAC- or NHC-adduct of borafluorene cations yields the radical species (D˙, Scheme 3).^65,66^ Monoanionic borafluorene adducts were obtained by reducing the 9-bromo-9-borafluorene adducts with strong reducing reagents (Li-naphthalenide, Na, KC_8_).^66–70^ The interconversion of a NHC-adduct of boraphenalene (E^+^, Scheme 3) into three different redox states has been presented with the reversible one- and two-electron reduction using KC_8_ and oxidation in reaction with AgSbF_6_.^71^ The CAAC-adduct of a phenyl substituted iminoboryl compound (F^+^, Scheme 3) shows a one-electron reduction in reaction with cobaltocene Cp*_2_Co to give the radical and the anion is formed after addition of Li/naphthalene.^72^ A T-shaped organoboron dication with a PNP-pincer ligand was reversibly reduced to the radical cation and neutral base-stabilized borylene.^63^ Furthermore, both oxidation products of a BN analogue of Thiele's hydrocarbon, a radical cation and after the second oxidation the dication, have been isolated and characterized.^73^ Most recently the reversible electron transfer in the formation of an anionic radical and dianionic 1,4-diborabutatriene has been reported.^74^

In this manuscript we present the synthesis of methyl-substituted boradigermaallyl, the reversible stepwise two-electron reduction of boradigermaallyl derivatives 1 and discuss the structural and spectroscopic findings of the reduction products (Scheme 2).

## Results and discussion

**Scheme 4:**
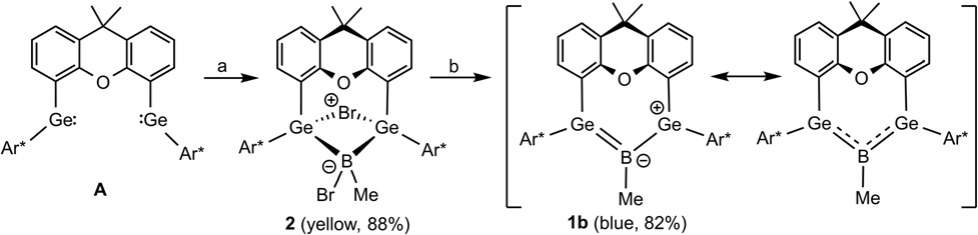
Synthesis of the methyl derivative 1b of boradigermaallyl ((a) *n*-pentane, 1 equiv. MeBBr_2_·SMe_2_, 2 h, rt; (b) Et_2_O, 2 equiv. KC_8_, rt, 30 min).

In the first step of this project, we synthesized a methyl substituted derivative of boradigermaallyl because we wanted to compare the properties of the chloro- and methyl-substituted allyl system 1. The oxidative addition of MeBBr_2_·SMe_2_ to the bis(germylene) A is a straightforward reaction (Scheme 4) and the product 2 was isolated as yellow crystals in 88% yield. Reduction was carried out treating the bromide 2 with two equivalents of KC_8_ in Et_2_O at room temperature. The solution turned green and finally blue to yield blue crystals of the methyl boradigermaallyl 1b in a yield of 82% after crystallization at −40 °C.

**Fig. 1:**
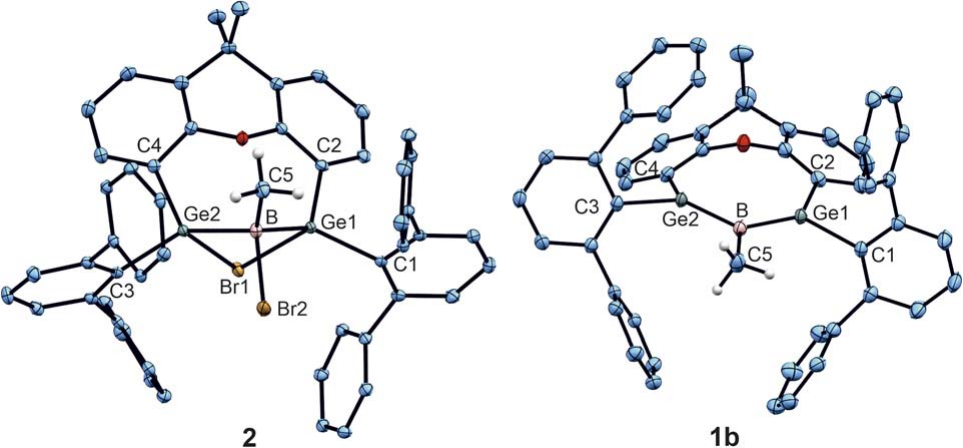
ORTEPs of the molecular structures of 1b (right) and 2 (left). Thermal ellipsoids are shown at 50% probability. *i*-Pr groups and hydrogen atoms except the hydrogen atoms of the B–Me unit have been omitted. Selected interatomic distances [Å] and angles [°]: 1b B–Ge1 1.962(4), B–Ge2 1.948(5), B–C1 1.597(5), Ge2–B–Ge1 121.4(2), C1–B–Ge1 118.4(2), C1–B–Ge2 120.1(2), 133.4 folding angle of xanthene backbone (folding angle of 1a 138.6); 2: B–Ge1 2.1196(15), B–Ge2 2.1269(15), B–C1 1.567(10), B–Br 2.0547(16), Ge1–Br1 2.5324(2), Ge2–Br1 2.5725(2), 143.8° folding angle of xanthene backbone (folding angle of xanthene backbone B 142.5).

The molecular structure of the MeBBr_2_ addition product 2, presented in Fig. 1, can be compared with the BCl_3_ addition product B (Scheme 1) and shows Ge–B distances in the same range [B: Ge–B 2.121(2), 2.117(2) Å]. Methyl-boradigermaallyl 1b, the molecular structure of which is shown in Fig. 1, exhibits short Ge–B distances of 1.948(5) and 1.962(4) Å which are almost as long as those in the chloro derivative 1a [Ge–B 1.960(2), 1.962(2) Å] and can be compared to Ge–B double bonds of germaborenes [1.886(2)–1.967(4) Å].^20,75–77^ The Ge–B–Ge angle of 121.4(2)° is slightly more acute than in 1a [Ge–B–Ge 126.7(1)°]. The Ge–B–C angles in 1b [118.4(1), 120.1(1)°] are slightly larger than the Ge–B–Cl angles in 1a [116.2(1), 117.1(1)°], which is in line with Bent's rule.^78,79^ The signal in the ^11^B NMR spectrum of 1b was observed at 41.9 ppm (1a 42.4 ppm). The solution UV-Vis spectrum of 1b features an absorption band at 636 nm. Based on TD-DFT calculations we qualitatively assign this absorption to the HOMO → LUMO transition (see SI).

**Scheme 5:**
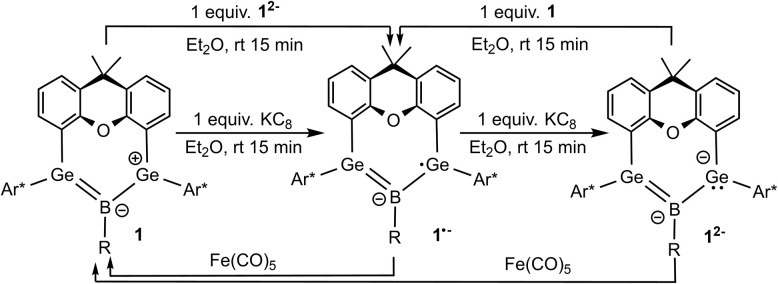
Reduction of 1a, 1b and radical anions 1a˙^−^ and 1b˙^−^. oxidation of dianions 1a^2−^, 1b^2−^ and radical anions 1a˙^−^ and 1b˙^−^ ((a) R = Cl, (b) R = Me).

**Scheme 6:**
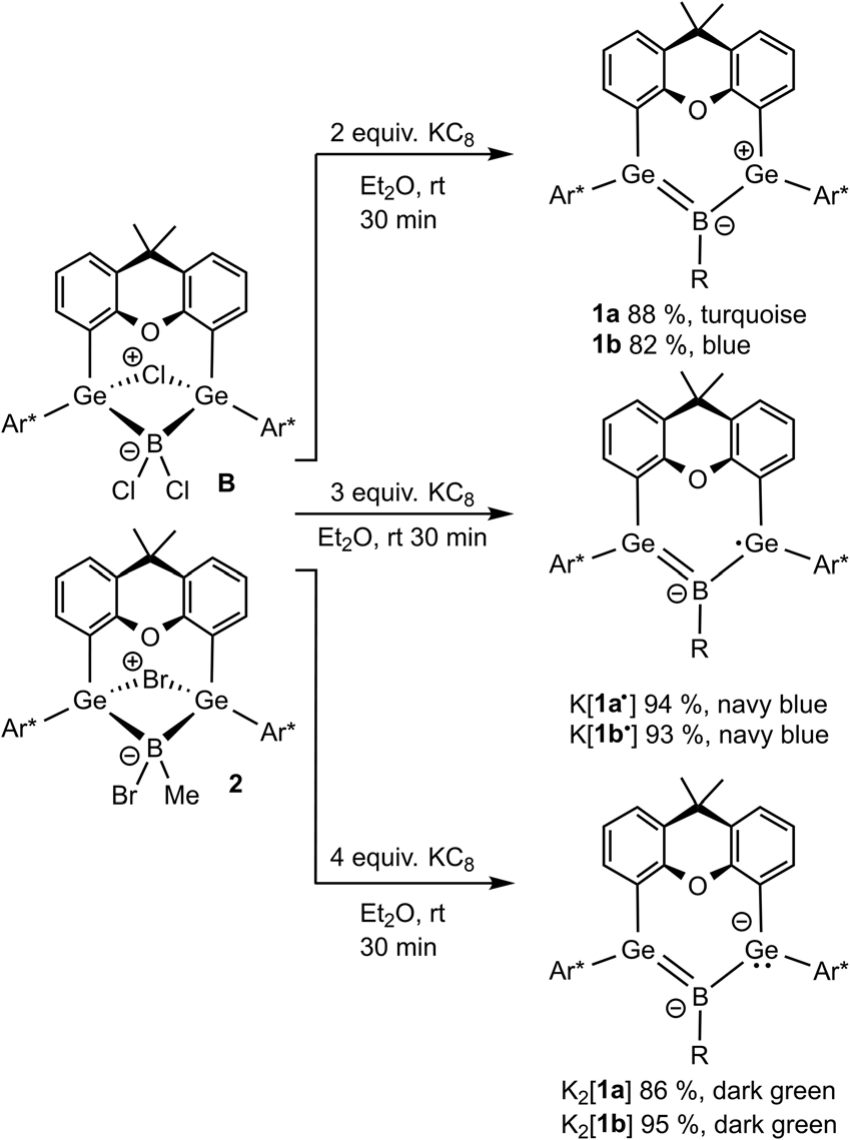
Syntheses of boradigermaallyl (1a, 1b), radical anion salts (K[1a˙], K[1b˙]) and dianion salts (K_2_[1a], K_2_[1b]) ((a) R = Cl, (b) R = Me).

The reduction of boradigermaallyl derivatives 1a and 1b (Fig. 3 and Scheme 5) was initially carried out by reaction with one equivalent of KC_8_ in diethyl ether at room temperature to yield the persistent radical anions 1˙^−^ as potassium salts. However, the reduction of the halides 2 and B has been established as a straightforward synthesis of radical anions 1a˙^−^ and 1b˙^−^ (Scheme 6). Since three equivalents of KC_8_ are used in this procedure, the weighing error is comparatively smaller and there is a smaller chance of over-reduction. During our investigations, no decomposition of 1˙^−^ was observed at room temperature in solution over a period of several weeks.

**Fig. 2:**
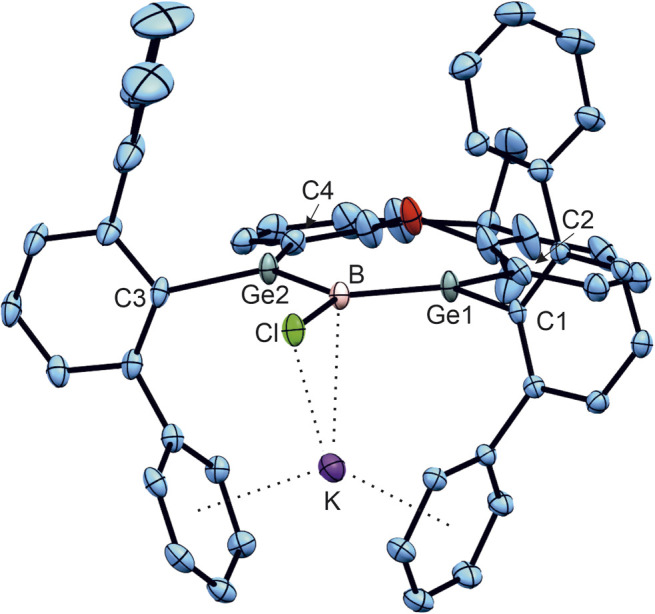
ORTEP of the molecular structures of K[1a˙]. Thermal ellipsoids are shown at 50% probability. *i*-Pr groups and hydrogen atoms have been omitted. Selected interatomic distances [Å] and angles [°]: B–Cl 1.851(3), Ge1–B 1.960(3), Ge2–B 1.967(3), K–Cl 3.0474(9), K–B 3.058(3), K–C (arene) 3.23(2) – 3.0474(9), Ge1–B–Ge2 131.8(1), Ge1–B–Cl 114.4(1), Ge2–B–Cl 113.7(1), 159.1° folding angle of xanthene backbone.

The reversibility of the reduction was demonstrated by oxidation of 1˙^−^ to the starting material 1 through the addition of iron pentacarbonyl (Scheme 5). Crystallization at −40 °C in diethyl ether gives navy blue crystals of K[1a˙] and K[1b˙]. The molecular structure of the potassium salt of the reduction product K[1a˙] is depicted in Fig. 2.

**Fig. 3:**
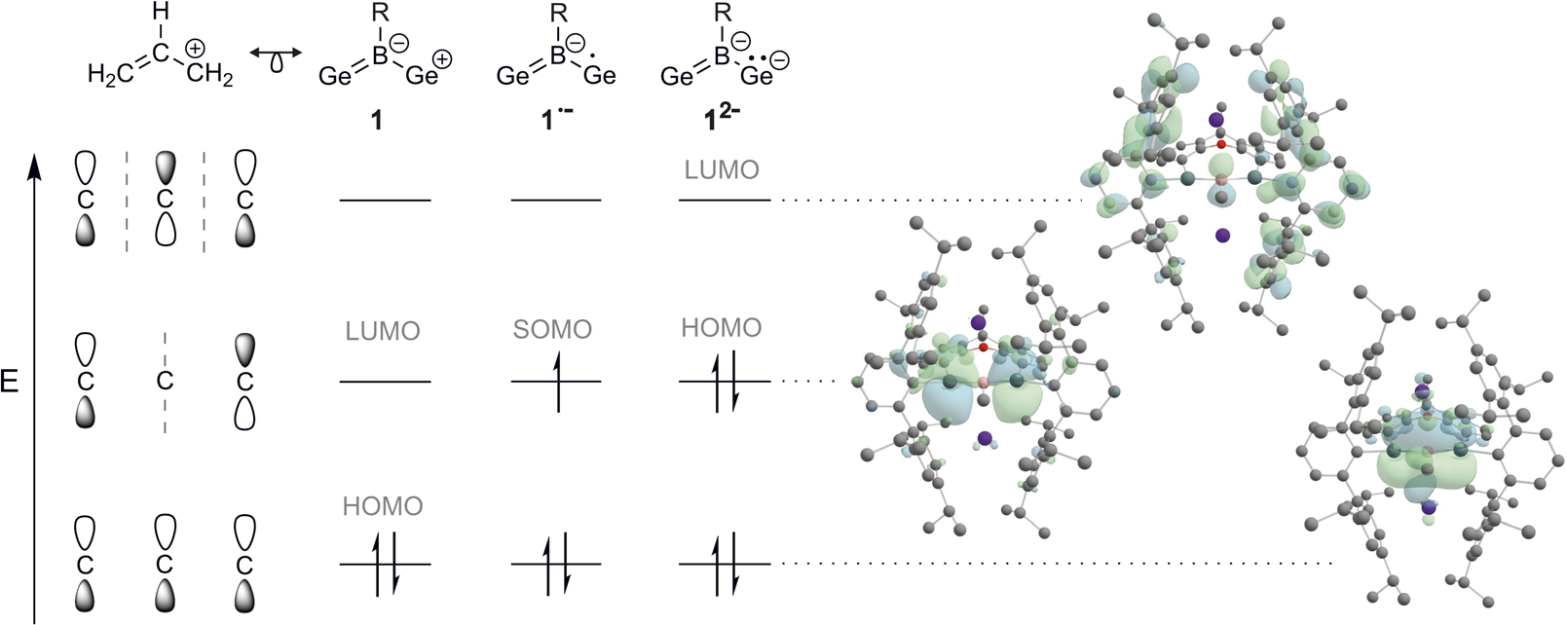
Isolobal analogy between the organic allyl cation and the Ge–B–Ge heteroallyl system: linear combinations of three aligned p-orbitals forming bonding, nonbonding and antibonding orbital combinations, exemplified by the organic allyl system (left). Electron occupancy of frontier orbitals of 1, K[1˙] and K_2_[1] (middle) and corresponding molecular orbitals of K_2_[1a] at the r^2^SCAN-3c level of theory (right). For energies of frontier orbitals see SI Table SI12.

The radical anion 1a˙^−^ exhibits almost no elongation of the Ge–B bonds lengths in comparison to the starting material 1a. The Ge–B–Ge angle of 131.8(1)° is significantly more obtuse than in 1a [Ge–B–Ge 126.7(1)°]. However, isolated crystals of K[1b˙] were of low quality, and only the positions of the heavy atoms, which have a structural motif analogous to K[1a˙], could be determined. The solution UV-Vis spectra of K[1a˙] and K[1b˙] feature a broadened absorption around 606 nm (K[1a˙]) and 650 nm (K[1b˙]). Based on TD-DFT calculations we qualitatively assign these absorptions to the SOMO → LUMO + *n* (*n* = 0,1,2) transitions (see SI).

**Fig. 4:**
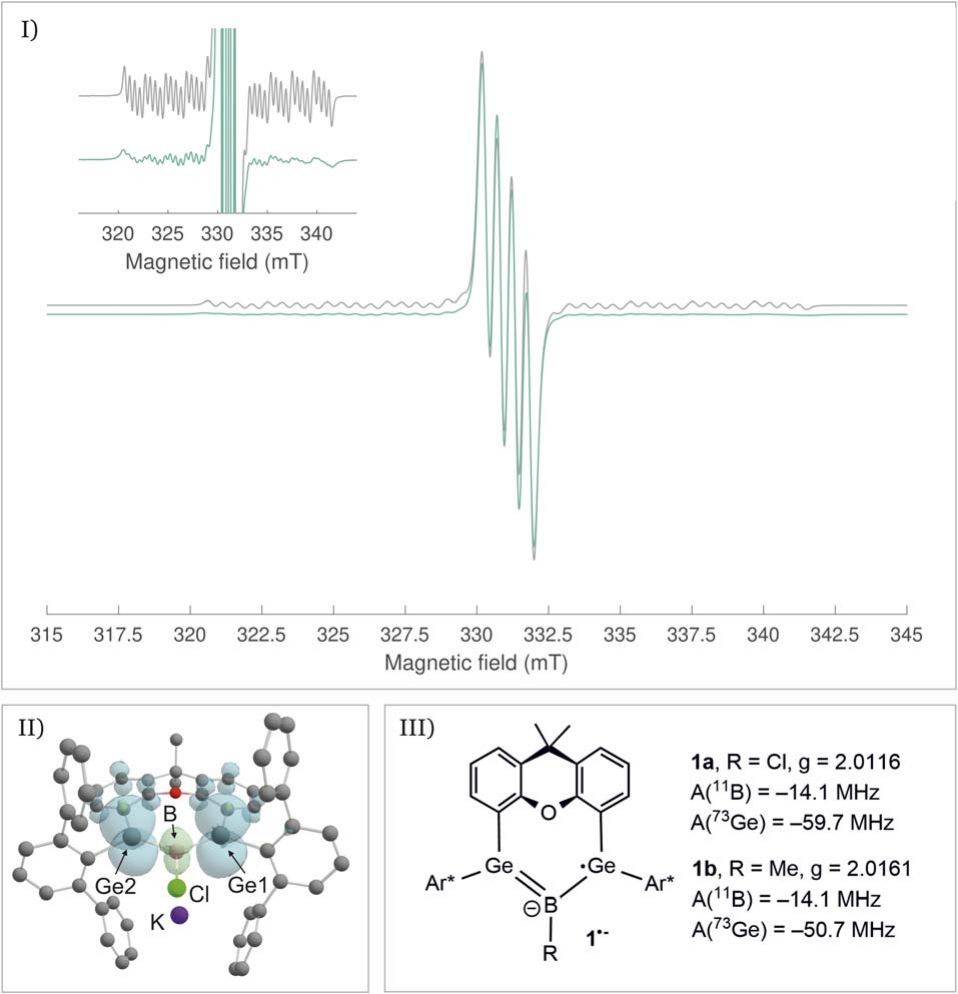
(I) cw X-band EPR spectrum (rt) of K[1a˙] (turquoise = experimental, grey = simulation), (II) spin density distribution of K[1a˙] (ωB97X-V/CPCM(Et_2_O)), (III) experimentally determined EPR parameters of K[1a˙] and K[1b˙].

**Scheme 7:**
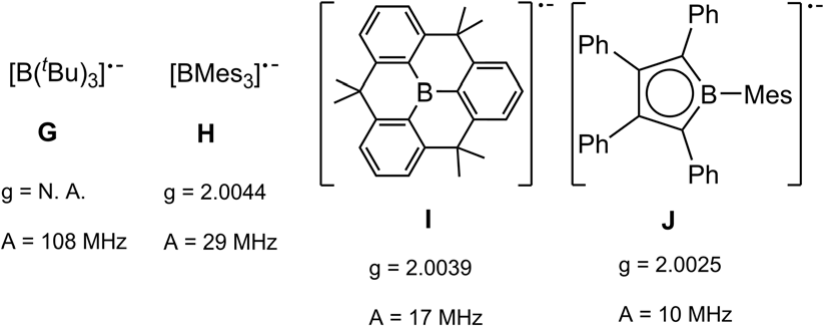
Examples for boron-containing radical species G,^89^H,^90–92^I,^93^J.^94^

**Scheme 8:**
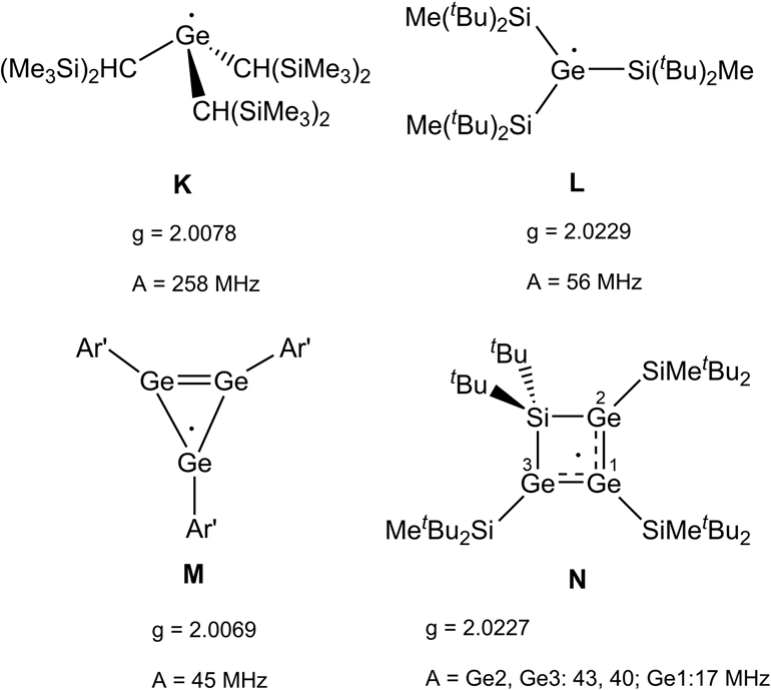
Examples for germanium-containing radical species K,^84,85^L,^87^M,^42^N.^47^

The radical character of K[1a˙] and K[1b˙] became evident by the fact that the compounds are NMR silent. The continuous-wave EPR spectra were measured in diethyl ether at room temperature and subsequently simulated (see SI for details). The spectra (Fig. 4, see SI for EPR of 1b˙^−^) of 1a˙^−^ and 1b˙^−^ yield isotropic *g*-factors of *g*_iso_ = 2.0116 (1a˙^−^) and *g*_iso_ = 2.0161 (1b˙^−^).^80,81^ Due to hyperfine coupling with the ^11^B (I = 3/2, 80.1% abundance) and ^73^Ge (I = 9/2, 7.8% abundance) isotopes 1˙^−^ exhibit spectra with a central four-line resonance [*A*_iso_(^11^B) = −14.1 MHz (1a˙^−^, 1b˙^−^)] featuring ^73^Ge hyperfine coupling [*A*_iso_(^73^Ge) = −59.65 MHz (1a˙^−^) and −50.69 MHz (1b˙^−^)]. The EPR parameters of 1a˙^−^ and 1b˙^−^ were computed using different density functional methods, dispersion corrections and solvation models (see SI). The computed *g*_iso_ values of 1a˙^−^ and 1b˙^−^ are in excellent agreement with the experimental data across all methods [1a˙^−^ (*g*_iso_ = 2.0131–2.0116), 1b˙^−^ (*g*_iso_ = 2.0124–2.015)]. Regarding the hyperfine coupling constants, the ωB97X-V//CAM-B3LYP method provides values for 1a˙^−^ which are very close to the experimental reference, however, the experimental values of 1b˙^−^ could not be reproduced as accurately using the same method [results of computations: *A*_iso_(^73^Ge) = −58.35 MHz (1a˙^−^) and −41.87 (1b˙^−^), *A*_iso_(^11^B) = −14.12 MHz (1a˙^−^) and −20.64 (1b˙^−^)]. Spin density distribution of 1a˙^−^ (Fig. 4) was computed using different functionals (see SI). A dominant localization of the spin density on the two germanium atoms with a small negative spin density at the boron centre and partial delocalization onto the xanthene backbone was found. Mulliken atomic spin densities of the boradigermaallyl radical anion 1a˙^−^ [ωB97X-V/CPCM(Et_2_O), Ge1: 0.565, Ge2: 0.563, B: −0.224] indicate a structural resemblance to the allyl radical (C^1^H_2_ = C^2^H–C^3^H_2_˙; C^1^, C^3^: 0.575 (ref. 82)/0.583,^83^ C^2^: −0.179 (ref. 82)/−0.170 (ref. 83)) showing comparable spin densities and illustrate the textbook character of the boradigermaallyl system. The hyperfine coupling constant (hfcc) *A*_iso_(^11^B) for the boron atom in 1a˙^−^ and 1b˙^−^ of −14.1 MHz is smaller in magnitude than those observed in G and H (Scheme 7). This indicates a less localized spin density on boron atoms in 1a˙^−^ and 1b˙^−^ and can be compared with delocalized spin densities found in I (*A* = 17 MHz) and J (*A* = 10 MHz) featuring comparable hfccs for the boron atom. In the case of the hyperfine coupling constants *A*_iso_(^73^Ge) for the germanium atoms [*A*_iso_(^73^Ge) = −59.65 MHz (1a˙^−^), −50.69 MHz (1b˙^−^)] the observed small hfccs also indicate a delocalized π-radical. The values are much smaller than the hfcc found in the pyramidal radical K featuring s-orbital character in the SOMO (Scheme 8).^84–86^ The hfcc of 1a˙^−^ and 1b˙^−^ can be compared with the values documented for radicals L, M and N (Scheme 8). Compound L is a planar π-radical with the 3p_*z*_ orbital as the SOMO and the unpaired electron in radicals M and N reside in an orbital of π-symmetry.^42,47,87,88^

**Fig. 5:**
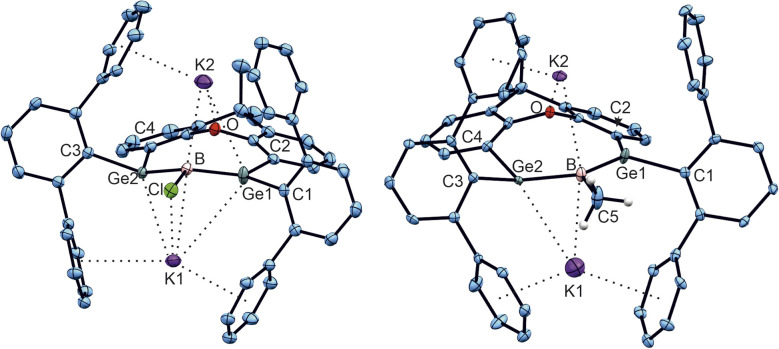
ORTEPs of the molecular structures of K_2_[1a] (left in figure) and K_2_[1b] (right in figure). Thermal ellipsoids are shown at 50% probability. *i*-Pr groups and hydrogen atoms except the hydrogen atoms of the B–Me unit have been omitted. Selected interatomic distances [Å] and angles [°]: K_2_[1a] Ge1–B 1.9454(19), Ge2–B 2.045(2), B–Cl 1.8725(19), K1–Cl 3.0399(6), K1–B 3.149(2), K1–Ge1 3.7423(5), K1–Ge2 3.3632(19), K1–C (arene) 3.2296(19) – 3.4542(19), K2–B 3.043(2), K2–Ge1 3.4547(5), K2–O 2.6807(13), K2–C (arene) 3.081(2) – 3.490(2), Ge1–B–Ge2 129.4(1), Ge1–B–Cl 110.6(1), Ge2–B–Cl 110.9(1); K_2_[1b] Ge1–B 1.962(5), Ge2–B 2.017(6), B–C5 1.609(7), K1–B 2.996(6), K1–Ge2 3.287(6), K1–C (arene) 3.007(6) – 3.472(5), K2–B 3.181(7), K2–O 2.645(6), K2–C (arene) 3.216(6) – 3.447(8), Ge1–B–Ge2 127.0(3), Ge1–B–C5 116.4(3), Ge2–B–C5 114.9(3).

Reduction of the radical anions (Fig. 3) with a further equivalent of KC_8_ yields the dianions K_2_[1a] and K_2_[1b] (Scheme 5). As the best preparative procedure for the syntheses of the dianions the reduction of the BCl_3_ and MeBBr_2_ insertion products B and 2 with four equivalents of KC_8_ at room temperature in diethyl ether was developed (Scheme 6). Crystallization was carried out at −40 °C in *n*-pentane to give dark green crystals of K_2_[1a] and K_2_[1b]. The molecular structures of the salts are depicted in Fig. 5 and further coordination of the potassium cations in aromatic moieties of the organic ligands and coordination at dianionic BGe_2_-units is shown. Both dianions feature a slight elongation of one of the Ge–B bonds [1a^2−^ Ge2–B 2.045(2), 1b^2−^ Ge2–B 2.017(6) Å] in comparison to the boradigermaallyl structures [1a 1.960(2), 1.962(2), 1b 1.962(4), 1.948(5) Å] which could possibly be due to the population of the nonbonding combination of the three aligned p-orbitals upon reduction (Fig. 3, right). The Ge1–B–Ge2 angle in 1a^2−^ of 129.4(1)° only slightly deviates from the corresponding radical [1a˙^−^ Ge1–B–Ge2 131.8(1)°]. Interestingly, the boron atom in 1a^2−^ shows a slight pyramidalization in the solid-state structure with a sum of bond angles around boron of 350.9°. Both salts show a signal in the ^11^B NMR spectrum at higher frequencies (K_2_[1a] 57.0, K_2_[1b] 55.5 ppm) in comparison to the boradigermaallyl derivatives (1a 42.4, 1b 41.9 ppm). On first sight, this observation may appear puzzling. However, quantum chemical calculations of magnetic shielding tensors of 1a and K_2_[1a] reproduce the experimental findings and indicate that this effect is caused by a significant deshielding contribution in σ_11_ along the direction of the B–Cl bond. This contribution arises from magnetic dipole allowed efficient mixing of the HOMO in 1a^2−^ with low lying σ* orbitals (see SI for more details). The excited state spectra of K_2_[1a] and K_2_[1b] were investigated by TD-DFT calculations (see SI). HOMO–LUMO + *n* (*n* = 1,2,3,4) transitions are in the visible range and are there assigned to cause the dark green colour of the compounds. It is worth noting, that due to the high sensitivity of K_2_[1a] and K_2_[1b] it is experimentally challenging to obtain meaningful UV-Vis spectra even from highest purity solvents upon dilution.

Oxidation of the dianions K_2_[1a] and K_2_[1b] to yield the radical anions K[1a] and K[1b] (Scheme 5) was achieved by reaction with one equivalent of boradigermaallyl 1a and 1b, respectively. Oxidation by treating the dianions K_2_[1a] and K_2_[1b] with two equivalents of iron pentacarbonyl led in both cases to the formation of 1a and 1b (Scheme 5).

**Fig. 6:**
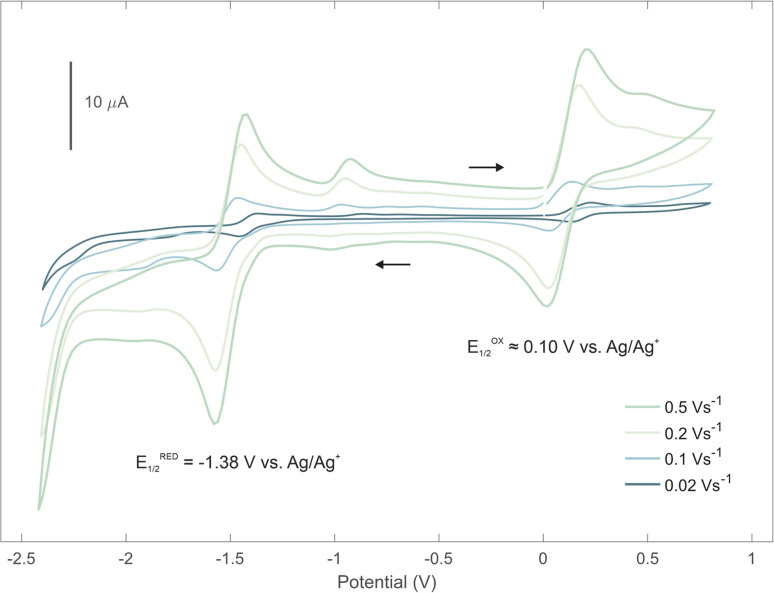
Cyclic voltammogram of 1a ([Bu_4_N][Al(OC{CF_3_}_3_)_4_] in THF) at various scan rates.

Electrochemical properties of the boradigermaallyl derivatives 1a and 1b were analyzed by cyclic voltammetry in THF using the silver/silver cation (Ag/Ag^+^) redox couple as reference and [Bu_4_N][Al(OC{CF_3_}_3_)_4_] salt as supporting electrolyte (Fig. 6, see SI for cyclic voltammogram of 1b).^95^ Both compounds reveal a reduction wave (1a −1.38 V, 1b −1.67 V) and an oxidation wave (1a 0.10 V, 1b 0.16 V).

The observed difference of reduction potentials is in accordance with the pronounced electron-withdrawing character of the chloro substituent. In the case of the reduction wave of 1a, which was obtained at different scan rates, a one electron transfer process can be estimated using the modified Randles-Ševčík equation for quasi-reversible processes (see SI).^96^ Redox potentials were computed at the r^2^SCAN-3c/CPCM(THF) level of theory analogously to a protocol published by Grimme and co-workers.^97^ Since the potassium counterion was not considered, the absolute values should be interpreted with caution. Nevertheless, the computed data clearly show a separation of the reduction potentials of redox couples 1|1˙^−^ and 1˙^−^|1^2−^ by approximately 0.5 V (see SI). This leads us to the conclusion that the recorded reduction wave corresponds to the reaction 1 + e^−^ ⇌ 1˙^−^ and not to a single-step two electron reduction. A second reduction step is not observed within the accessible electrochemical window, as the potential required exceeds the stability limit of THF below −2.2 V. The electrochemical oxidation of the low valent boron compounds 1a and 1b was investigated by preparative oxidation reactions with Ag[Al(OC{CF_3_}_3_)_4_], NOPF_6_ and [Cp_2_Fe][PF_6_]. However, we were not able to isolate any product of the oxidation.^98^

## Conclusion

In summary, methyl- and chloroborylene, stabilized by a chelating bis(diarylgermylene), form delocalized neutral allyl-cation analogues that exhibit stepwise two-electron reduction. After isolation of the persistent radical anioni and dianion as the products of reduction, their preparative oxidation confirms the interconvertible nature of the redox products. The radical boradigermaallyl species exhibit delocalization of the radical on the Ge–B–Ge moiety with spin densities comparable to the C_3_H_5_ allyl radical.

## Author contributions

Investigations, computational investigations, writing, review S. F. M.; special NMR experiments K. E.; X-ray measurements and structure determinations H. S.; supervision, funding acquisition, manuscript writing and review H. F. B.; electrochemistry, supervision, funding acquisition, manuscript writing and review C. P. S.; supervision, funding acquisition, manuscript writing and review L. W.

## Conflicts of interest

The authors declare no conflicts of interest.

## Supplementary Material

SC-017-D6SC00727A-s001

SC-017-D6SC00727A-s002

SC-017-D6SC00727A-s003

## Data Availability

Full experimental and computation details are provided as part of the supplementary information (SI). Supplementary information is available. See DOI: https://doi.org/10.1039/d6sc00727a. CCDC 2504370–2504374 contain the supplementary crystallographic data for this paper.^99*a*–*e*^
