# Supplementary material for: Three reversibly interconvertible redox states of boradigermaallyl: syntheses of radical allyl anion and allyl dianion
Source: Chem Sci. 2026 Mar 2;17(17):8622–9. doi: 10.1039/d6sc00727a (PMC12969875; doi:10.1039/d6sc00727a)
Supplement: SC-017-D6SC00727A-s001 [file SC-017-D6SC00727A-s001.pdf]

Supplementary Information (SI) for Chemical Science.
This journal is © The Royal Society of Chemistry 2026

Three Reversibly Interconvertible Redox States of Boradigermaallyl: Syntheses of Radical Allyl Anion and Allyl Dianion

Stefan F. Miehe,[a] Klaus Eichele,[a] Hartmut Schubert,[a] Holger F. Bettinger,[b] Christian P. Sindlinger*[c] and Lars Wesemann*[a]

[a]Institut für Anorganische Chemie, Eberhard Karls Universität Tübingen, Auf der Morgenstelle 18, 72076 Tübingen, Germany. E-mail: lars.wesemann@uni-tuebingen.de
[b]Institut für Organische Chemie, Eberhard Karls Universität Tübingen, Auf der Morgenstelle 18, 72076 Tübingen, Germany. E-mail: holger.bettinger@uni-tuebingen.de
[c]Institut für Anorganische Chemie, Universität Stuttgart, Pfaffenwaldring 55, 70569 Stuttgart, Germany. E-mail: christian.sindlinger@iac.uni-stuttgart.de

Supporting Information **A**

Content

Experimental 2
Synthesis 3
Crystal structure determination 7
NMR spectroscopy 8
- NMR spectra of compound **2** 8
- NMR spectra of compound **1b**. 11
- NMR spectra of compound K_2[**1a**]. 14
- NMR spectra of compound K_2[**1b**]. 17
- Comparison of the ^{11}B NMR chemical shifts of **1a** and **1a**$^{2-}$. 20

UV-Vis spectroscopy 25
EPR spectroscopy 40
Cyclic voltammetry 43
Number of electrons transferred during reduction of **1a** 45
Quantum chemistry 45
- Structure optimization/NBO analysis 45
- Computational study on EPR parameters of K[**1**$^{\cdot}$] 46
- Spin density distribution and Mulliken atomic spin densities of K[**1a**$^{\cdot}$] 50
- NMR shielding tensor computations 50
- Frontier orbital energies of **1**, **K[1]** and **K_2[1]** 51
- Redox Potentials 52

References 53

Experimental

General procedures: All manipulations were carried out under argon atmosphere using standard Schlenk techniques and gloveboxes. Benzene was dried with activated aluminium oxide, *n*-pentane and *n*-hexane were obtained from a MBraun solvent purification systems (SPS). All other solvents (Et_2O, THF, toluene, benzene-d_6, cyclohexane-d_{12}) were distilled from a sodium-potassium alloy and like the previous mentioned solvents subsequently degassed by three freeze−pump−thaw cycles. Bis(germylene) **A**, BCl_3-addition product **B**, boradigermaallyl **1a**, and $MeBBr_2{\cdot}SMe_2$ were prepared according to literature procedures.[1, 2] Further chemicals were purchased commercially and used as received.

Elemental analysis: Elemental analysis was performed at the Institute of Inorganic Chemistry, University of Tübingen using an *elementar* vario MICRO Cube.

NMR spectroscopy: NMR spectra were recorded with either a Bruker Avance III HD 300 NanoBay spectrometer equipped with a 5 mm BBFO probe head and operating at 300.13 (^1H) and 96.29 (^{11}B) MHz, a Bruker Avance II+ 400 spectrometer with a Quad Systems 5 mm BBFO probe head operating at 400.11 (^1H) and 100.61 (^{13}C) MHz, a Bruker Avance III HDX 600 spectrometer equipped with a 5 mm Prodigy BBO cryo probe head operating at 600.13 (^1H) and 150.90 (^{13}C) or a Bruker Avance III HDX 700 NMR spectrometer equipped with a 5 mm TXI probe head operating at 700.29 (^1H) and 176.9 (^{13}C) MHz. Chemical shifts are reported in δ values in ppm relative to external $SiMe_4$ (^1H, ^{13}C) or $BF_3 \cdot OEt_2$ (^{11}B) referenced in most cases on the ^2H resonance frequency of the solvent as follows: Ξ = 25.145020 % for ^{13}C, Ξ = 32.083 974 % for ^{11}B. The multiplicity of the signals is indicated as s = singlet, d = doublet, t = triplet, sept = septet, m = multiplet or br = broad/unresolved. For the assignment of proton and carbon signals detailed analysis of ^1H, $^{13}C\{^1H\}$, ^1H–^1H COSY, ^1H–^{13}C HSQC, ^1H–^{13}C HMBC, and $^{13}C\{^1H\}$ DEPT 135 spectra was done.

Cyclovoltammetry: Cyclovoltammetric measurements were performed with a Metrohm Autolab PGStat100 potentiostat using a glassy carbon working electrode, a Pt-wire as counter electrode and a silver-wire (freshly treated with aqueous NaOCl solution, rinsed with dest water and dried shortly prior to the experiment) as pseudo-reference electrode in 0.05M THF solutions of $[NBu_4][Al(OtBu^F)_4]$[3] in a custom-made electrochemical cell (1.5 mL sample volume, 1cm vial diameter) kept within a glovebox. THF was freshly dried over $LiAlH_4$ and vacuumtransferred/condensed. Due to great sensitivity and reactivity of the probed sample application of internal reference systems such $FeCp_2/FeCp_2^+$ remained unsuccesful.

Crystallography: X-ray data were collected with a Bruker Smart APEX II diffractometer with graphite-monochromated Mo-K_α radiation. The programs used were Bruker's APEX2 v2011.8-0, including SAINT for data reduction, SADABS for absorption correction, and SHELXS for structure solution, as well as the WinGX suite of programs version 1.70.01 or the GUI ShelXle, including SHELXL for structure refinement. [4-10]

UV/Vis Spectroscopy: Visible UV/Vis absorption spectra were recorded on PerkinElmer Lambda 35 spectrophotometer in gas tight 1 cm quartz cuvettes sealed with Teflon stoppers or Teflon lined screw caps.

Synthesis

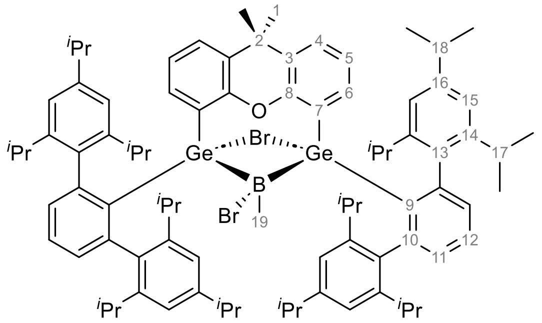

2: Bis(germylene) **A** (200 mg, 15.2 µmol, 1.00 eq.) is dissolved in *n*-pentane (15.0 mL). $MeBBr_2 \cdot SMe_2$ (37.6 mg, 15.2 µmol, 1.00 eq.) is added to the orange solution and the reaction mixture is stirred for 2 h at RT. When the solution turns bright yellow, the reaction is completed. If necessary, some $MeBBr_2 \cdot SMe_2$ can be added to achieve a complete reaction. The solution is filtered and concentrated to about half of the initial volume. After 30 min at RT small crystals begin to form, if not, the solution needs to be further concentrated. The product **2** is obtained by fractional crystallization over night at RT from *n*-pentane as pale yellow to colourless crystals (200 mg, 13.3 µmol, 88%), which are suitable for X-ray crystal structure analysis. *Analytical data:* ^{1}H-NMR (400.11MHz, C_6D_6): *δ* [ppm] = -0.59 (s, 19–C*H*$_3$), 0.20-2.00 (1H, 1–*H*, 17– C*H*$_3$ + 18–C*H*$_3$), 2.80-3.50 (17–*H* + 18–*H*), 6.4-7.4 (4-6–*H*, 11–*H*, 12–*H*, 15–*H*). *signals could not be assigned, as compound* **2** *is found to exist as two diastereomers (41:59).* ^{11}B{^{1}H}-NMR (96.29MHz, C_6D_6): *δ* [ppm] = 14.0 (br.s). Elemental analysis for $C_{88}H_{113}OGe_2BBr_2$: 70.57% C (calc. 70.34%), 7.24% H (calc. 7.58%).

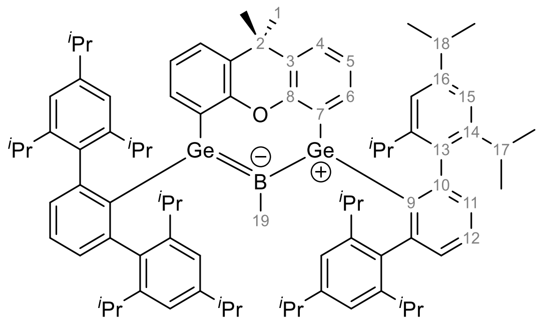

1b: **2** (200.0 mg, 133 µmol, 1.00 eq.) is dissolved in diethyl ether (10.0 mL). KC_8 (36.0 mg, 266 µmol, 2.00 eq.) is added to the pale-yellow reaction solution at RT and the reaction mixture is stirred for 30 min. The solution first turns green and then intensely blue. The solvent is removed, and the sample is placed under dynamic vacuum for 4 hours. The product is extracted with n-pentane and graphite as well as KBr are filtered off. The product **1b** is obtained by fractional crystallization over night at -40 °C from *n*-pentane as blue crystals (147.0 mg, 109 µmol, 82.2%). Crystals suitable for X-ray crystal structure analysis can be obtained at -40 °C from *n*-pentane. *Analytical data:* ^{1}H-NMR (400.11MHz, C_6D_6): *δ* [ppm] = 0.51 (d, 12H, $^{3}J_{HH}$ = 6.7 Hz, 17–C*H*$_3$), 0.57 (s, 3H, 19-C*H*$_3$), 1.06 (d, 12H, $^{3}J_{HH}$ = 6.7 Hz, 17–C*H*$_3$), 1.19-1.27 (m, 18H, 17–C*H*$_3$ + 1–*H*), 1.32-1.41 (m, 24H, 17– C*H*$_3$ + 18–C*H*$_3$ + 18–C*H*$_3$), 2.99 (sept, 4H, $^{3}J_{HH}$ = 6.9 Hz, 18–*H*), 3.04-3.16 (m, 8H, 17–*H* + 17–*H*), 6.50-6.59 (m, 2H, 5–*H*), 6.63 (dd, 2H, $^{3}J_{HH}$ = 7.5 Hz, $^{4}J_{HH}$ = 1.3 Hz, 6–*H*), 6.79 (dd, 2H, $^{3}J_{HH}$ = 7.5 Hz, $^{4}J_{HH}$ = 1.2 Hz, 4–*H*), 7.01 (d, 4H, $^{3}J_{HH}$ = 1.7 Hz, 15–*H*), 7.15-7.21 (m, 6H, 15–*H* + 11–*H*), 7.23-7.30 (m, 2H, 12–*H*); ^{13}C{^{1}H}-NMR (100.61MHz, C_6D_6): *δ* [ppm] = 6.0 (C19), 23.4 (17–*C*H$_3$), 23.8 (17–*C*H$_3$), 25.0 (18–*C*H$_3$), 25.1 (18–*C*H$_3$), 26.9 (17–*C*H$_3$), 27.4 (17–*C*H$_3$+C1), 31.3 (C17), 31.7 (C17), 35.2 (C18), 37.1 (C2), 121.5 (C15), 121.9 (C15), 124.5 (C5), 125.2 (C4), 127.9 (C11), 128.9 (C11), 131.1 (C12), 131.2 (C6), 133.1 (C3+C10), 136.0 (C7), 139.0 (C13), 147.1 (C14), 147.3 (C9), 148.9 (C14), 149.0 (C16), 149.4 (C10), 158.2 (C8); ^{11}B{^{1}H}-NMR (96.29MHz, C_6D_6): *δ* [ppm] = 41.9 (br.s). Elemental analysis for $C_{88}H_{113}OGe_2B$: 78.79% C (calc. 78.71%),

8.17% H (calc. 8.48%). UV-vis (Et_2O, c = 0.149 mmol·L^{-1}): λ_{max} /nm (ε /L·mol^{-1}·cm^{-1}): 362 (6.0·10^3), 478 (3.3·10^3), 636 (8.7·10^3).

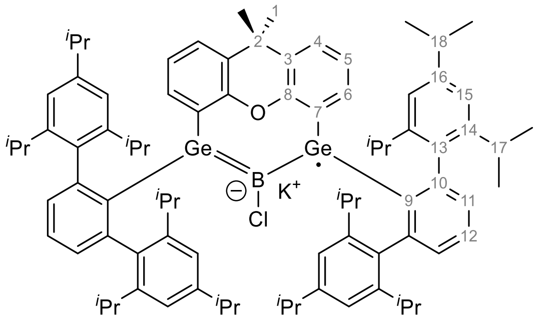

K[**1a**•]: **B** (50.0mg, 34.9 µmol, 1.00 eq.) is dissolved in diethyl ether (10.0 mL). KC_8 (14.1 mg, 10.5 µmol, 3.00 eq.) is added to the pale-yellow reaction solution and is stirred for 30 min at RT. The solution first turns green followed by a change of colour to dark blue. The solvent is removed, and the sample is placed under dynamic vacuum for 4 hours. The product is extracted with *n*-pentane (2 mL) and graphite and KCl are filtered off. The product is obtained by fractional crystallization over several days at -40 °C from diethylether in the form of navy blue crystals (45.6 mg, 34.9 µmol, 94.3%). Black-blue crystals suitable for X-ray crystal structure analysis can be obtained at -40 °C from diethyl ether. *Alternative synthesis:* K[**1a**•] is as well accessible *via* reduction of **1a** with KC_8 (1 eq.) under identical reaction conditions, followed by the same workup as in the main synthesis. *Analytical data:* cw-EPR (9.321653 GHz, Et_2O): see EPR spectroscopy. Elemental analysis for $C_{87}H_{110}OGe_2BClK$: 75.01% C (calc. 74.51%), 7.76% H (calc. 7.91%). UV-vis (Et_2O, c = 0.029 mmol·L^{-1}): λ /nm (ε /L·mol^{-1}·cm^{-1}): 375 (2.5·10^3), 606 (2.4·10^3). The solution magnetic susceptibility could not be evaluated using Evans Method, due to a very small peak separation.

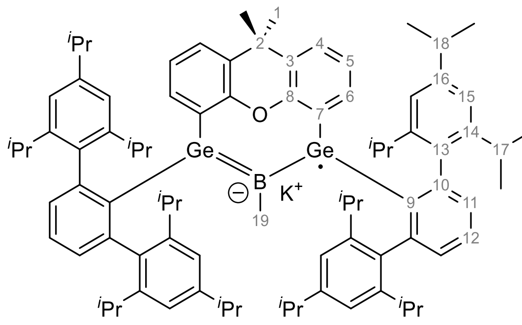

K[**1b**•]: **2** (50.0 mg, 33.3 µmol, 1.00 eq.) is dissolved in diethyl ether (10.0 mL). KC_8 (13.5 mg, 99.8 µmol, 3.00 eq.) is added to the pale-yellow reaction solution and is stirred for 30 min at RT. The solution first turns green followed by a change of colour to dark blue. The solvent is removed, and the sample is placed under dynamic vacuum for 4 hours. The product is extracted with *n*-pentane (2 mL) and graphite and KBr are filtered off. The solvent is removed to obtain product K[**1b**•] (42.8 mg, 30.9 µmol, 93.0%). Navy blue crystals suitable for X-ray crystal structure analysis can be obtained at -40 °C from diethyl ether. *Alternative synthesis:* K[**1b**•] is as well accessible *via* reduction of **1b** with KC_8 (1 eq.) under identical reaction conditions, followed by the same workup as in the main synthesis. *Analytical data:* cw-EPR (9.321653 GHz, Et_2O): see EPR spectroscopy. Elemental analysis for $C_{88}H_{113}OGe_2BK$: 76.58% C (calc. 76.48%), 8.23% H (calc. 8.24%). UV-vis (Et_2O, c = 0.029 mmol·L^{-1}): λ /nm (ε /L·mol^{-1}·cm^{-1}): 404 (2.9·10^3), 650 (3.6·10^3).

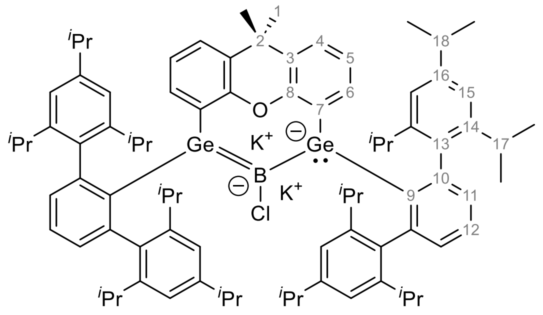

K$_2$[**1a**]: **B** (50.0mg, 34.9 µmol, 1.00 eq.) is dissolved in diethyl ether (10.0 mL). KC$_8$ (18.9mg, 139.0 µmol, 4.00 eq.) is added to the pale-yellow reaction solution and is stirred for 30 min at RT. The solution first turns green, then dark blue and finally dark green. The solvent is removed, and the sample is placed under dynamic vacuum for 4 hours. The product K$_2$[**1a**] is extracted with *n*-pentane (2 mL) and graphite and KCl are filtered off. The product is obtained by fractional crystallization over several days at -40 °C from *n*-pentane in the form of dark green crystals (42.5 mg, 34.9 µmol, 85.8%). Black-green crystals suitable for X-ray crystal structure analysis can be obtained at –40 °C from *n*-pentane. *Alternative synthesis:* K$_2$[**1a**] is as well accessible *via* reduction of **1a**/K[**1a**$^{\cdot}$] with KC$_8$ (2/1 eq.) under identical reaction conditions, followed by the same workup as in the main synthesis. *Analytical data:* ^{1}H-NMR (400.11MHz, C$_6$*D*$_{12}$): δ [ppm] =0.30 (d, 12H, $^{3}J_{HH}$ = 6.66 Hz, 17–C*H*$_3$), 0.75 (d, 12H, $^{3}J_{HH}$ = 0.76 Hz, 17–C*H*$_3$), 0.98 (d, 12H, $^{3}J_{HH}$ = 6.78 Hz, 17–C*H*$_3$), 1.16-1.33 (m, xH, 17–C*H*$_3$ + 18–C*H*$_3$ + 1–*H* + 18–C*H*$_3$), 2.83 (sept, 4H, $^{3}J_{HH}$ = 6.86 Hz, 18–*H*), 2.96-3.15 (m, 8H, 17–*H* + 17–*H*), 6.27-6.42 (dd, 2H, 6–*H*), 6.50-6.57 (m, 2H, 5–*H*), 6.62-6.68 (dd, 2H, 4–*H*), 6.76 (d, 4H, $^{3}J_{HH}$ = 1.65 Hz, 15–*H*), 6.85-6.93 (m, 8H, 11–*H* + 15–*H*), 7.02-7.11 (m, 2H, 12–*H*). ^{13}C{^{1}H}-NMR (100.61MHz, C$_6$*D*$_{12}$): δ [ppm] = 22.9 (17–*C*H$_3$), 24.1 (17–*C*H$_3$), 24.6 (18–*C*H$_3$), 25.1 (18–*C*H$_3$), 25.7 (17–*C*H$_3$), 26.0 (17–*C*H$_3$), 28.6 (C1), 30.9 (C17), 31.7 (C17), 34.9 (C18),36.5 (C2), 117.9 (C6), 120.0 (C15), 122.9 (C5), 124.7 (C12), 129.9 (C11), 131.4 (C3), 132.9 (C4), 143.5 (C7), 145.4 (C9), 146.4 (C16), 149.1 (C10), 150.0 (C14), 150.1 (C14), 154.1 (C9), 154.3 (C8). ^{11}B{^{1}H}-NMR (96.29MHz, C$_6$*D*$_{12}$): 57.0 (br.s). Elemental analysis for C$_{87}$H$_{110}$OGe$_2$BClK$_2$: 72.61% C (calc. 72.49%), 7.66% H (calc. 7.69%). UV-vis (Et$_2$O, c = 0.028 mmol·L^{-1}): λ /nm (ε /L·mol^{-1}·cm^{-1}): 426 (13.3·10^{3}).

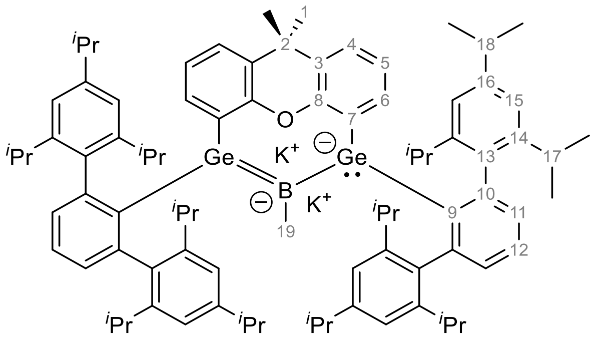

K$_2$[**1b**]: GeBMeBr$_2$Ge (50.0 mg, 33.3 µmol, 1.00 eq.) is dissolved in diethyl ether (10.0 mL). KC$_8$ (18.00 mg, 133 µmol, 4.00 eq.) is added to the pale-yellow reaction solution and is stirred for 30 min at RT. The solution first turns green, then dark blue and finally dark green. The solvent is removed, and the sample is placed under dynamic vacuum for 4 hours. The product K$_2$[**1b**] is extracted with *n*-pentane (2 mL) and graphite and KBr are filtered off. The product solvent is removed to obtain product K$_2$[**1b**] (44.8 mg, 31.5 µmol, 94.7%). Dark green crystals suitable for X-ray crystal structure analysis can be obtained at -40 °C from *n*-pentane. *Alternative synthesis:* K$_2$[**1b**] is as well accessible *via* reduction of **1b**/K[**1b**$^{\cdot}$] with KC$_8$ (2/1 eq.) under identical reaction

conditions, followed by the same workup as in the main synthesis. *Analytical data:* ^{1}H-NMR (400.11MHz, C_6D_{12}): δ [ppm] = 0.31 (d, 12H, $^{3}J_{HH}$ = 6.6 Hz, 17–C*H*$_3$), 0.78 (d, 12H, $^{3}J_{HH}$ = 6.7 Hz, 17–C*H*$_3$), 0.84 (s, 3H, 19–*H*), 0.99 (d, 12H, $^{3}J_{HH}$ = 6.8 Hz, 17–C*H*$_3$), 1.17-1.25 (m, 30H, 17–C*H*$_3$ + 18–C*H*$_3$ + C1), 1.30 (d, 12H, $^{3}J_{HH}$ = 6.9 Hz, 18–C*H*$_3$), 2.83 (sept, 4H, $^{3}J_{HH}$ = 6.9 Hz, 18–*H*), 2.96-3.14 (m, 8H, 17–*H* + 17–*H*), 6.30-6.4 (dd, 2H, $^{3}J_{HH}$ = 0.7 Hz, $^{4}J_{HH}$ = 7.3 Hz, 6–*H*), 6.44-6.52 (m, 2H, 5–*H*), 6.54-6.59 (dd, 2H, $^{3}J_{HH}$ = 1.2 Hz, $^{4}J_{HH}$ = 7.4 Hz, 4–*H*), 6.76 (d, 4H, $^{3}J_{HH}$ = 1.6 Hz, 15–*H*), 6.88 (d, 4H, $^{3}J_{HH}$ = 1.6 Hz, 15–*H*), 6.92 (d, 4H, $^{3}J_{HH}$ = 7.5 Hz, 11–*H*), 7.05-7.10 (m, 2H, 12–*H*). ^{13}C{^{1}H}-NMR (100.61MHz, C_6D_{12}): δ [ppm] =11.8 (C19), 23.1 (17–*C*H$_3$), 23.8 (17–*C*H$_3$), 24.5 (18–*C*H$_3$), 25.2 (18–CH$_3$), 25.8 (17–CH$_3$), 26.1 (17–*C*H$_3$), 28.5 (C1), 31.0 (C17), 31.7 (C17), 34.9 (C18), 36.4 (C2), 116.9 (C4), 119.9 (C15), 120.0 (C15), 122.8 (C5), 124.4 (C12), 129.7 (C11), 131.4 (C3), 132.6 (C6), 143.5 (C7), 145.6 (C13), 146.2 (C16), 149.1 (C14), 149.3 (C10), 150.1 (C14), 154.1 (C8), 155.7 (C9). ^{11}B{^{1}H}-NMR (96.29MHz, C_6D_{12}): 55.5 (br.s). Elemental analysis for $C_{88}H_{113}OGe_2BK_2$: 74.22% C (berechnet 74.38%), 7.86% H (berechnet 8.01%). UV-vis (Et_2O, c = 0.028 mmol·L^{-1}): *λ* /nm (ε /L·mol^{-1}·cm^{-1}): 343 (2.9·10^{3}), 650 (1.4·10^{3}).

Crystal structure determination

Table SI1. Data of crystal structure determinations.

	**2**	**1b**	K[**1a·**]	K_2[**1a**]	K_2[**1b**]
empirical formula	$C_{88}H_{113}Ge_2OBBr_2$	$C_{96}H_{133}Ge_2O_3B$	$C_{95}H_{130}BClGe_2KO_3$	$C_{97}H_{134}Ge_2OBClK_2$	$C_{88}H_{113}Ge_2OBK_2$
M [g/mol]	1502.59	1491.01	1550.52	1585.67	1420.97
T [K]	100(2)	150(2)	100(2)	120(2)	100(2)
λ [Å]	0.71073	0.71073	0.71073	0.71073	0.71073
crystal system	triclinic	triclinic	monoclinic	triclinic	triclinic
space group	$P\bar{1}$	$P\bar{1}$	$P2_1/n$	$P\bar{1}$	$P\bar{1}$
Z	2	2	4	2	2
a [Å]	13.9201(3)	14.16(3)	13.9575(3)	15.3084(4)	12.4223(3)
b [Å]	14.5139(3)	15.57(3)	24.6291(4)	15.6697(4)	13.6169(3)
c [Å]	22.3865(5)	23.12(4)	25.5330(5)	20.0999(5)	26.2587(6)
α [°]	82.6770(10)	95.88(10)	90	80.7170(10)	93.5190(10)
β [°]	71.9370(10)	106.07(13)	98.9240(10)	86.3170(10)	98.1790 (10)
γ [°]	65.7300(15)	111.83(10)	90	72.3980(10)	116.4050(16)
V [Å^3]	3919.92(15)	4424(14)	8671.0(3)	4535.0(2)	3898.77(16)
D_c [g/cm^3]	1.273	1.119	1.188	1.161	1.210
μ [mm^{-1}]	1.831	0.726	0.820	0.828	0.922
F(000)	1576	1604	3316	1696	1512
crystal size [mm]	0.28 x 0.27x 0.25	0.32 x 0.30 x 0.29	0.33 x 0.31 x 0.29	0.32 x 0.27 x 0.25	0.33 x 0.31 x 0.29
θ range [°]	1.539 – 28.995	1.595 – 28.850	1.569 – 29.512	2.234 – 28.344	1.583 – 26.372
limiting indices	−18 ≤ h ≤ 18<br>−19 ≤ k ≤ 19<br>−30 ≤ l ≤ 30	−18 ≤ h ≤ 19<br>−21 ≤ k ≤ 21<br>−29 ≤ l ≤ 31	−16 ≤ h ≤ 19<br>−34 ≤ k ≤ 34<br>−35 ≤ l ≤ 30	−20 ≤ h ≤ 20<br>−20 ≤ k ≤ 20<br>−24 ≤ l ≤ 26	−15 ≤ h ≤ 15<br>−17 ≤ k ≤ 17<br>−32 ≤ l ≤ 32
reflections collected	130134	131330	180203	189066	132491
independent reflections	20509	22867	23971	22161	15918
R_{int}	0.0321	0.0583	0.0352	0.0342	0.0219
Completeness [%]	99.6	99.6	99.1	99.6	99.6
absorption correction	multi-scan	multi-scan	multi-scan	multi-scan	multi-scan
max., min. transmission	0.6449/0.9799	0.6591/0.9799	0.6707/0.7459	0.7081/0.9799	0.6979/0.9799
parameter/restraints	888 /0	998/115	1057/452	1067/495	893/0
R_1, ωR_2 [I > 2σ(*I*)]	0.0249, 0.0604	0.0467, 0.1112	0.0563, 0.1363	0.0392, 0.1017	0.0786, 0.1826
R_1, ωR_2 (all data)	0.0315, 0.0626	0.0731, 0.1231	0.0744, 0.1460	0.0529, 0.1091	0.0805, 0.1835
GooF on F^2	1.004	1.077	1.063	1.026	1.291
peak / hole [e·Å^{-3}]	0.458, −0.328	1.575, −0.697	1.310, −1.965	1.069, −0.981	0.545, −2.558
CCDC	2504374	2504371	2504372	2504370	2504373

NMR spectroscopy

NMR spectra of compound **2**.

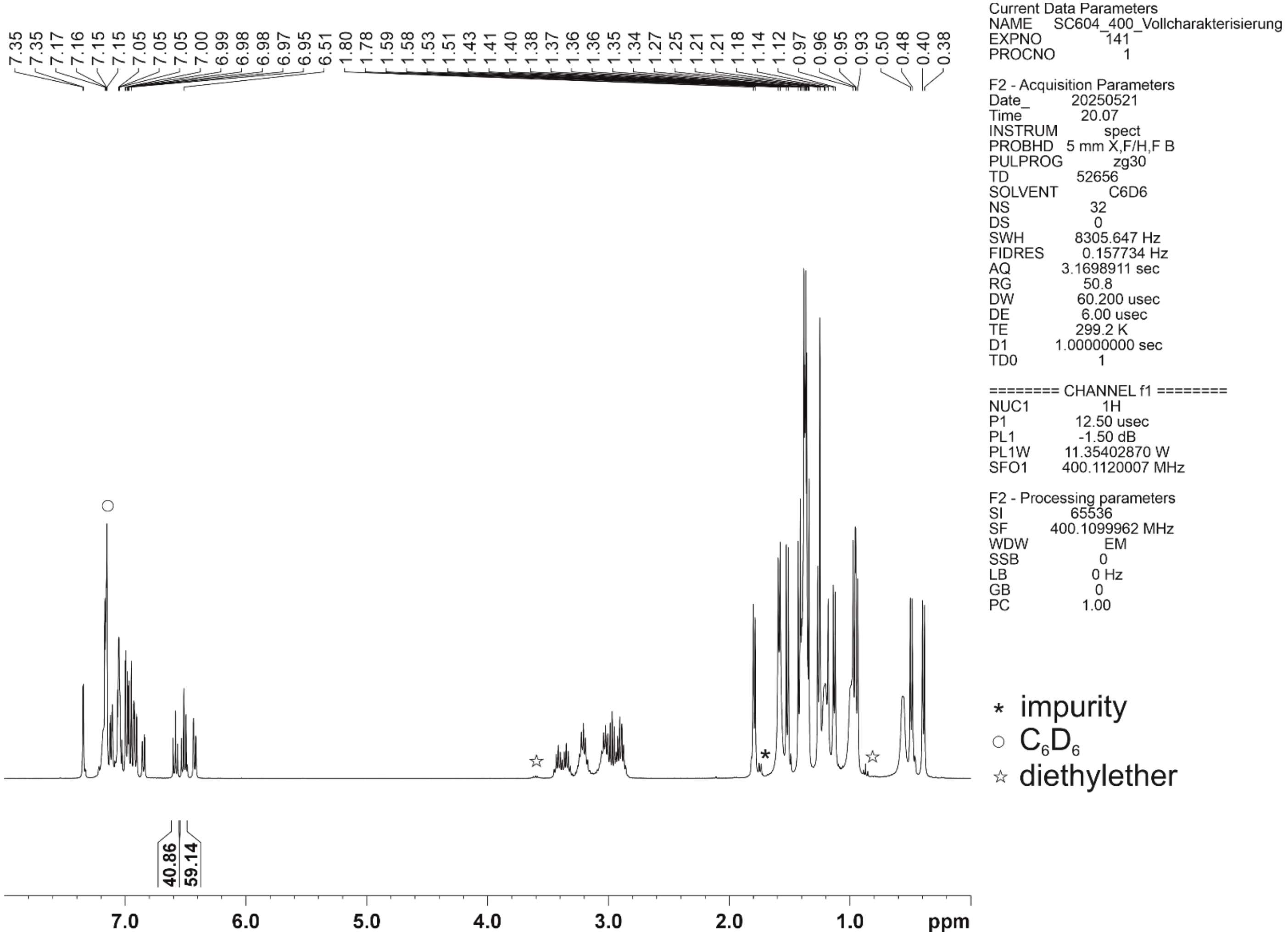

Figure SI1. ^{1}H NMR of compound **2**.

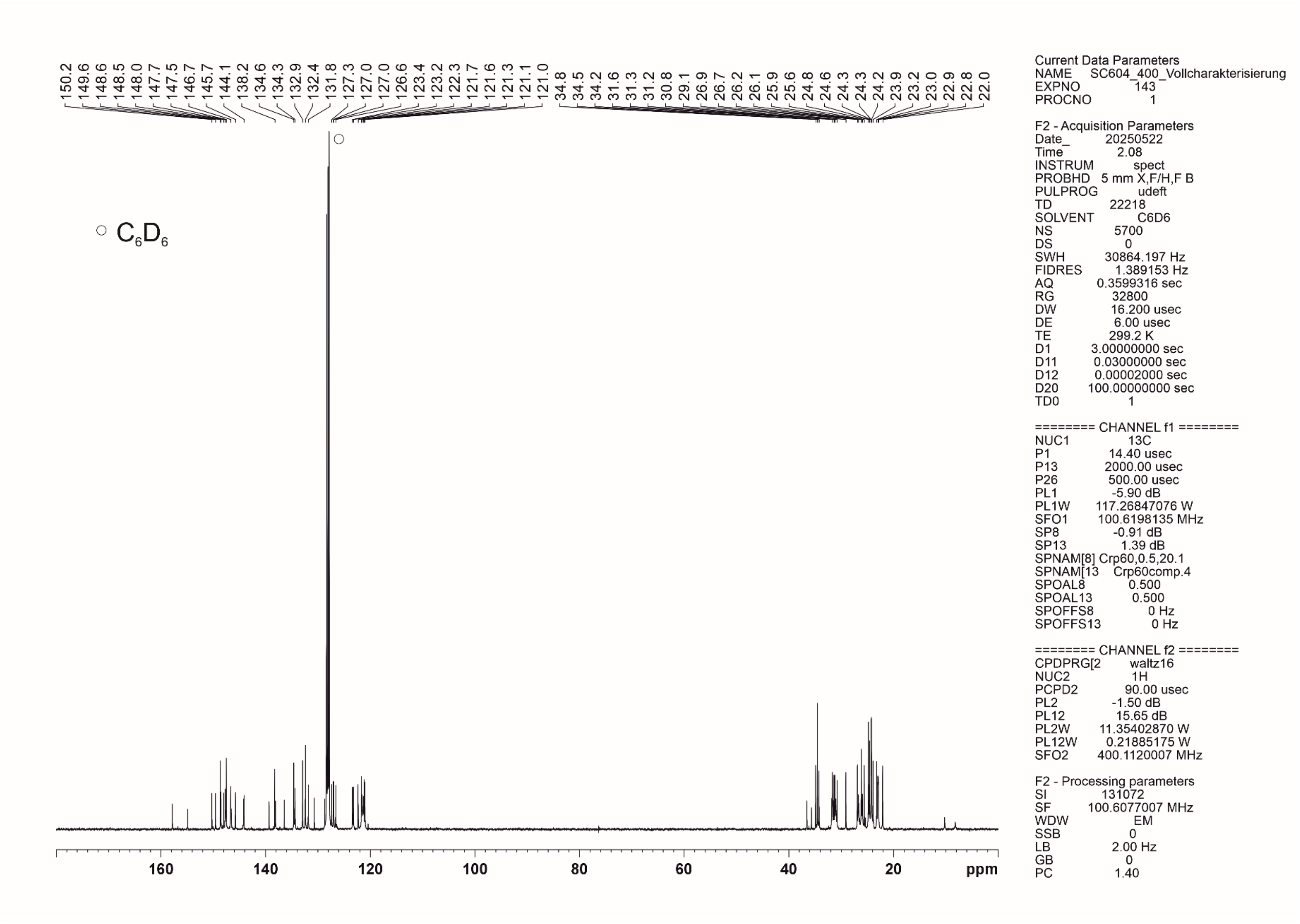

Figure SI2. $^{13}C\{^{1}H\}$ NMR of compound **2**.

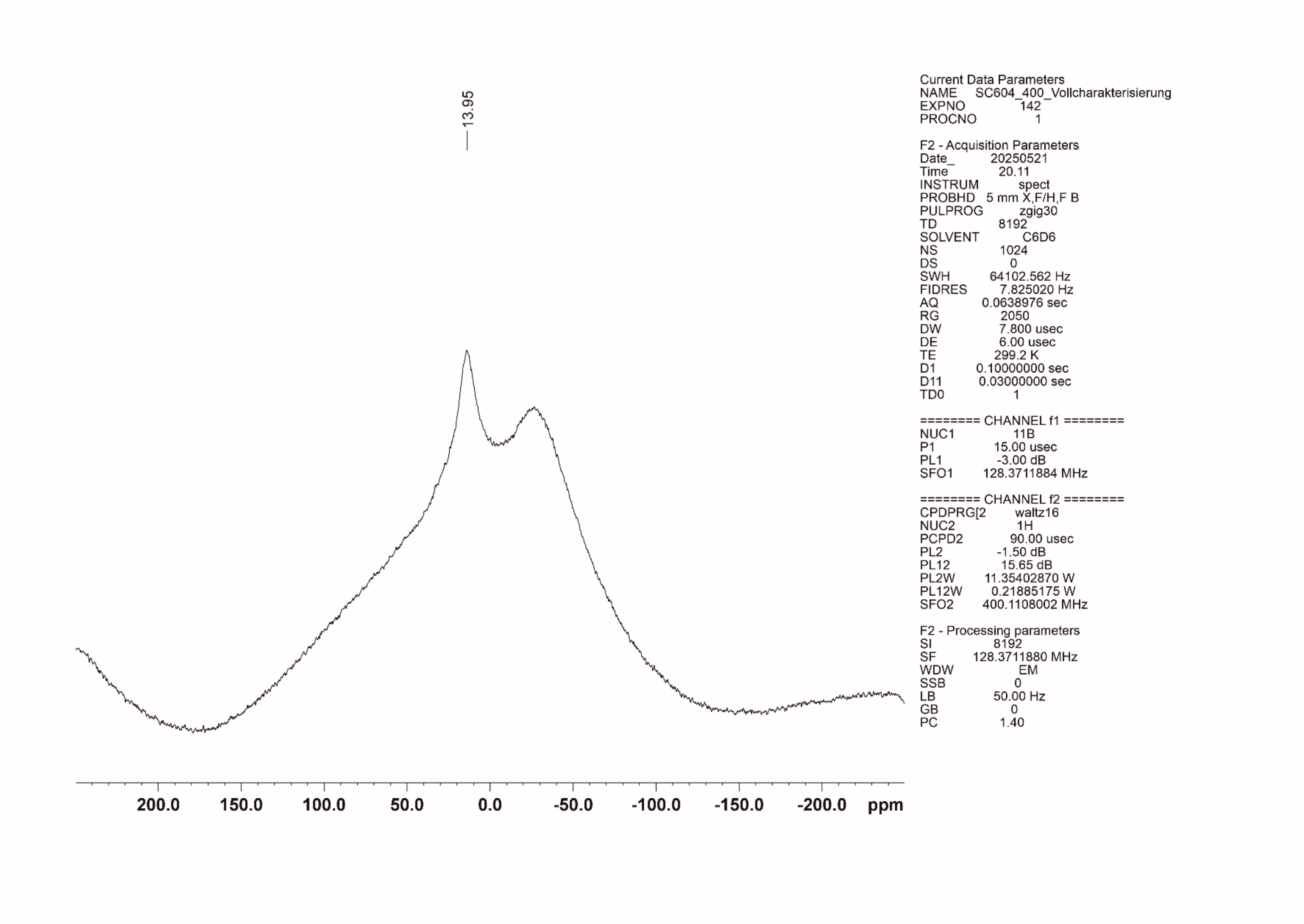

Figure SI3. $^{11}B\{^1H\}$ NMR of compound **2**.

NMR spectra of compound **1b**.

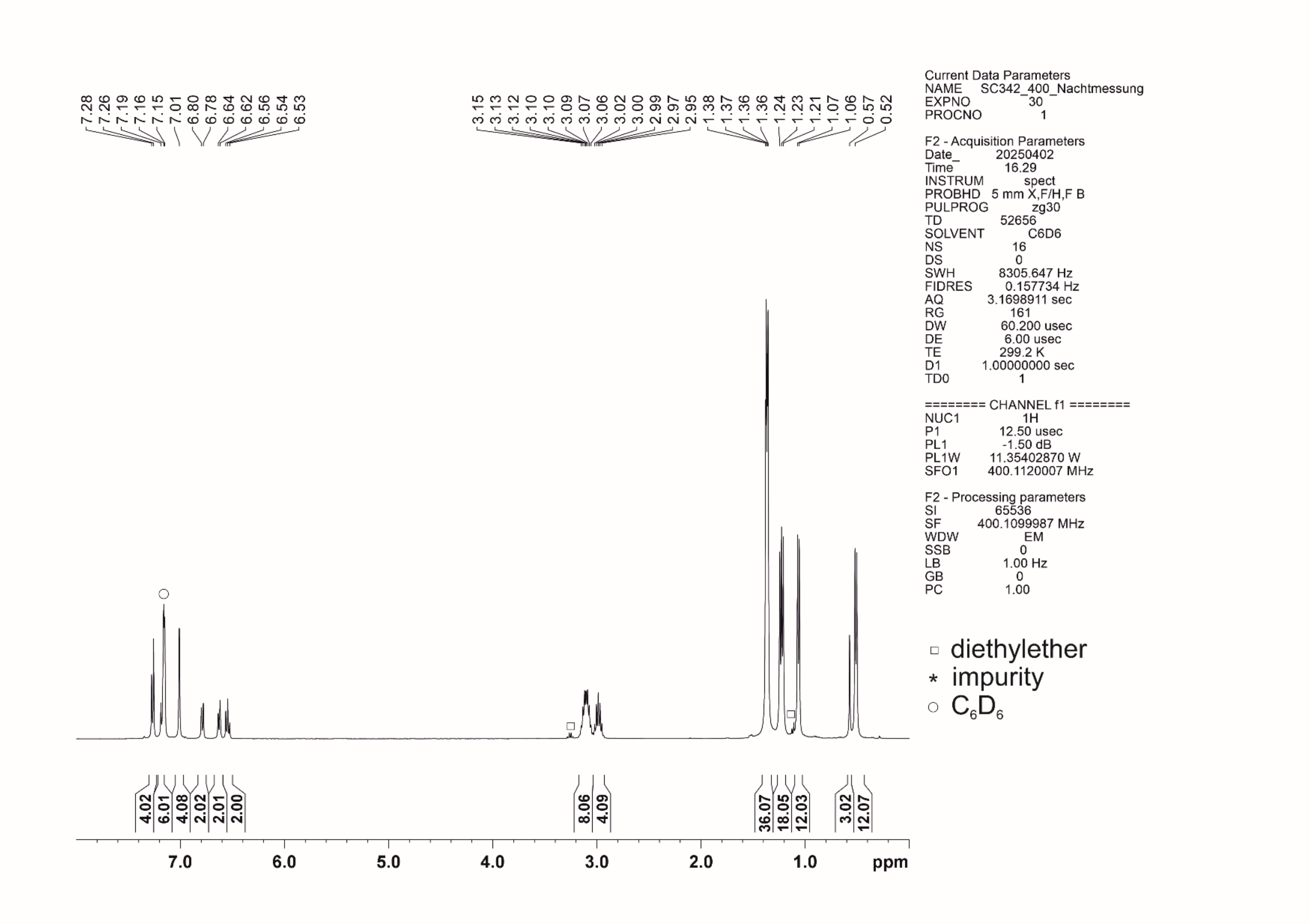

Figure SI4. ^{1}H NMR of compound **1b**.

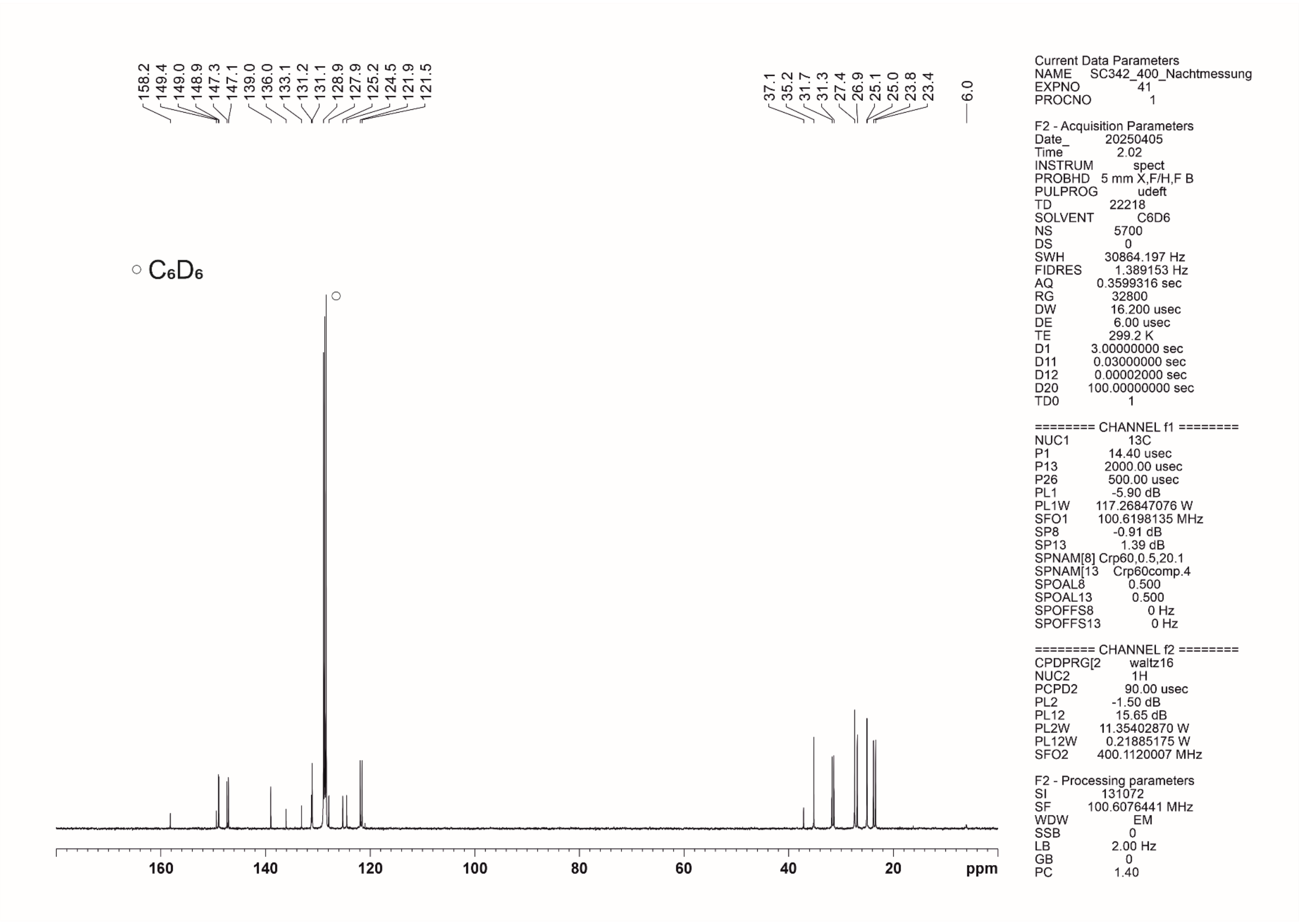

Figure SI5. $^{13}C\{^1H\}$ NMR of compound **1b**.

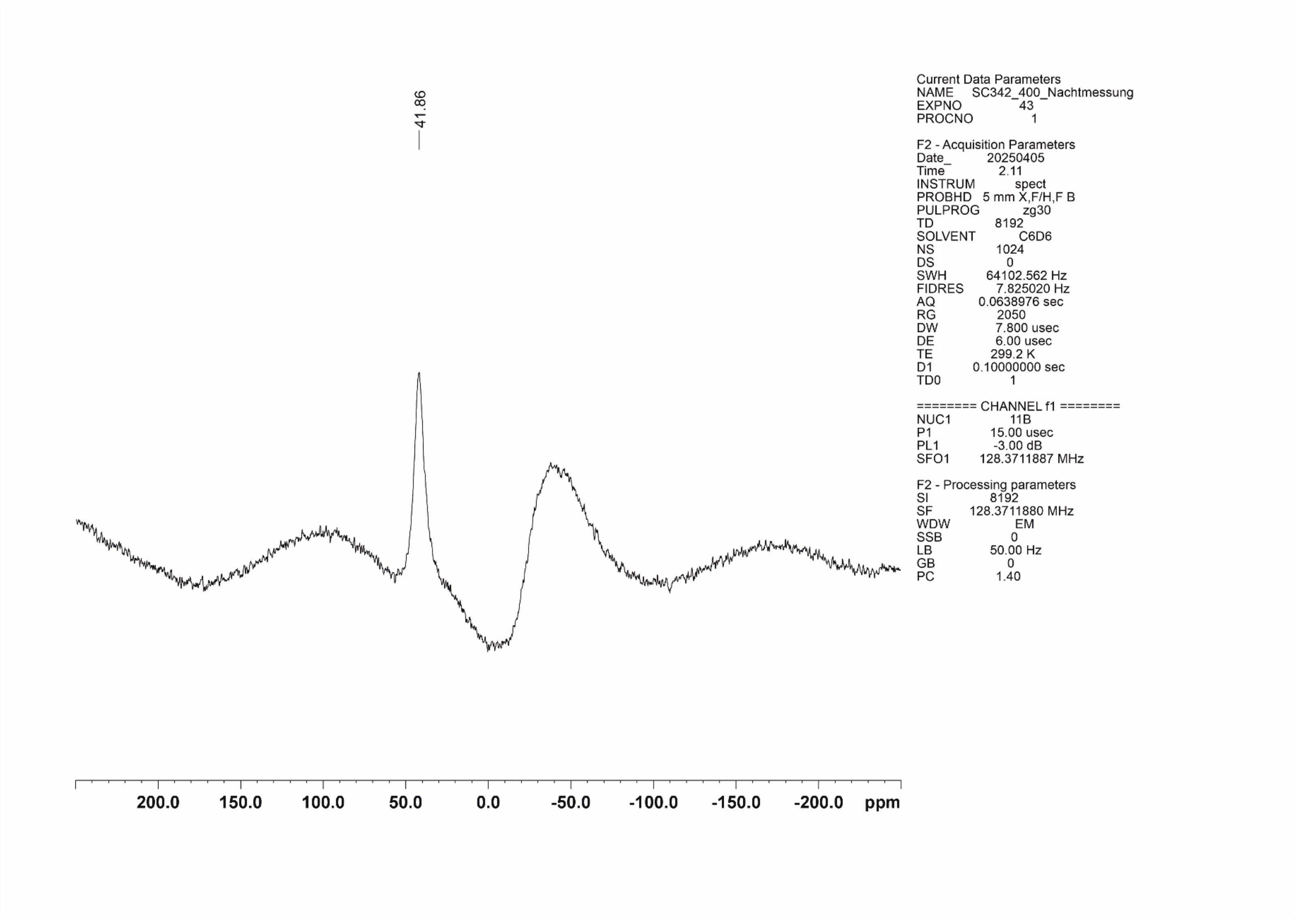

Figure SI6. $^{11}B\{^{1}H\}$ NMR of compound **1b**.

NMR spectra of compound K_2[**1a**].

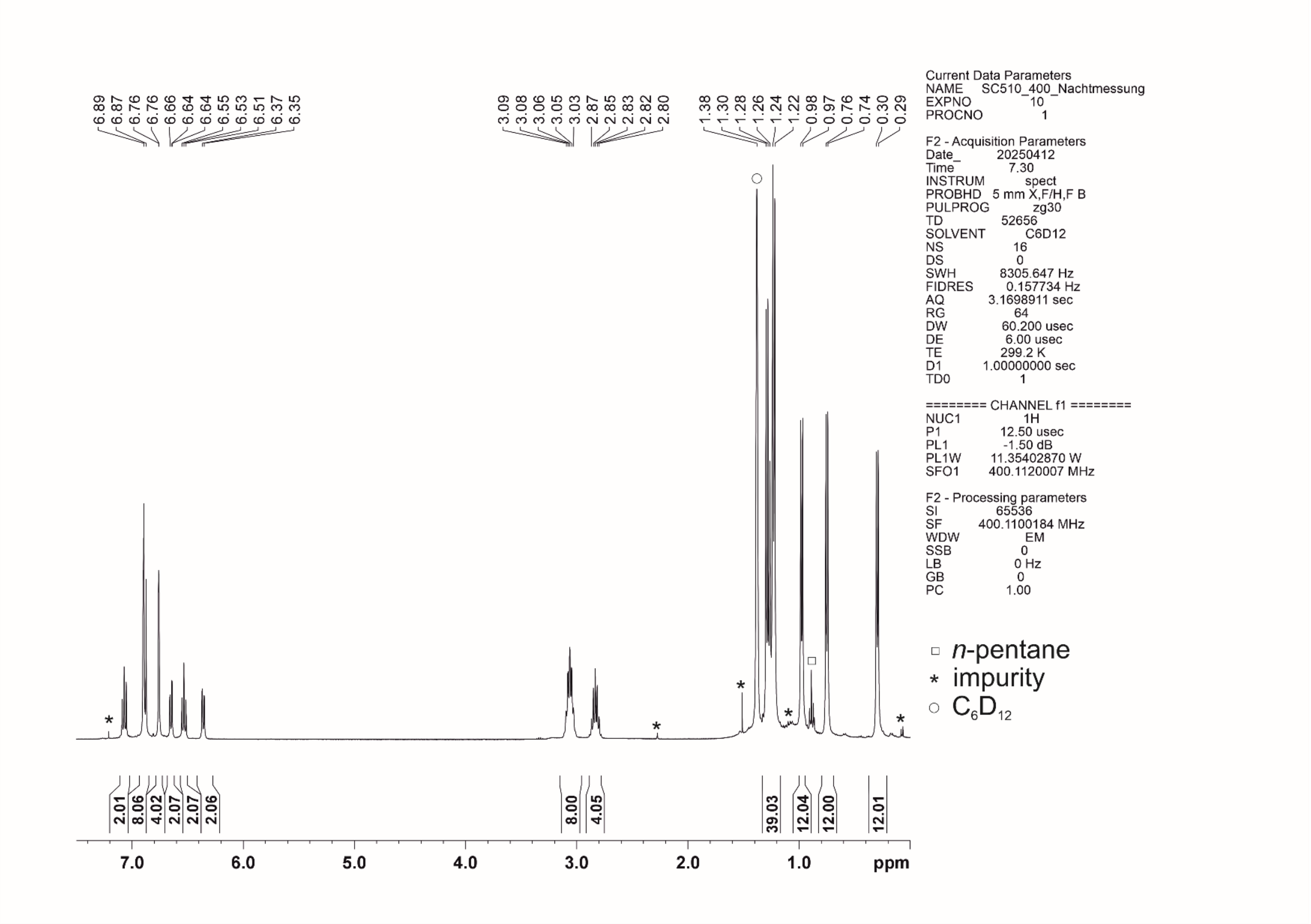

Figure SI7. ^{1}H NMR of compound K_2[**1a**].

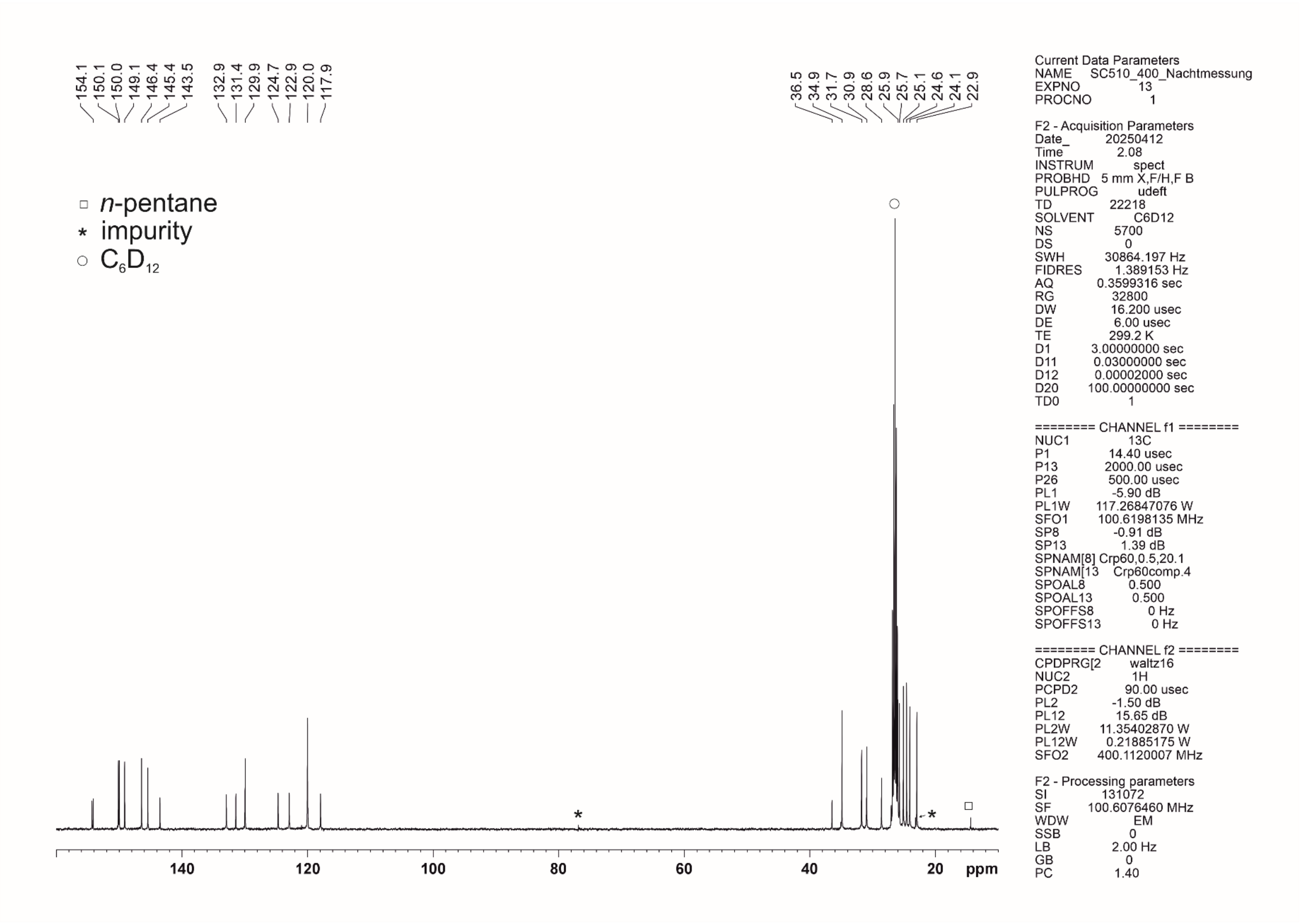

Figure SI8. $^{13}C\{^{1}H\}$ NMR of compound K_2[**1a**].

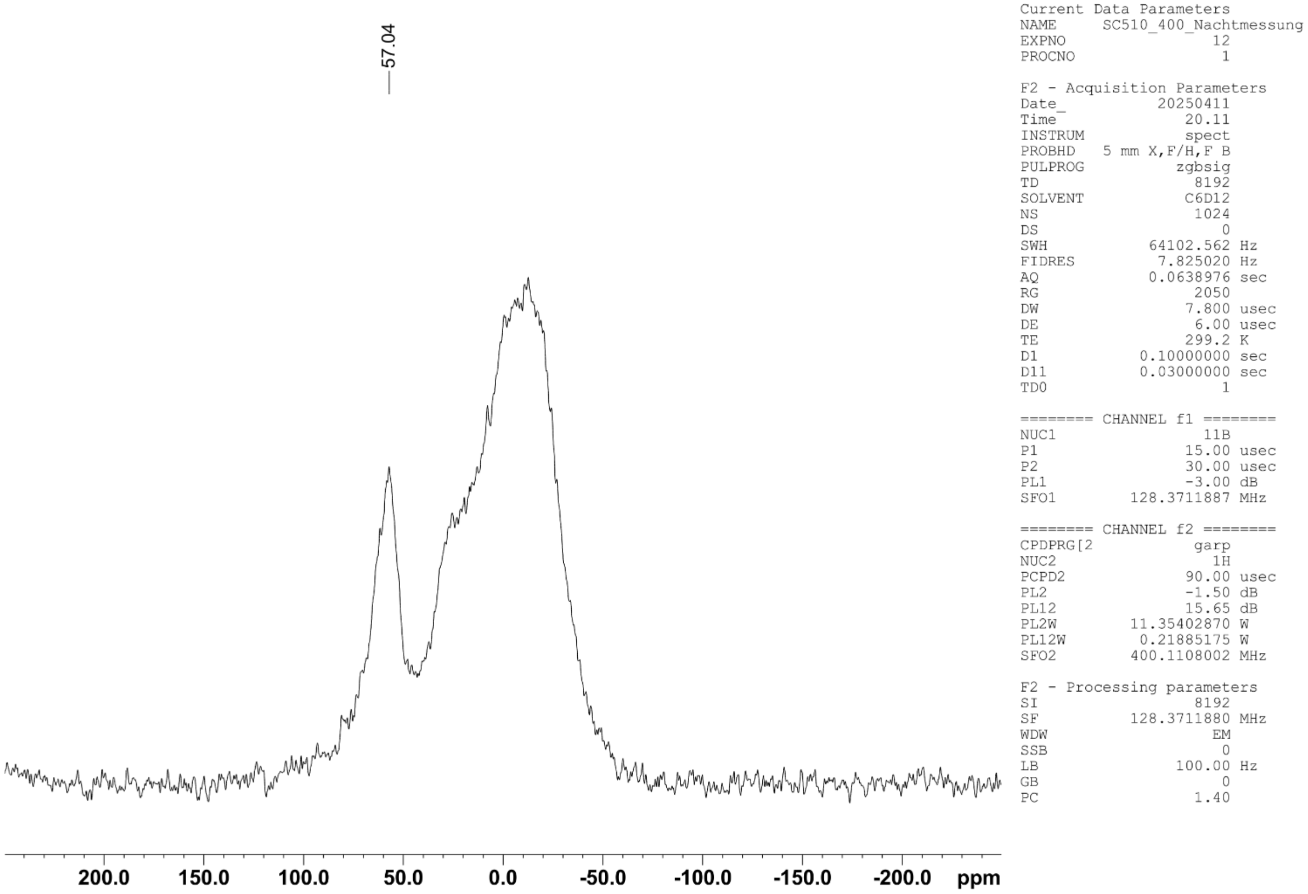

Figure SI9. $^{11}B\{^{1}H\}$ NMR of compound K_2[**1a**].

NMR spectra of compound K_2[**1b**].

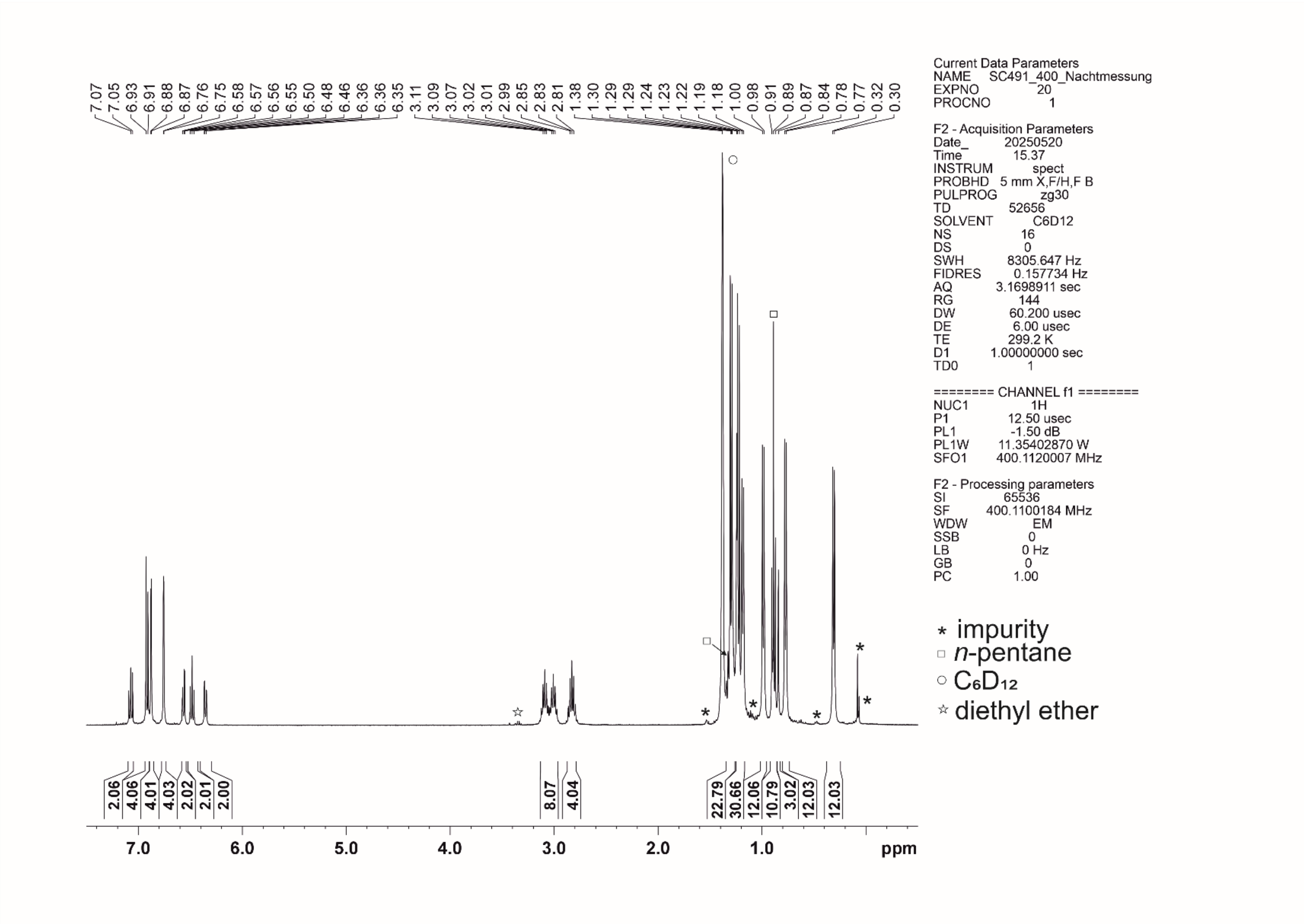

Figure SI10. ^{1}H NMR of compound K_2[**1b**].

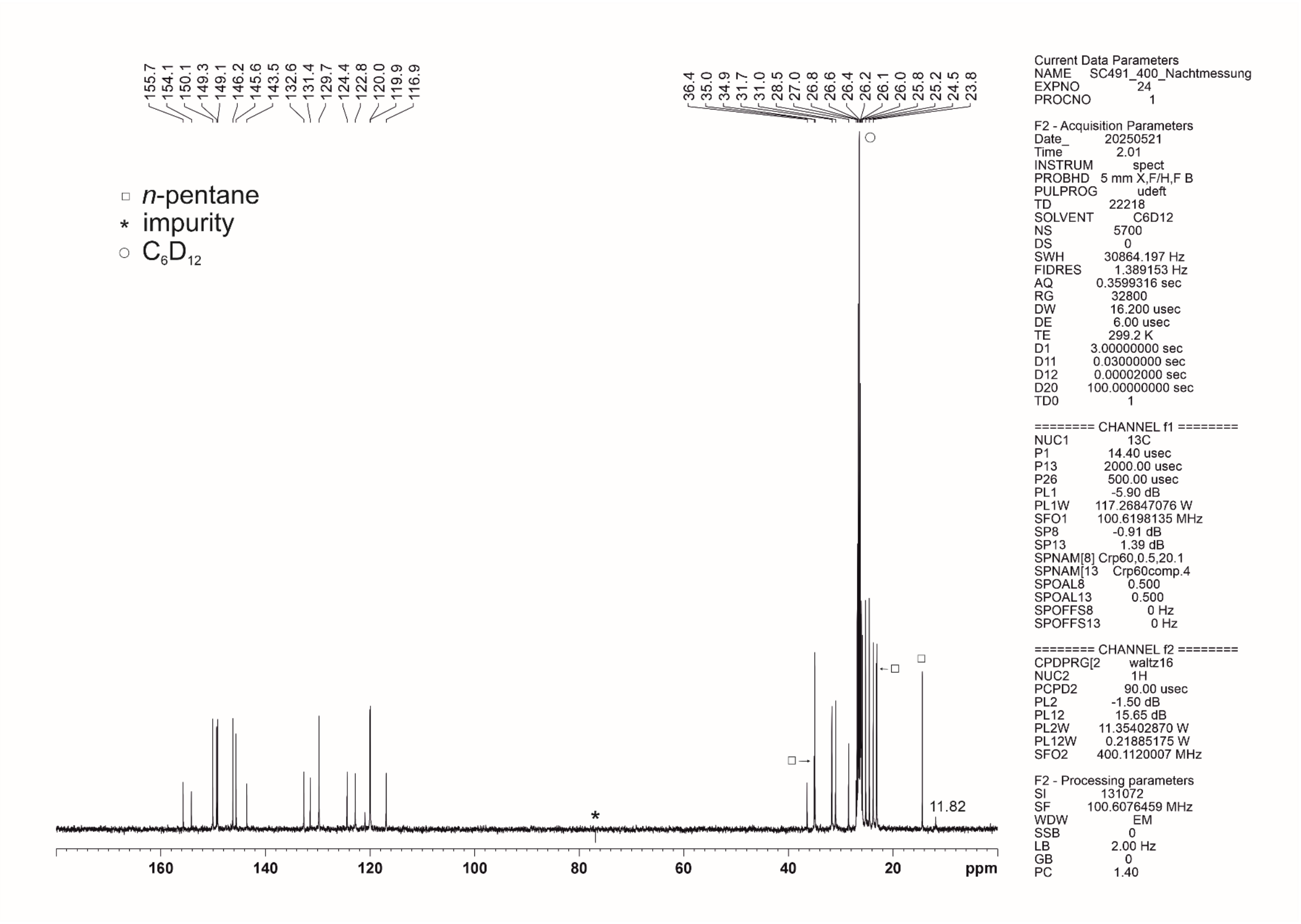

Figure SI11. $^{13}C\{^{1}H\}$ NMR of compound K_2[**1b**].

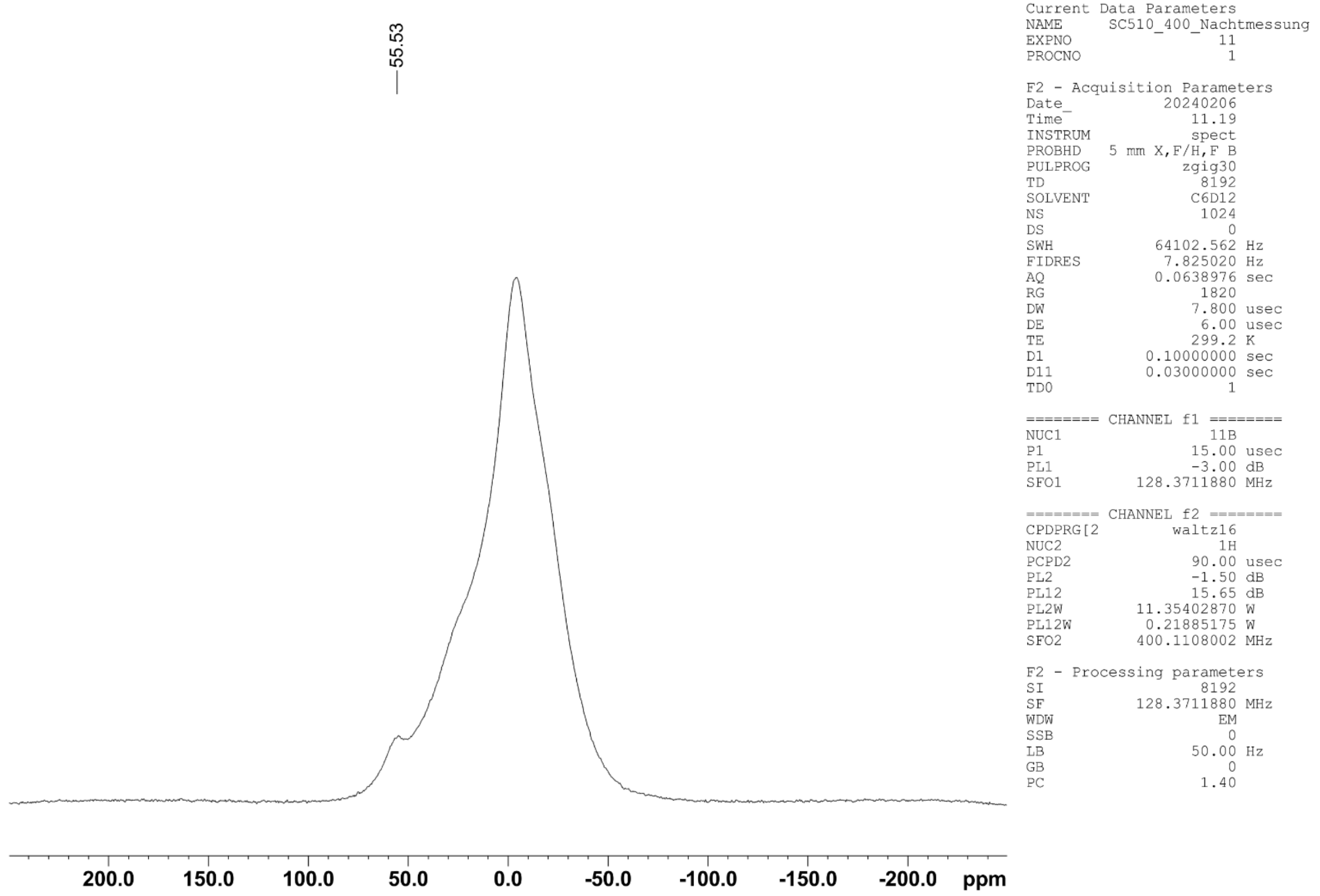

Figure SI12. $^{11}B\{^{1}H\}$ NMR of compound K_2[**1b**].

Comparison of the ^{11}B NMR chemical shifts of **1a** and **1a^{2-}**.

The observation that the ^{11}B NMR chemical shift of the Ge-B-Ge heteroallyl dianion **1a^{2-}**, δ(^{11}B) 57.0 ppm, features a boron atom less shielded than the neutral parent compound **1a**, 42.4 ppm, is surprising on first sight. However, quantum mechanical calculations of magnetic shieldings (see NMR shielding tensor computations) reproduce this trend and provide more insights (Table SI2, Fig. SI13). The results of the calculated ^{11}B magnetic shieldings illustrated in Fig. SI13 (top axis) have been converted to ^{11}B chemical shifts (bottom axis) via the absolute magnetic shielding scale established by Jackowski et al. using σ(^{11}B, BF_3OEt_2) = 110.9 ppm.[11] In both compounds, **1a** and **1a^{2-}**, the magnetic shielding tensor is oriented in the same way (Fig. SI14): the direction of least shielding, σ_{11}, points along the B-Cl bond, the direction of highest shielding, σ_{33}, is perpendicular to the plane of the heteroallyl skeleton, and the direction of intermediate shielding, σ_{22}, completes the axes system, being in-plane and perpendicular to B-Cl. On going from **1a** to **1a^{2-}**, σ_{22} and σ_{33} shift in such a manner that the changes cancel each other, hence the difference in the isotropic shieldings σ_{iso} arises from a significant change in σ_{11}, causing a deshielding contribution in the direction of the B-Cl bond. Contributions to paramagnetic shielding (i.e., deshielding) are caused by magnetic dipole allowed mixing of ground state (occupied) with excited states (empty orbitals)[12, 13] of appropriate symmetry.[14, 15] Because of the size of the molecules **1a**, **1a**$^{2-}$, there are hundreds of MOs (Fig. SI16) that are crucial for the quantitative description of the systems but are lacking in simplicity. Similar to the way boranes helped researchers to deduce the likely structures of isoelectronic hypercarbon systems,[16] we shall reduce complexity in order to qualitatively rationalize the deshielding effect on ^{11}B by examining the prototypal allyl cation in point group C_{2v} and its MO diagram given in Fig. SI15. The picture has been produced using the extended Hückel MO program CACAO,[17] with results similar to published illustrations.[18] According to group theory, mixing of orbitals that causes deshielding along the z axis (central C-H in allyl, B-Cl in **1a**, **1a^{2-}**) must yield the irreducible representation A_2 (containing R_z) as direct product, i.e. the products $A_2 \otimes A_1$ and $B_2 \otimes B_1$. In the allyl cation, the MO $1b_1$ is the HOMO and $1a_2$ the LUMO. Mixing of these orbitals results in B_2, which is effective in x direction (the most shielded direction), therefore must be not important. In contrast, in the allyl anion $1a_2$ is the HOMO and can interact with the low lying empty $5a_1$ orbital. Interestingly, in the carbon system the central carbon in related allyl cations and anions have quite similar chemical shifts, the differences between the two systems manifested in the terminal carbon atoms: 1,3-dimethylallyl MeHC-C$^+$H-CHMe ($C^{1,3}$: 232.2, C^2: 148.3 ppm)[19] and 1-methylallyl MeHC-C$^+$H-CH_2 (C^1: 255.1, C^2: 149.8, C^3: 201.5 ppm)[20] versus allyl lithium ($C^{1,3}$: 51.2, C^2: 147.2 ppm)[21] or allyl potassium ($C^{1,3}$: 52.8, C^2: 144.0 ppm).[22]

Table SI2. ^{11}B NMR magnetic shielding tensors[a)] of **1a** and **1a^{2-}**, (see NMR shielding tensor computations)

compd.	σ_{iso} / ppm	σ_{11} / ppm	σ_{22} / ppm	σ_{33} / ppm
**1a**	66.3	41.4	62.4	95.0
**1a^{2-}**	45.4	-23.4	73.5	85.9

[a)] $\sigma_{11} \geq \sigma_{22} \geq \sigma_{33}$; $\sigma_{iso} = (\sigma_{11} + \sigma_{22} + \sigma_{33})/3$

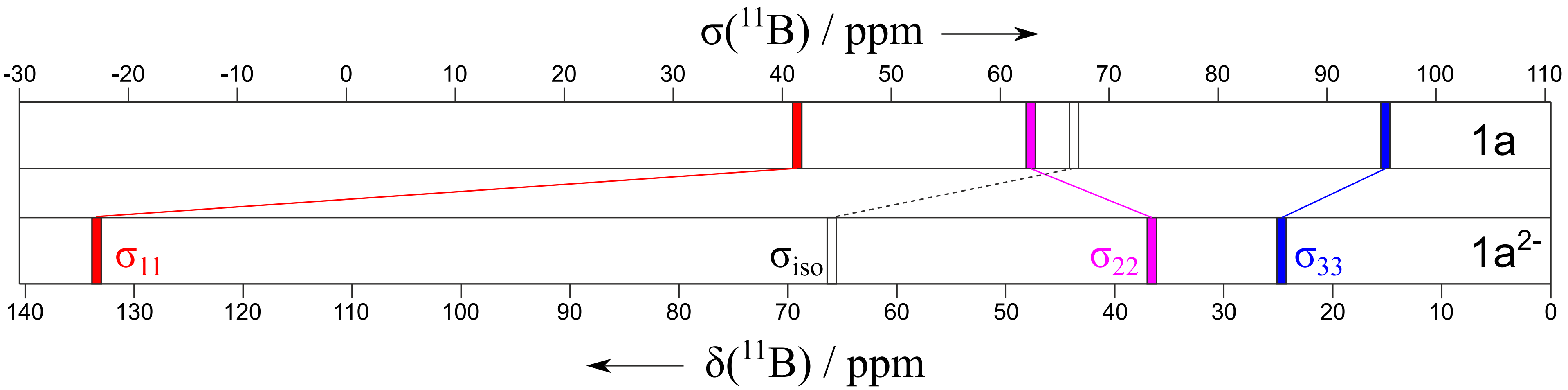

Fig. SI13. Changes in ^{11}B NMR magnetic shielding tensor obtained from quantum mechanical calculations (see NMR shielding tensor computations) on **1a** and **1a**$^{2-}$ ($\sigma_{11} \geq \sigma_{22} \geq \sigma_{33}$; $\sigma_{iso} = (\sigma_{11} + \sigma_{22} + \sigma_{33})/3$).

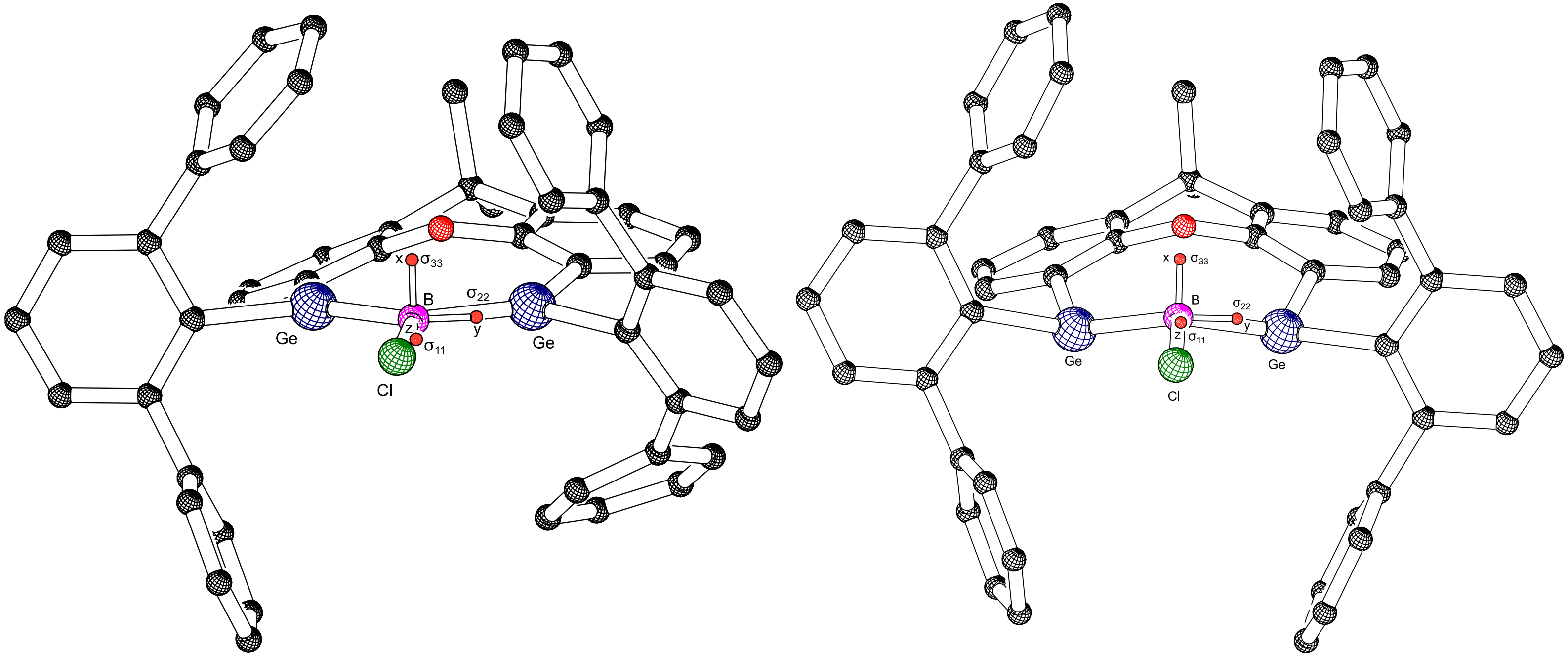

Fig. SI14. Orientations of ^{11}B magnetic shielding tensors obtained from quantum mechanical calculations on **1a** (left) and **1a^{2-}** (right).

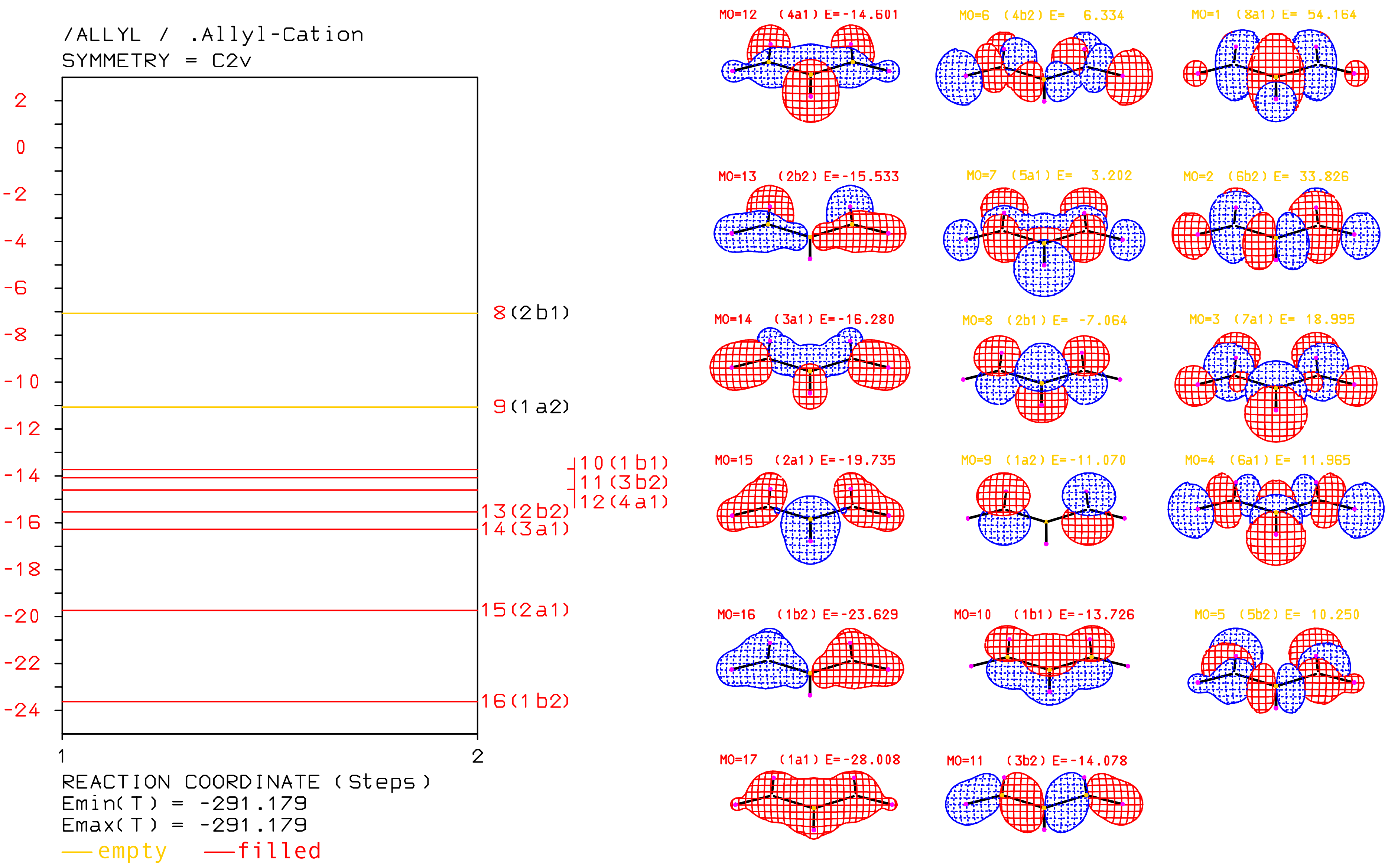

Fig. SI15. EHMO diagram of the allyl cation, produced using CACAO.[17]

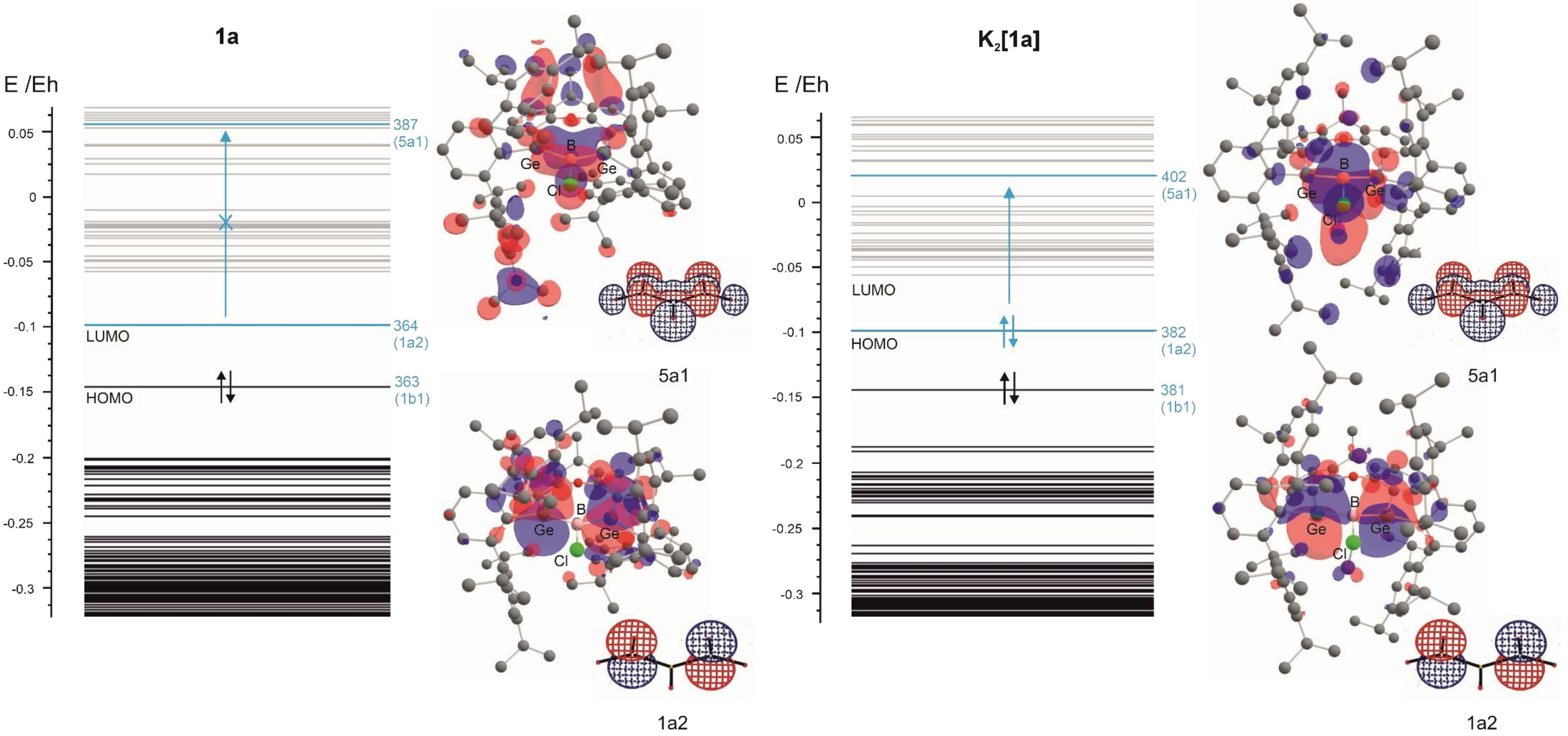

Fig. SI16. Molecular orbitals of **1a** and **1a**$^{2-}$ corresponding to the 1a2 and 5a1 EHMO orbitals of the allyl cation.

UV-Vis spectroscopy

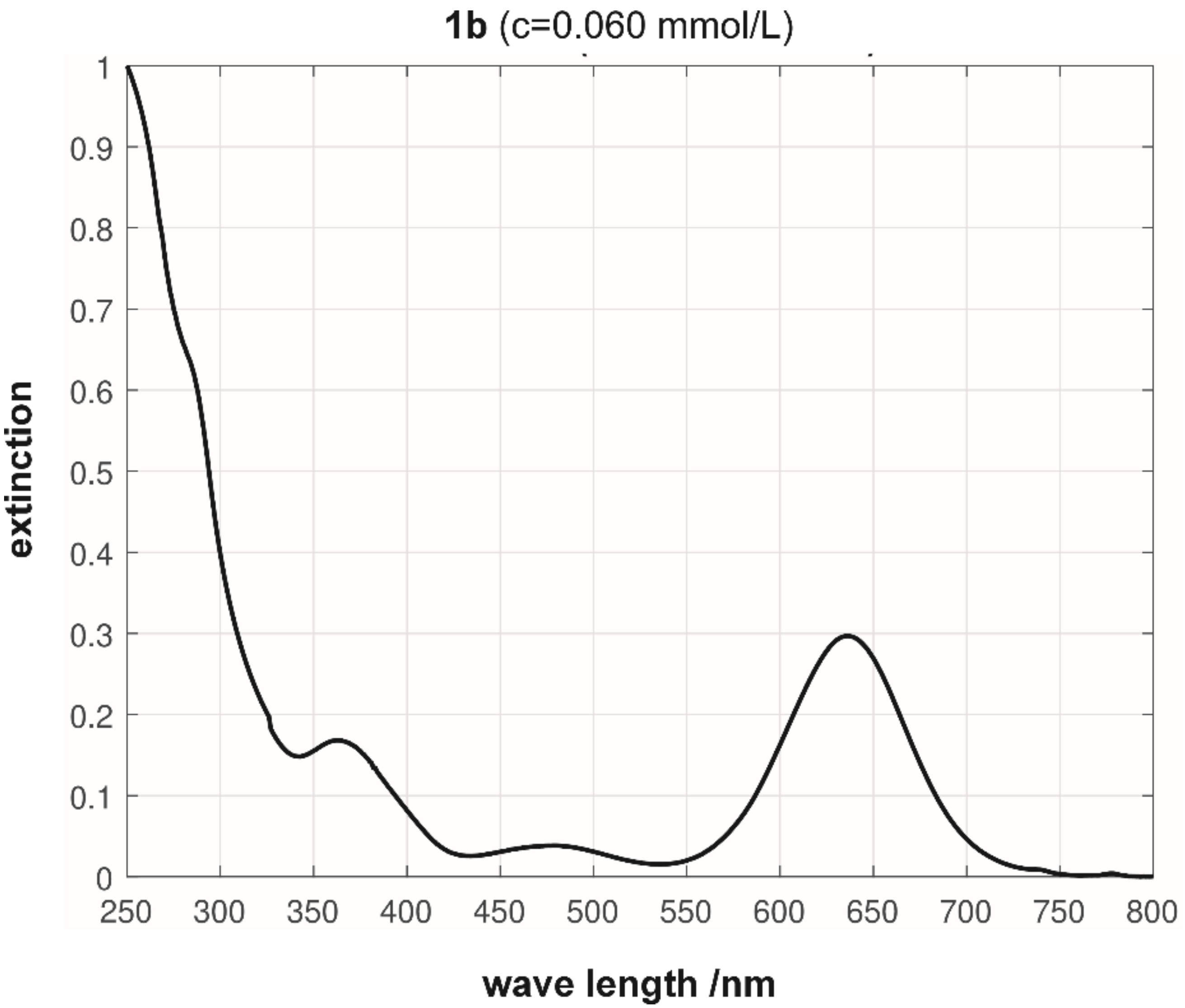

Figure SI17. UV-Vis spectrum of compound **1b**.

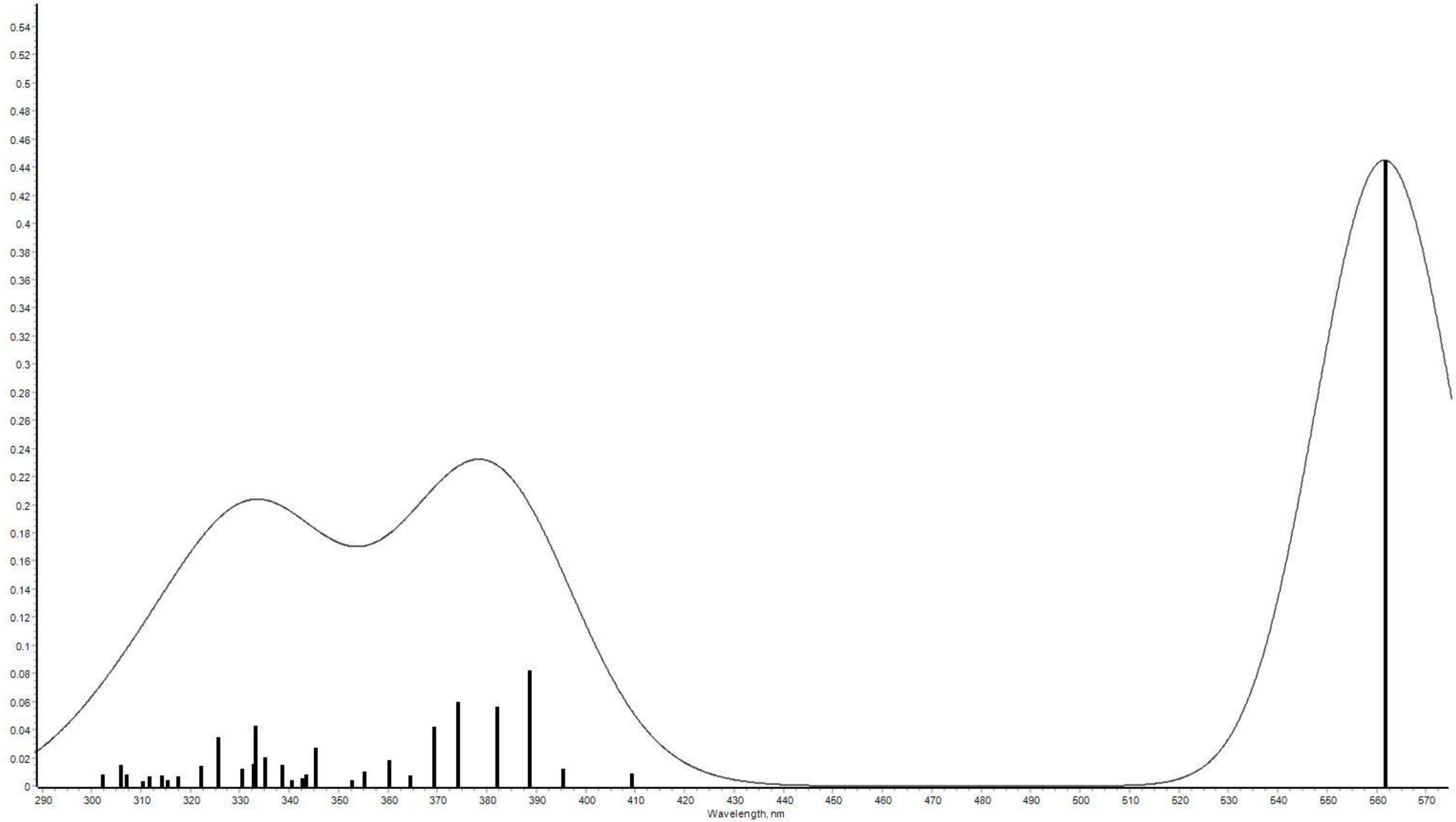

Figure SI18. Absorption spectrum computed at the TD-B3LYP/def2-TZVP level of theory of compound **1b.**

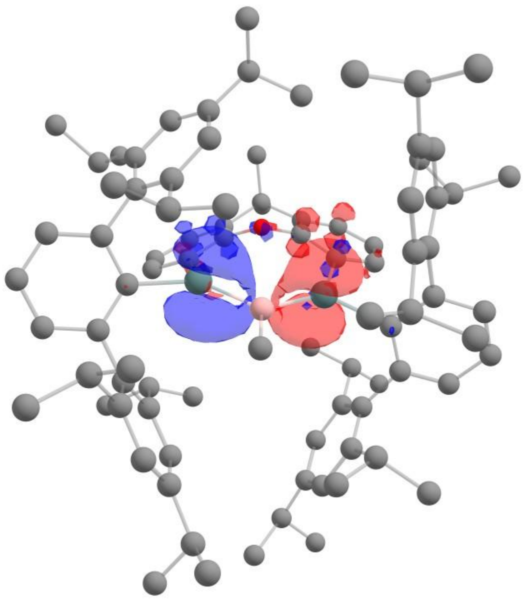

Figure SI19. TD-DFT transition densities of state 1 at the TD-B3LYP/def2-TZVP level of theory of compound **1b.**

Table SI3. UV-Vis transitions (TD-B3LYP/def2TZVP) (out of excited state 1-5, c > 0.3) of **1b**.

State	energy (cm^{-1})	wavelength (nm)	fosc	Relevant Transitions
1	17807.3	561.6	0.45	**HOMO → LUMO** (0.915681; c=-0.9569)
2	24442.7	409.3	0.01	HOMO → LUMO+1 (0.809156; c=0.8995), HOMO → LUMO+2 (0.160202; c=-0.4003)
3	25297.3	395.3	0.01	HOMO → LUMO+3 (0.914342; c=0.9562)
4	25749.9	388.3	0.08	HOMO → LUMO+2 (0.667804; c=0.8172), HOMO → LUMO+4 (0.189342; c=-0.4351), HOMO → LUMO+5 (0.189342; c= -0.4351)
5	26184.9	381.9	0.06	HOMO−1 → LUMO (0.895400; c=-0.9463)

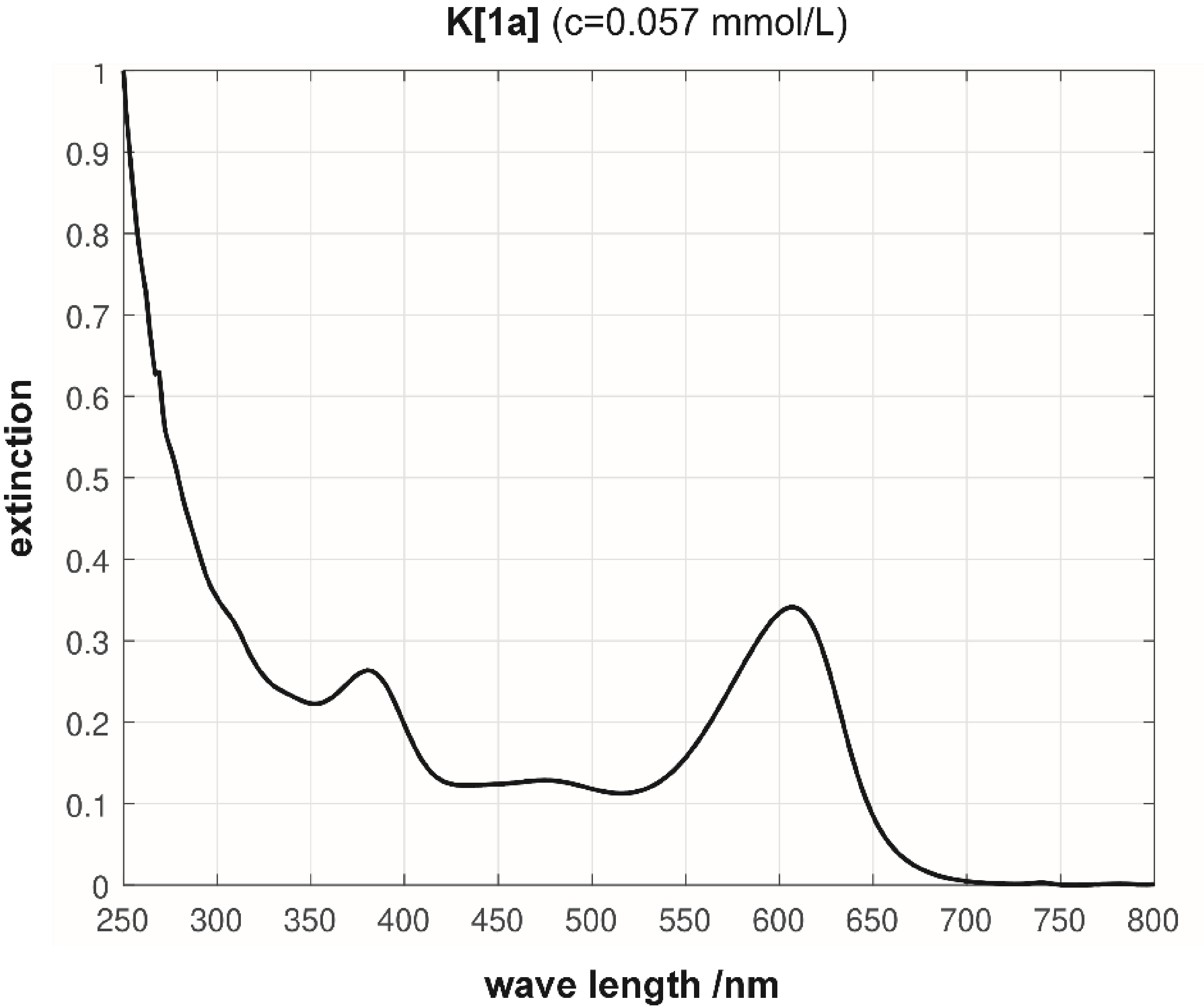

Figure SI20. UV-Vis spectrum of compound **K[1a]**.

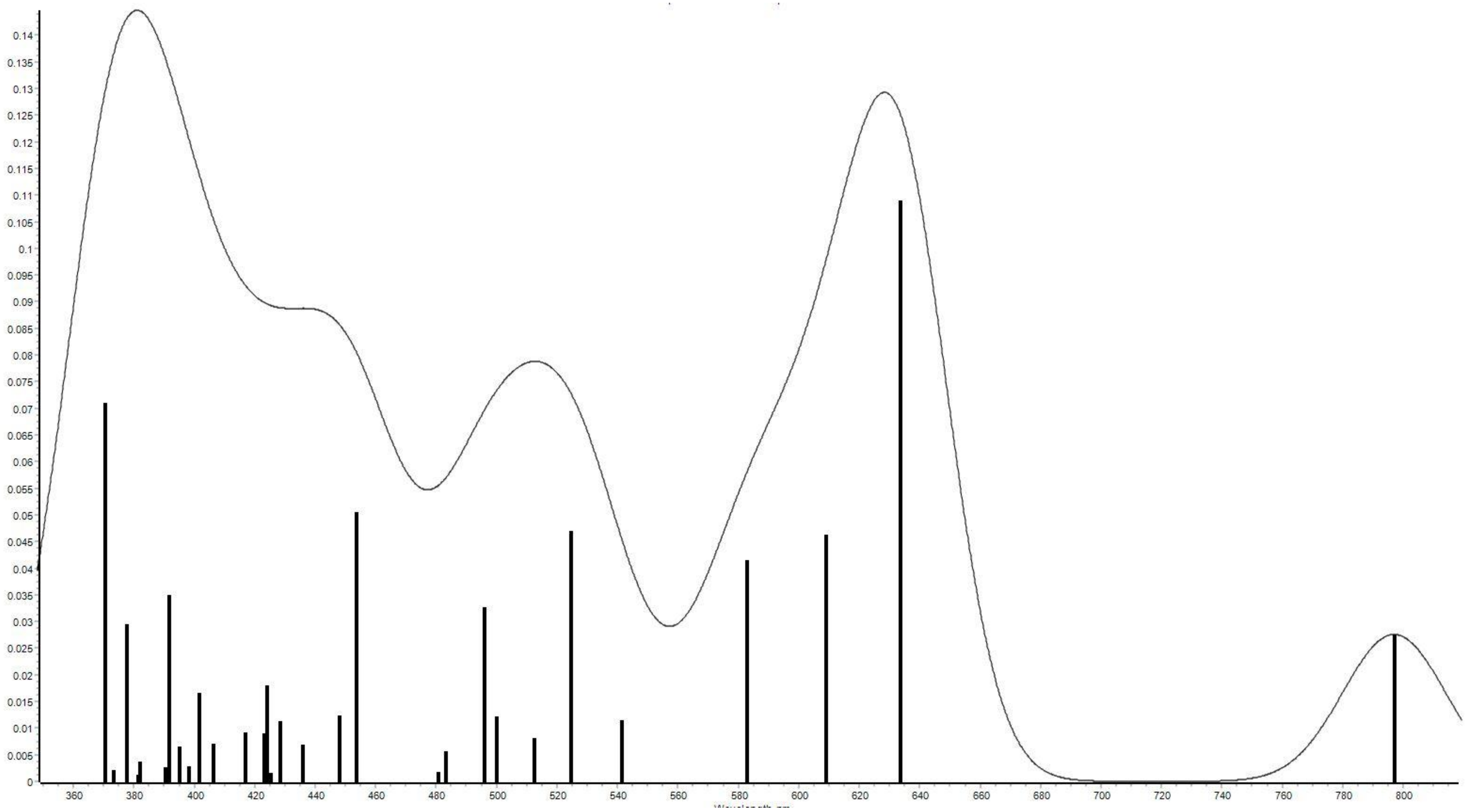

Figure SI21. Absorption spectrum computed at the TD-B3LYP/def2-TZVP level of theory of compound **K[1a]**.

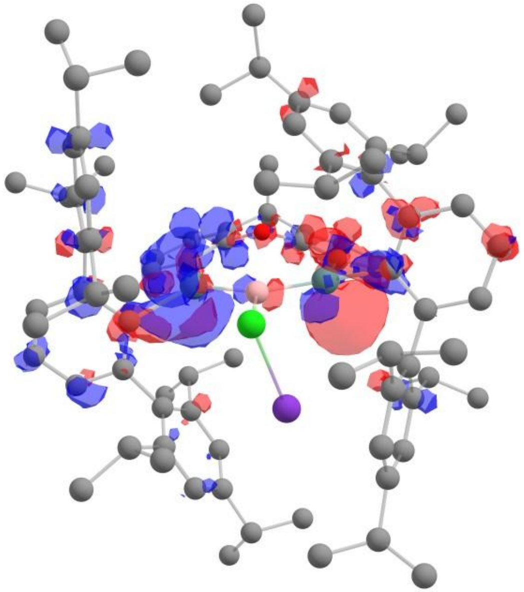

Figure SI22. TD-DFT transition densities of state 2 at the TD-B3LYP/def2-TZVP level of theory of compound **K[1a].**

Table SI4. UV-Vis transitions (TD-B3LYP/def2TZVP) (out of excited state 1-5, c > 0.3) of **K[1a]**.7

State	energy (cm^{-1})	wavelength (nm)	fosc	Relevant Transitions
2	15791.1	633.5	0.11	**α-HOMO → α-LUMO** (0.884719; c=0.9406)
3	16428.0	608.6	0.05	α-HOMO → α-LUMO+1 (0.908662; c=-0.9532)
4	17166.1	582.5	0.04	α-HOMO → α-LUMO+2 (0.880230; c=-0.9382)
5	18479.3	541.1	0.01	α-HOMO → α-LUMO+3 (0.897150; c=-0.9472)

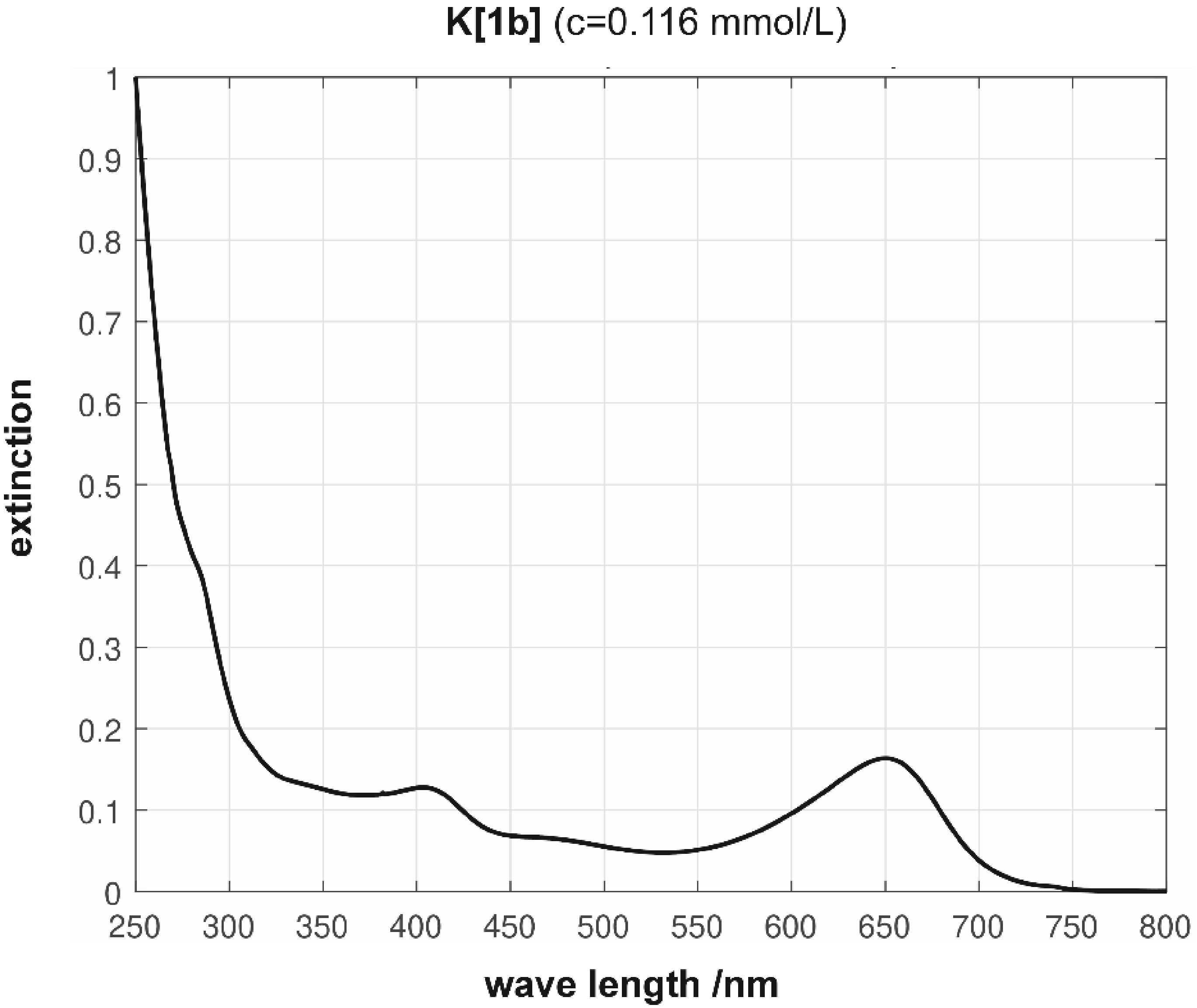

Figure SI23. UV-Vis spectrum of compound **K[1b]**.

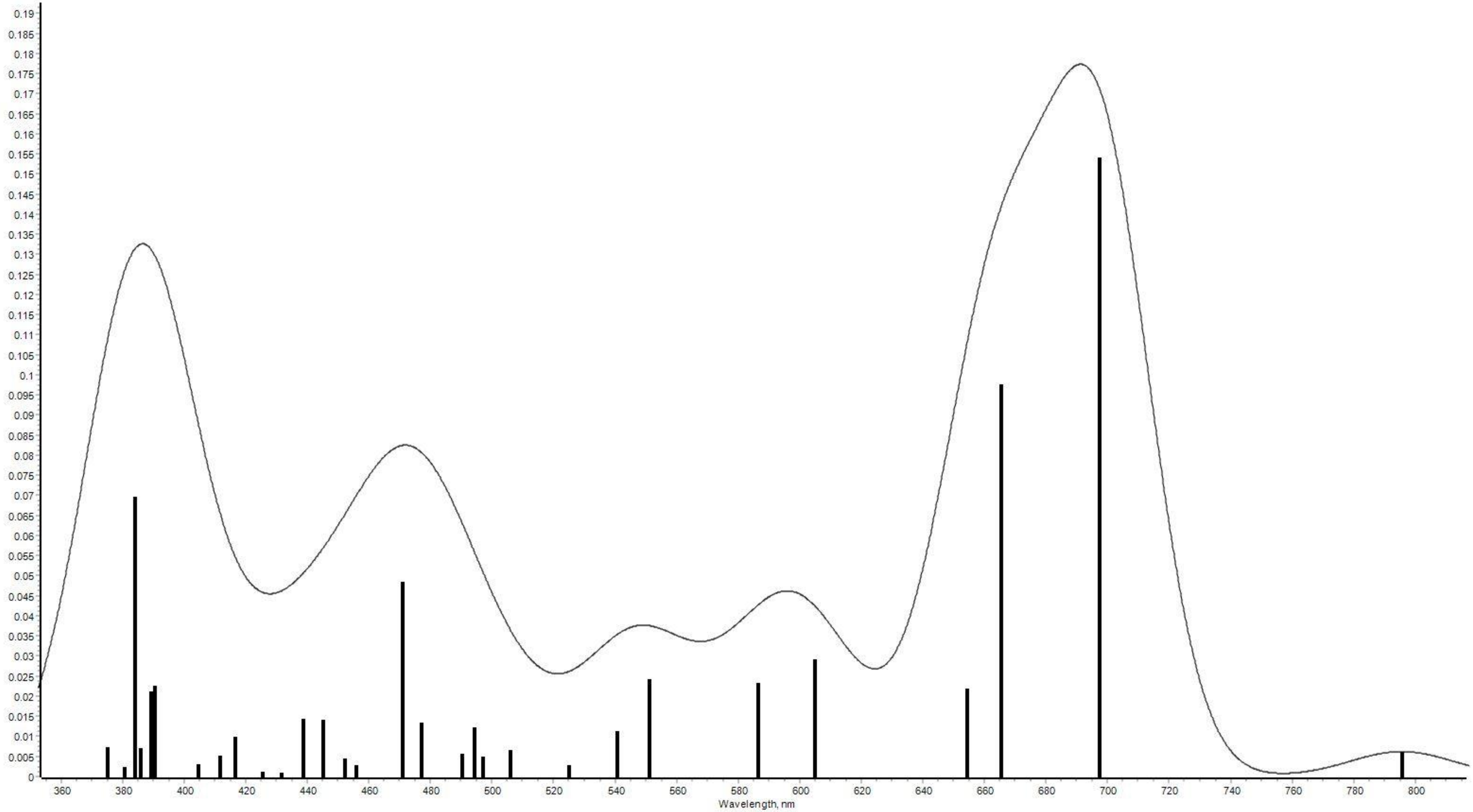

Figure SI24. Absorption spectrum computed at the TD-B3LYP/def2-TZVP level of theory of compound **K[1b]**.

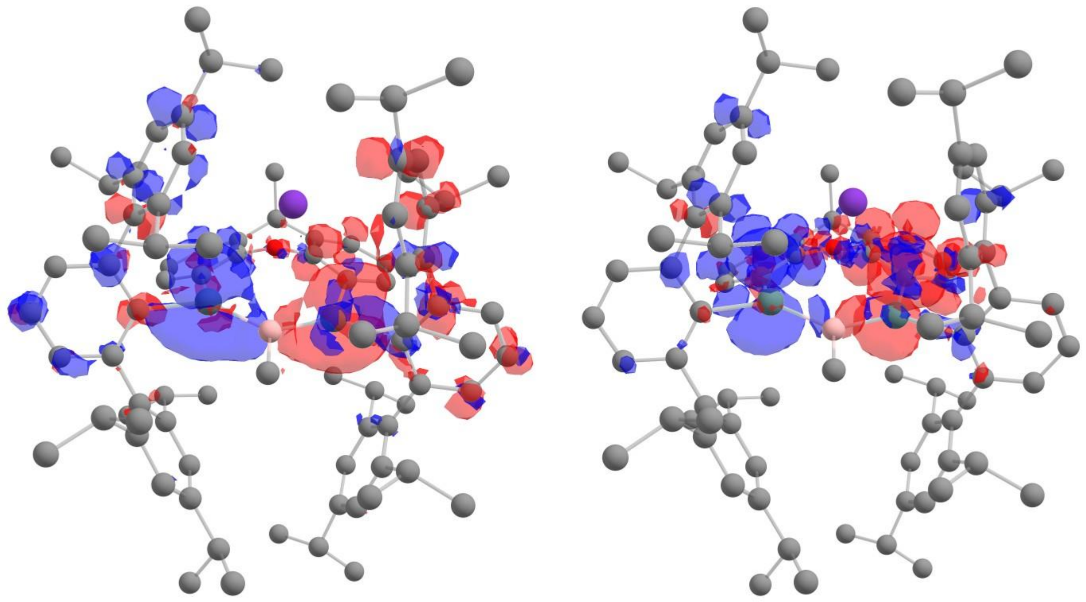

Figure SI25. TD-DFT transition densities of state 2 (left) and 3 (right) at the TD-B3LYP/def2-TZVP level of theory of compound **K[1b].**

Table SI5**.** UV-Vis transitions (TD-B3LYP/def2TZVP) (out of excited state 1-5, c > 0.3) of **K[1b]**.

State	energy (cm^{-1})	wavelength (nm)	fosc	Relevant Transitions
2	14343.0	697.2	0.15	**α-HOMO → α-LUMO** (0.664558; c=-0.8152),<br>β-HOMO → β-LUMO (0.176698; c= 0.4204)<br>α-HOMO → α-LUMO+1 (0.091045; c= -0.3017)
3	15029.8	665.4	0.10	**α-HOMO → α-LUMO+1** (0.753214; c=0.8679)<br>β-HOMO → β-LUMO (0.138376; c= 0.3720)
4	15289.6	654.1	0.02	α-HOMO → α-LUMO+2 (0.875497; c=0.9357)
5	16540.9	604.5	0.03	α-HOMO → α-LUMO+3 (0.927893; c=-0.9633)

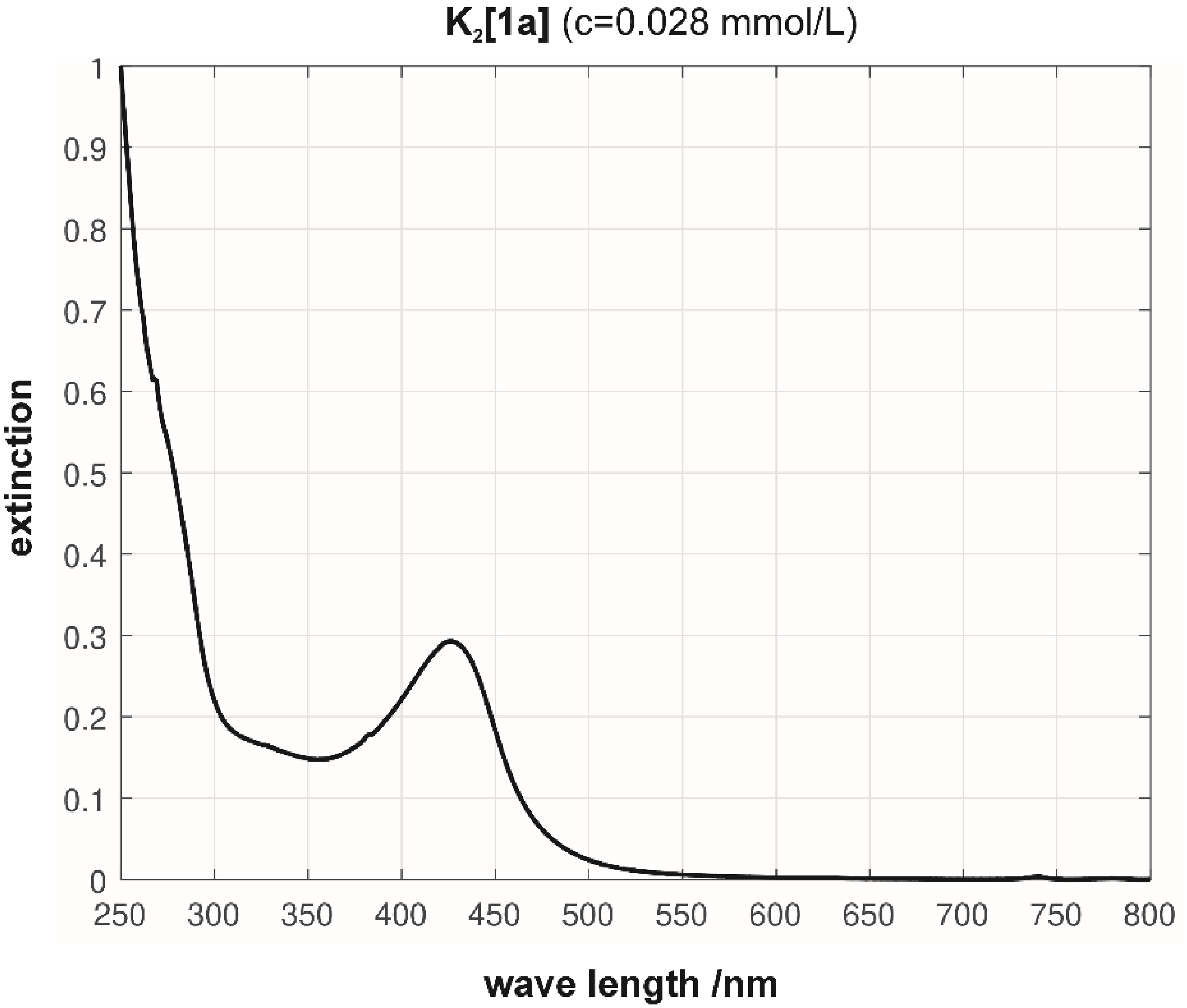

Figure SI26. UV-Vis spectrum of compound **K_2[1a]**.

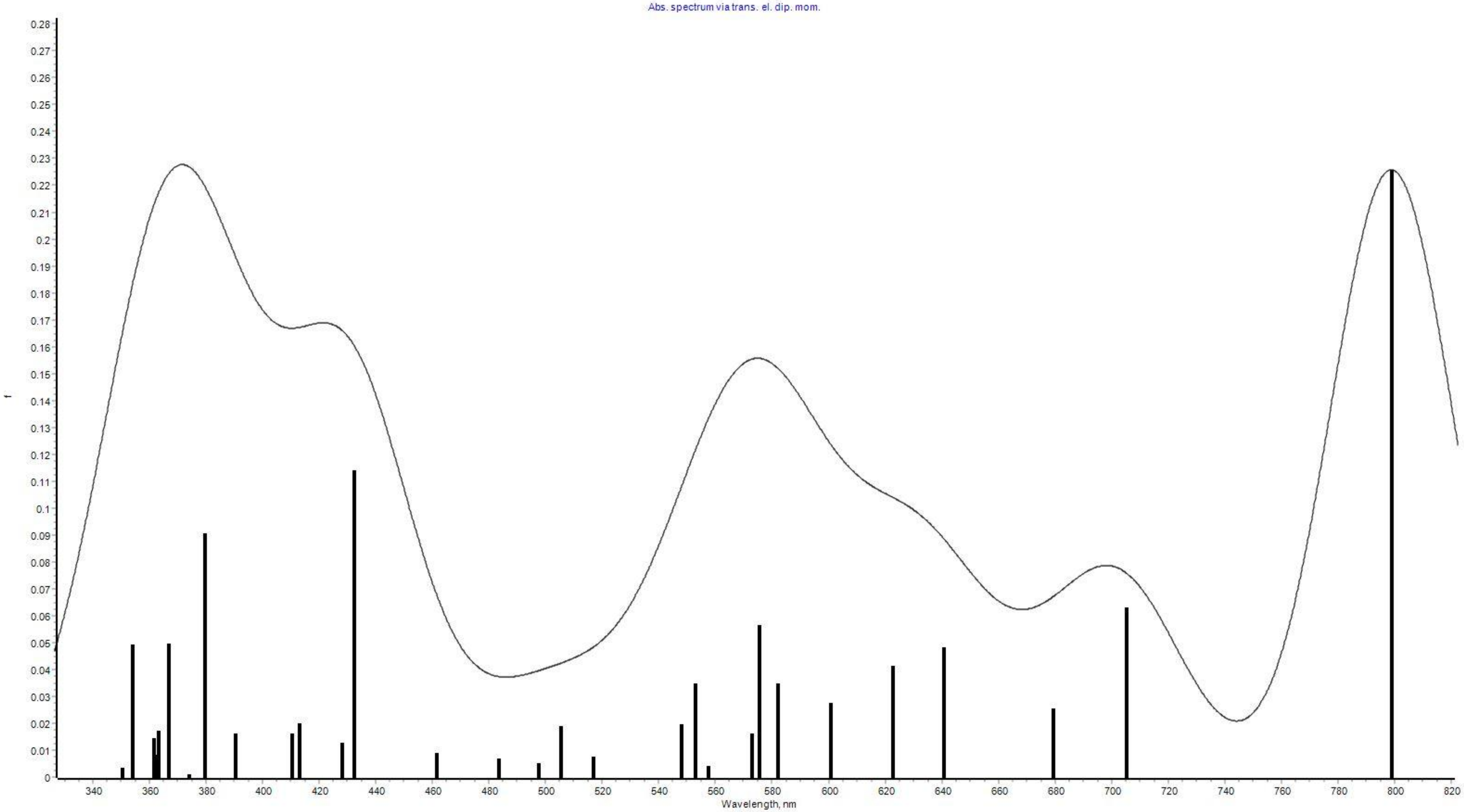

Figure SI27. Absorption spectrum computed at the TD-B3LYP/def2-TZVP level of theory of compound **K_2[1a]**.

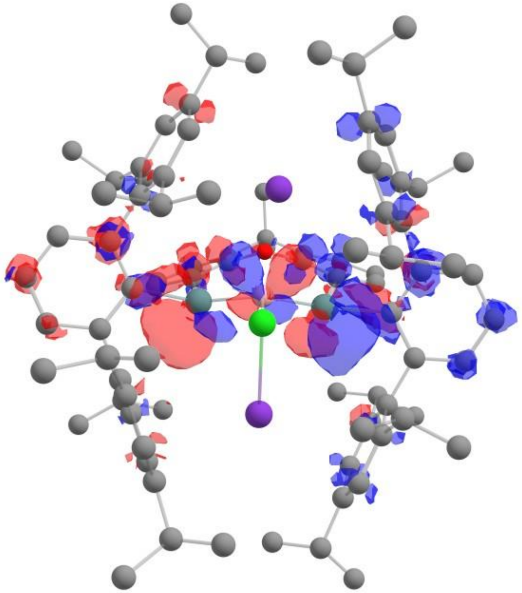

Figure SI28. TD-DFT transition densities of state 2 at the TD-B3LYP/def2-TZVP level of theory of compound **K_2[1a]**.

Table SI6. UV-Vis transitions (TD-B3LYP/def2TZVP) (out of excited state 1-5, c > 0.3) of **K_2[1a]**.

State	energy (cm^{-1})	wavelength (nm)	fosc	Relevant Transitions
2	14186.9	704.9	0.06	**HOMO → LUMO+1** (0.941813; c=-0.9705)
3	14725.7	679.3	0.03	HOMO → LUMO+2 (0.930408; c=0.9646)
4	15609.3	640.6	0.05	HOMO → LUMO+3 (0.922726; c=0.9606)
5	16068.8	622.2	0.04	HOMO → LUMO+4 (0.917306; c=-0.9578)

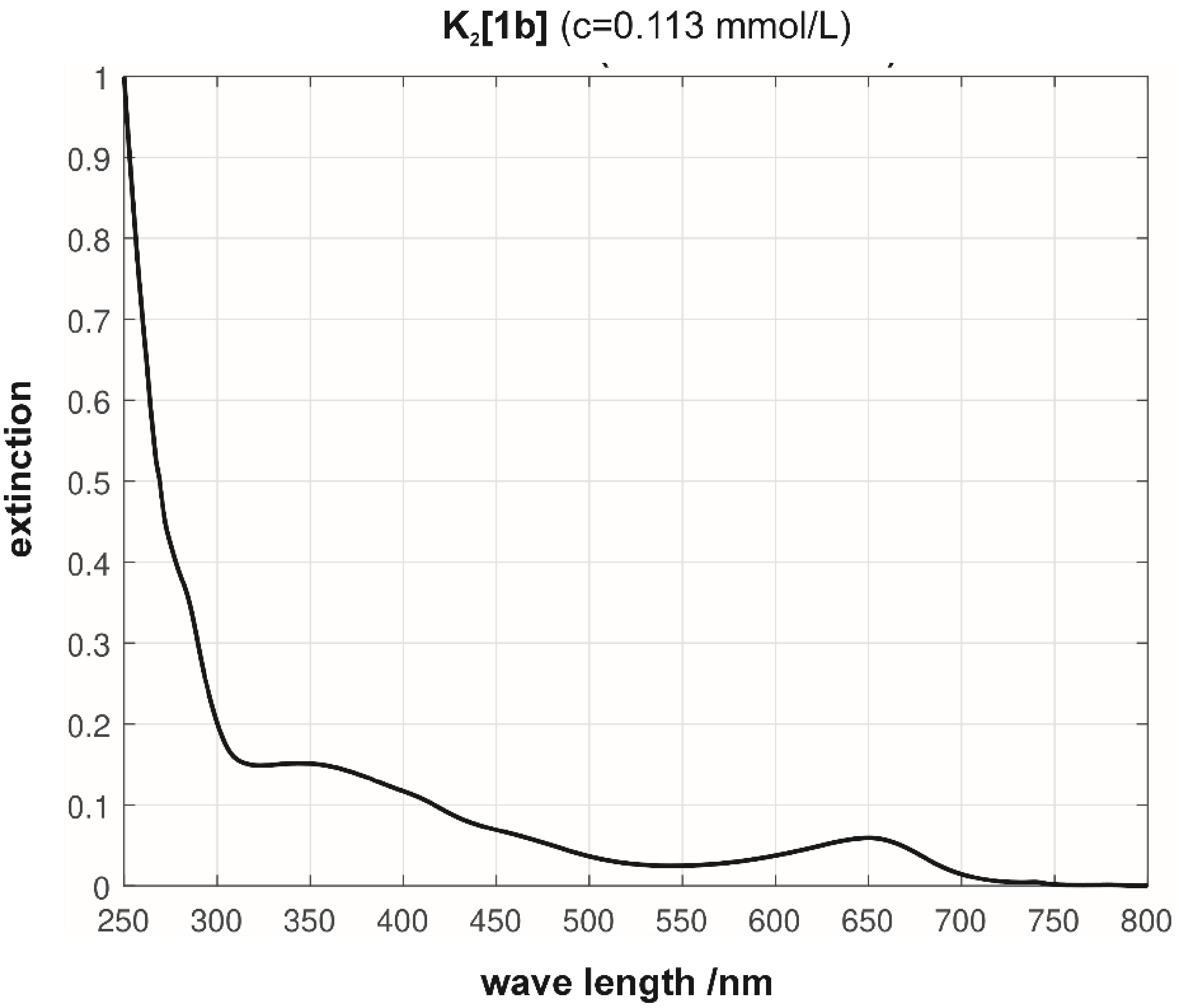

Figure SI29. UV-Vis spectrum of compound **K_2[1b]**.

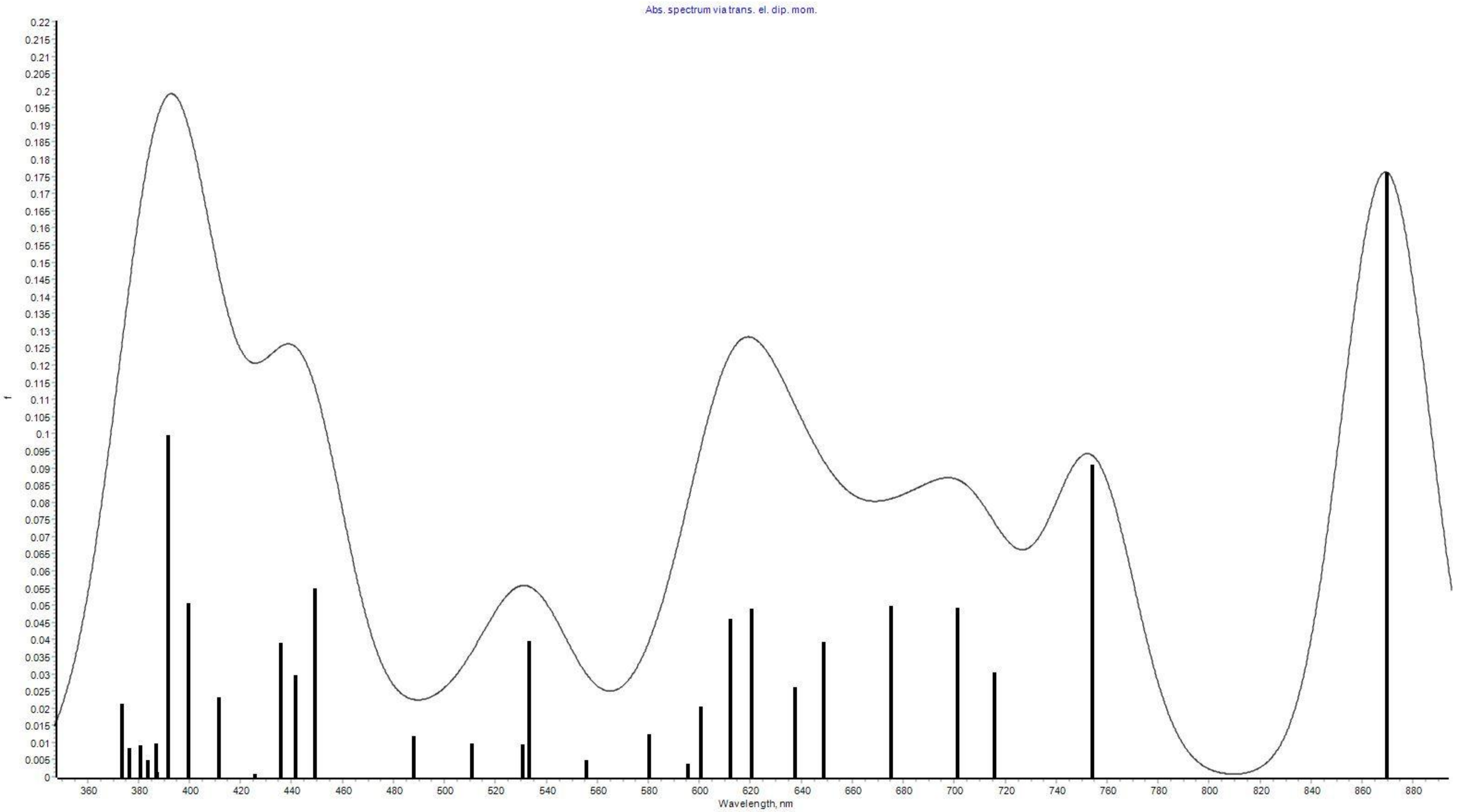

Figure SI30. Absorption spectrum computed at the TD-B3LYP/def2-TZVP level of theory of compound **K_2[1b]**.

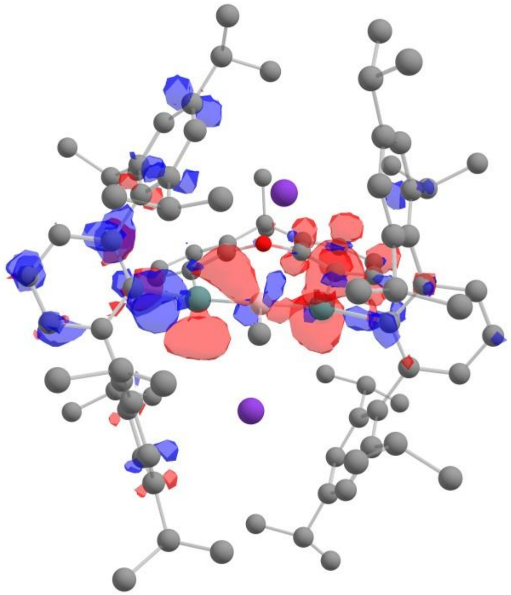

Figure SI31. TD-DFT transition densities of state 2 at the TD-B3LYP/def2-TZVP level of theory of compound **K_2[1b]**.

Table SI7**.** UV-Vis transitions (TD-B3LYP/def2TZVP) (out of excited state 1-5, c > 0.3) of **K_2[1b]**.

State	energy (cm^{-1})	wavelength (nm)	fosc	Relevant Transitions
2	13267.8	753.6	0.09	**HOMO → LUMO+1** (0.945551; c=-0.9724)
3	13975.9	715.5	0.03	HOMO → LUMO+2 (0.894177; c=0.9456)
4	14267.3	701.0	0.05	HOMO → LUMO+3 (0.901529; c=0.9495)
5	14817.6	674.8	0.05	HOMO → LUMO+4 (0.927170; c=0.9629)

EPR spectroscopy

Continuous-wave electron paramagnetic resonance (cw-EPR) spectra were recorded on a *Bruker* EMXmicro spectrometer equipped with a PremiumX X-band microwave bridge (9.3217 GHz). Unless stated otherwise, measurements were performed in solution at room temperature in standard NMR tubes fitted with sealed quartz capillaries (2 mm outer diameter) which are stored protected from light. Spectra were acquired using *Bruker* Xenon software (v1.1b.155). Field calibration was performed using the *Bruker* strong pitch standard (g_{iso} = 2.0028).

Numerical simulations were carried out using the *EasySpin* toolbox (v6.0.2) implemented in *matlab vR2023b*.[23] Simulations were performed using the garlic function, assuming isotropic fast-motion conditions and an electronic ground state of S = 1/2. Fitting parameters included the isotropic g-value (g_{iso}), hyperfine coupling constants (A_{iso}), and linewidths (Lorentzian and Gaussian contributions (full width at half maximum), *lw*).

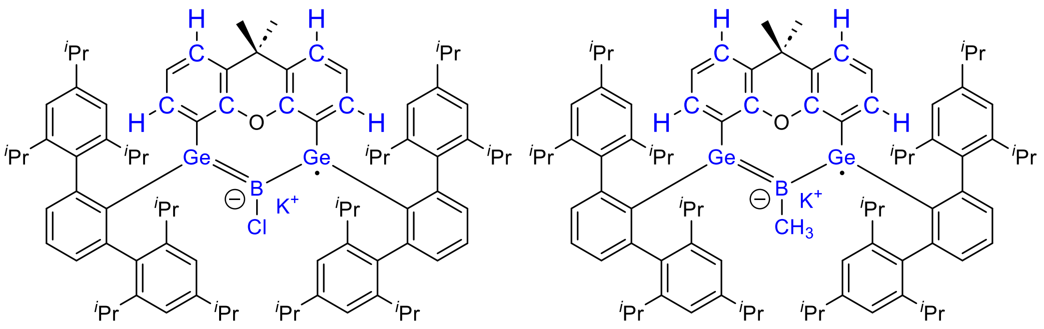

Figure SI32. NMR/EPR active nuclei with localization of significant amount of spin density (blue) of compound K[**1a**$^{\cdot-}$] (left) and K[**1b**$^{\cdot-}$] (right).

All NMR active nuclei (see Figure SI18, blue coloured) with localization of significant amount of spin density (see quantum chemistry) were included in the simulation. The results of the simulations are presented in Table 2 and 3. All nuclei were included in the simulations based on their natural abundance as implemented in *EasySpin*.

Tabel SI8. Fitting parameters (g_{iso} and lw) for EPR spectra of compound K[**1a**$^{\cdot-}$] and K[**1b**$^{\cdot-}$].

	g_{iso}	lw (Gaussian)	lw (Lorentzian)
K[**1a**$^{\cdot-}$]	2.0116	0.1945	0.2097
K[**1b**$^{\cdot-}$]	2.0161	0.4346	0.0939

Table SI9. Fitting parameters (|A_{iso}|) for EPR spectra of compound K[**1a**$^{\cdot-}$] and K[**1b**$^{\cdot-}$].

	A_{iso}(B)	A_{iso}(Ge)	A_{iso}(K)	A_{iso}(Cl)	A_{iso}[H(CH_3)]	A_{iso}[H(Xanth.)]	A_{iso}[C(Xanth.)]
K[**1a**$^{\cdot-}$]	14.07	59.65	1.66	0.28	-	2.22	5.00
K[**1b**$^{\cdot-}$]	14.14	50.69	2.07	-	5.02	2.42	5.13

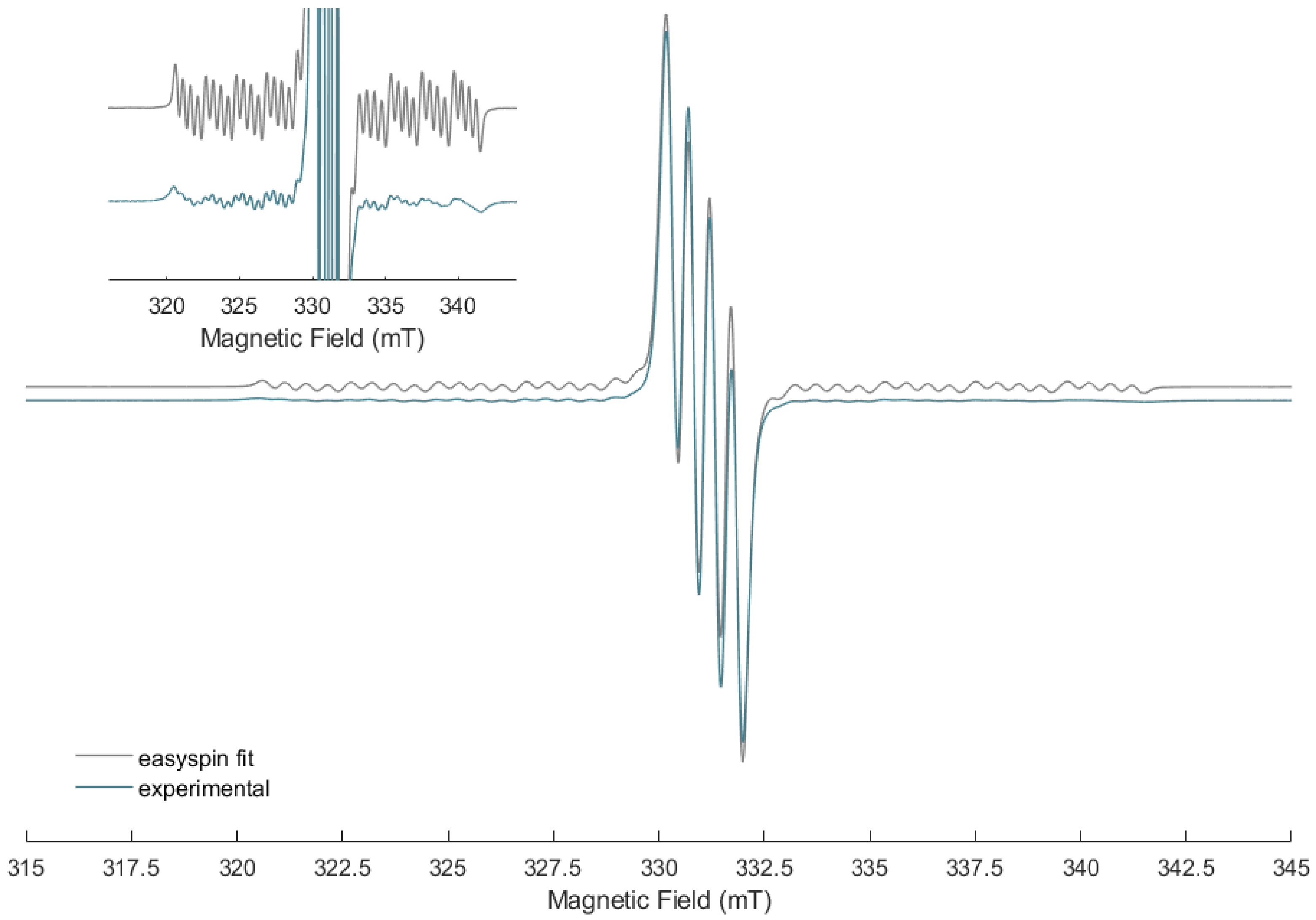

Figure SI33. EPR spectrum of K[**1a**$^{\cdot-}$].

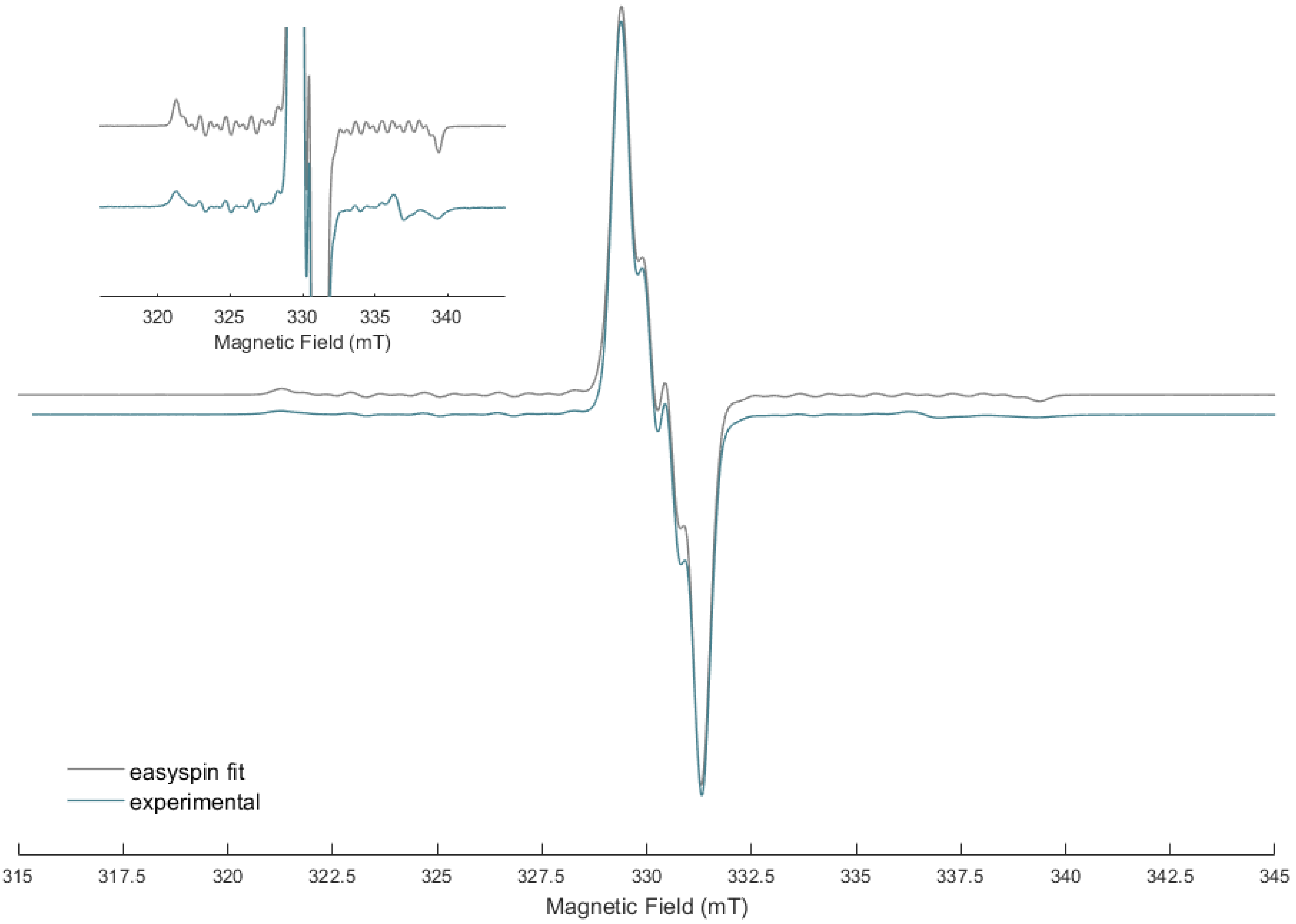

Figure SI34. EPR spectrum of K[**1b**$^{·-}$].

Cyclic voltammetry

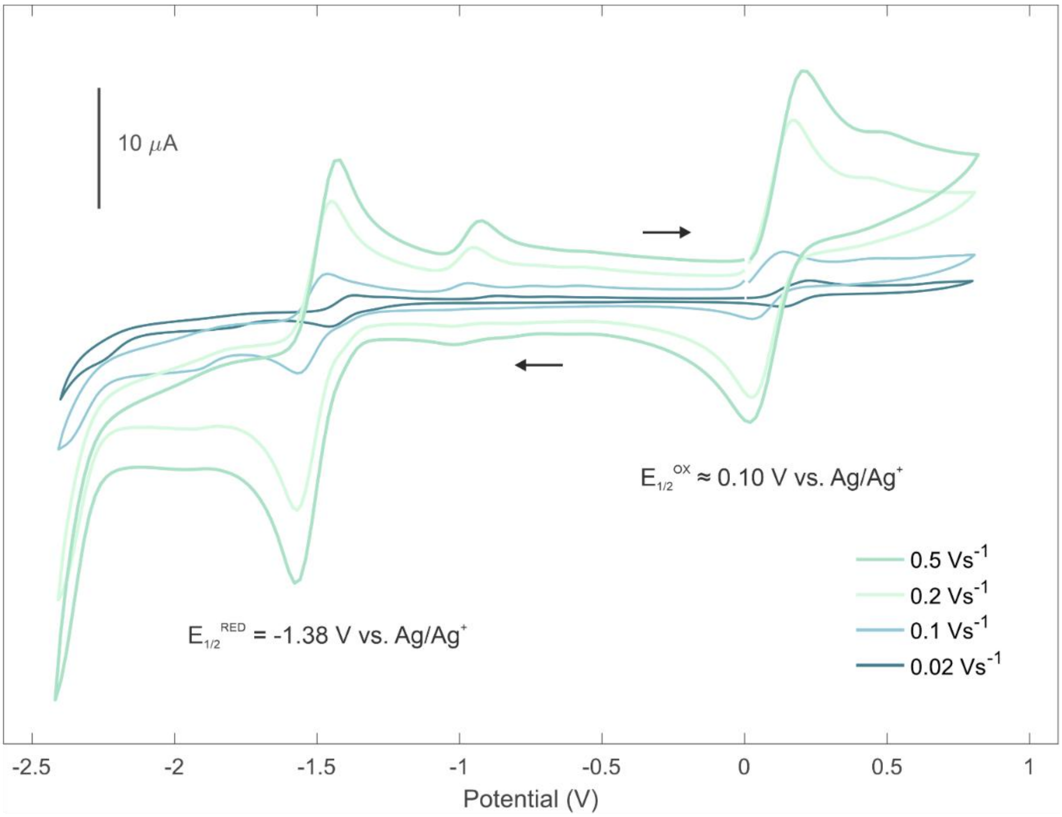

Figure SI35. Cyclic voltammogram of **1a** at different scan rates.

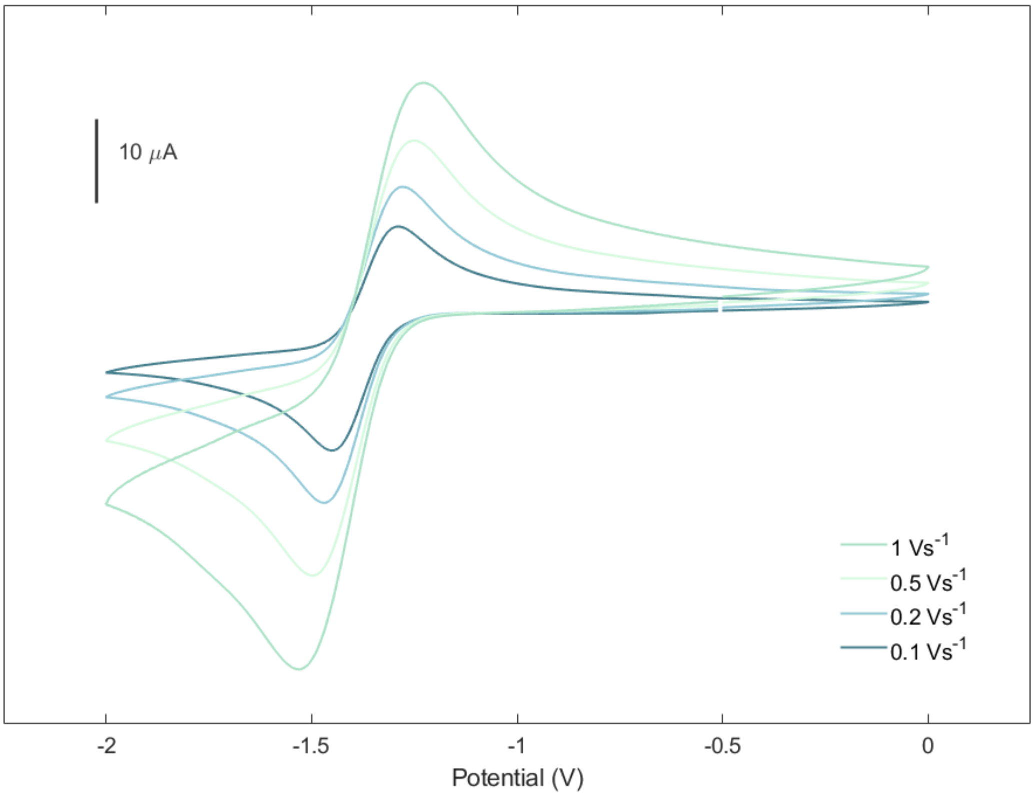

Figure SI36. Cyclic voltammogram of **1a** at different scan rates.

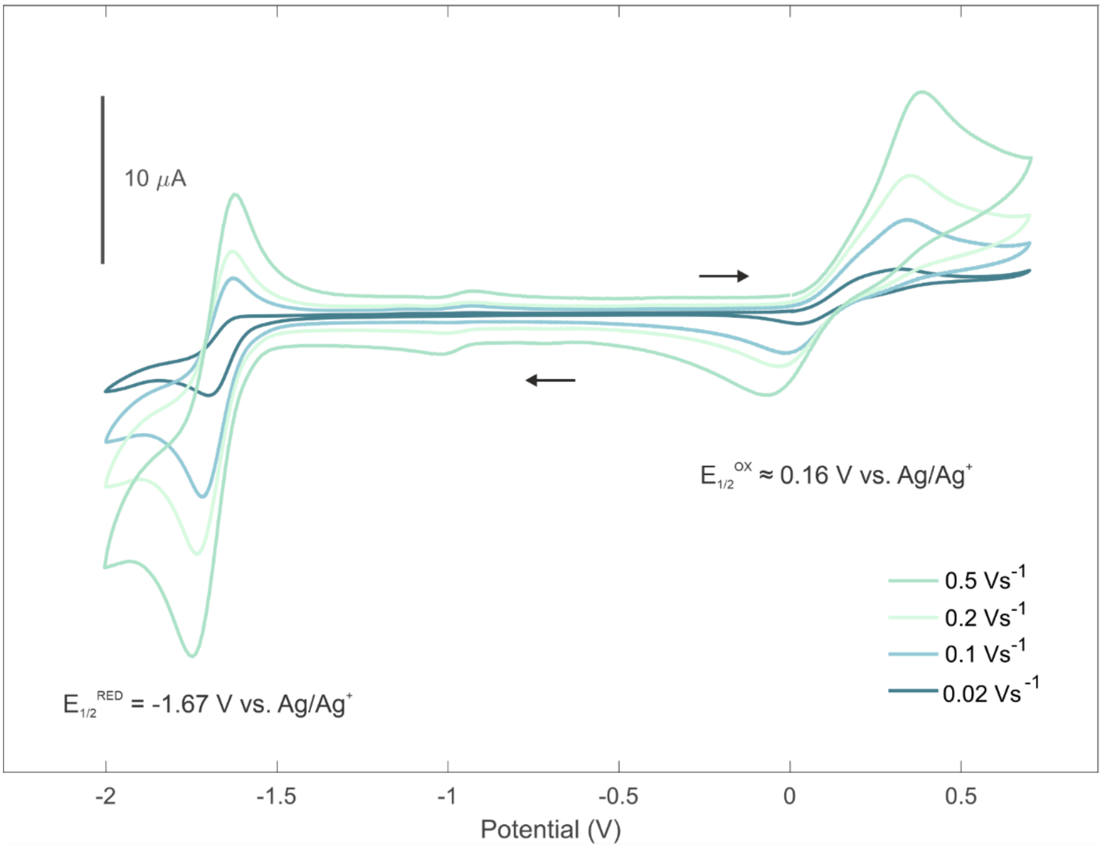

Figure SI37. Cyclic voltammogram of **1b** at different scan rates.

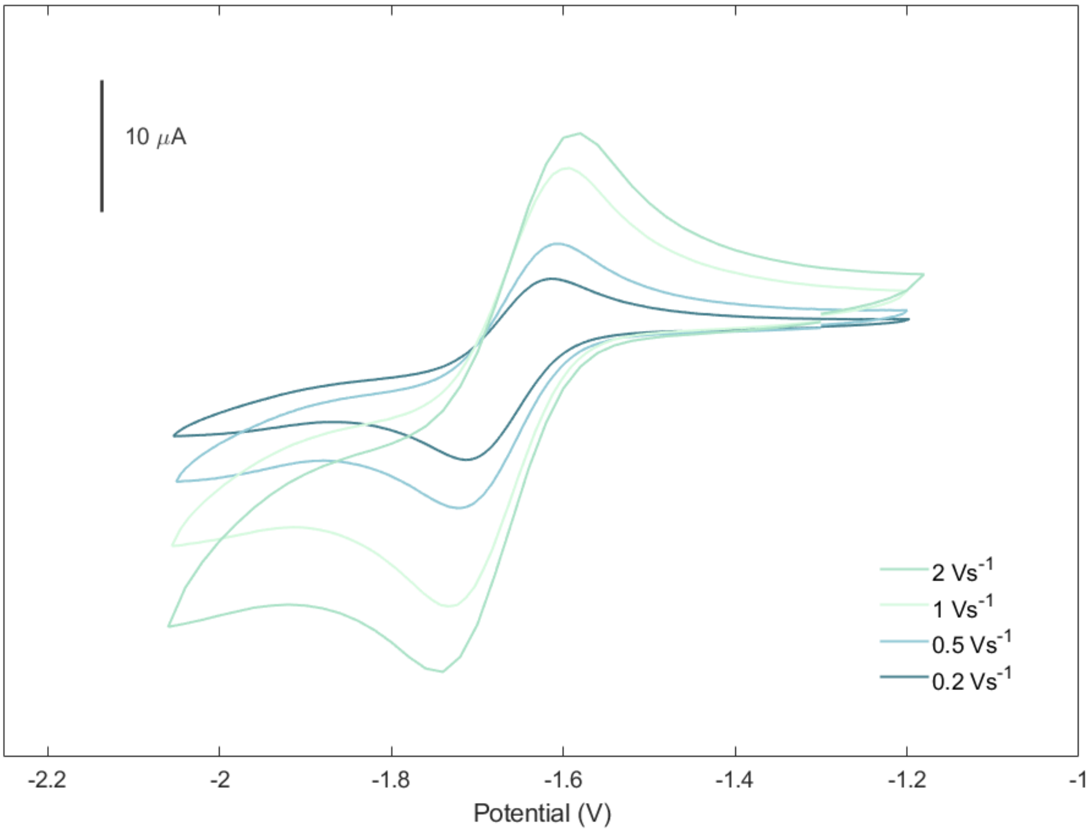

Figure SI38. Cyclic voltammogram of **1b** at different scan rates.

Number of electrons transferred during reduction of **1a**

Randles–Ševčík equation (reversible process)

The Randles–Ševčík equation yields a value of *n* = 0.9 for the number of electrons transferred.[24]

$$I_p = 2.69 \cdot 10^5 \cdot n^{3/2} \cdot A \cdot D^{1/2} \cdot c \cdot v^{1/2}$$

I_p peak current I_p (100 mV) = $1.90 \cdot 10^{-5}$ A

n number of transferred electrons

A electrode surface A = 0.071 cm^2

D diffusion coefficient D = $\frac{k_B T}{6\pi\eta r_H} \approx 5.79 \cdot 10^{-6}$ *cm*^{2}*s*$^{-1}$

with $r_H = 8.05\,Å$ *[molecule diameter = 16.09 Å (r*^{2}*scan-3c/CPCM(thf))]*
and $\eta\ (thf, rt) = 0.4695$ *mPa·s*[25]

c concentration c = 1.99 $\cdot 10^{-6}$ *mol cm*$^{-3}$

v scan rate v = 0.1 Vs^{-1}

Modified Randles–Ševčík equation (quasi-reversible process $\Delta E_p \neq 57.5$ mV)[24]

For quasi-reversible processes ($\Delta E_p \neq 57.5$ mV), the modified Randles–Ševčík equation must be used [ΔE_p = 0.161 V (v = 100 mVs^{-1}), 0.191 V (v = 200 mVs^{-1}), 0.242 V (v = 500 mVs^{-1}), 0.302 V (v = 1 Vs^{-1})].

$$I_p = 2.69 \cdot 10^5 \cdot n^{3/2} \cdot A \cdot D^{1/2} \cdot c \cdot v^{1/2} \cdot K$$

K modified parameter for quasi-reversible reactions

Based on Trachioti, Lazanas and Prodromidis*, K* can be estimated in the range 0.5–0.8 ($\alpha \approx 0.3$, $\log \Lambda \approx 0.16$) yielding corrected values for *n* between 1.20 and 0.87.[24]

Quantum chemistry

Structure optimization/NBO analysis

All structures were fully optimized starting from experimentally determined structures obtained by single-crystal X-ray diffraction (XRD). Structure optimizations were performed using both the BP86 functional and the composite r^{2}SCAN-3c method as implemented in ORCA6.0.1.[26-33] The r^{2}SCAN-3c method is a versatile and computationally efficient approach.[34] This method combines the r^{2}SCAN meta-GGA density functional with a tailored triple-ζ Gaussian basis set (def2-mTZVPP/J)[34], an auxiliary basis set for the resolution-of-the-identity (RI) approximation, the D4 dispersion correction[35] for long-range London dispersion,[36] and a geometric counterpoise correction to account for basis set superposition error (gCP).[37] Solvent effects were modeled using the conductor-like polarizable continuum model (CPCM) as the solvent.[38] The BP86 calculations were conducted using Grimme's D3 dispersion correction, Becke–Johnson damping (D3BJ). The RI approximation was applied to accelerate the calculations. The basis set chosen was def2-TZVP for Ge, B, Cl and def2-SVP on all other elements.[39, 40]

All computations employed *tight* convergence criteria for structure optimizations and *very tight* thresholds for SCF convergence. The integration grid *defgrid3* was used in all cases. All structures

were fully optimized, and harmonic vibrational frequencies were computed to confirm whether the stationary points correspond to minima.

Analyses of the electronic structure were carried out using NBO 6.0 as implemented in ORCA, employing the structures optimized at the BP86 level of theory.[41]

Excited state spectra were computed using time-dependent DFT with the B3LYP functional as implemented in the ORCA with the def2-TZVP basis set and RIJCOSX approximation.[40, 42] For radical species **K[1]**, unrestricted Kohn–Sham (UKS) calculations were employed.

Computational study on EPR parameters of K[**1**˙]

EPR parameters were calculated based on structures optimized with a range of density functionals and dispersion corrections, with solvent effects of diethyl ether considered (see below). The basis set chosen was def2-TZVP for Ge, B, Cl and def2-SVP on all other elements (r^2scan-3c was used with implemented def2-mTZVPP/J basis set).[39, 40] All computations employed default convergence criteria for structure optimizations and *very tight* thresholds for SCF convergence. The integration grid *defgrid3* was used in all cases.

Functionals used: ωB97X[43], ωB97X-V[44], B3LYP[42], CAM-B3LYP,[45] PBE0[46], TPSS[47], BP86[48, 49], and r^2SCAN-3c[34]
Dispersion corrections: D30[30], D3BJ[31], D4[35] and non-local (NL)[50]
Solvation models: CPCM[38] and SMD[51] (diethyl ether)

EPR single-point calculations were performed as implemented in ORCA using the B3LYP functional in combination with the EPR-II[52] basis set (for all compatible atoms), and def2-TZVP[39] (for Ge, K, and Cl). Dispersion interactions were included via the D4 correction[35], and solvation effects of diethyl ether were modeled using CPCM[38] correction.

*Computational study on EPR parameters of K[**1a**˙]*

Hyperfine coupling constants were calculated for Ge, B, K, Cl (considering Fermi contact term (aiso), dipolar coupling (adip), and orbital contributions (aorb)) as well as all hydrogen atoms (only aiso).

Table SI10a. Computation of EPR parameters (g_{iso}, A_{iso}) based on structures obtained by optimization with different density functional methods. The relative percentage deviation to the experimental parameters is given (%dev.) and highlighted in colours (green = low deviation, red = high deviation).

	g_{iso}	% dev.	A_{iso}(Ge)	% dev.	A_{iso} (B)	% dev.
**experiment**	**2.0116**		**-59.64**		**-14.12**	
crystal structure	2.0125	0.043	-35.96	39.7	-35.29	149.9

The significant discrepancy between the calculated EPR parameters when using the crystal structure and the experimentally obtained parameters (Table SI10a) shows the importance of the accuracy of the underlying structure. To systematically investigate the influence of the structure on the calculated EPR parameters, g_{iso} and A_{iso} were computed based on structures optimized with several density functionals, including different dispersion corrections and solvation models.

Table SI10b. Computation of EPR parameters (g_{iso}, A_{iso}) based on structures obtained by optimization with different density functional methods.

structure	g_{iso}	% dev.	A_{iso}(Ge)	% dev.	A_{iso} (B)	% dev.
PBE0/CPCM	2.0125	0.042	-38.25	35.9	-23.3	65
BP86/CPCM	2.0121	0.023	-38.3	35.8	-27.42	94.2
TPSS/CPCM	2.0121	0.027	-38.81	34.9	-24.47	73.3
B3LYP/CPCM	2.0122	0.028	-41.78	29.9	-20.94	48.3
ωB97X/CPCM	2.012	0.022	-46.06	22.8	-11.91	15.6
r2scan-3c/CPCM	2.0117	0.004	-48.5	18.7	-6.96	50.7

All density functionals (Table SI10b) yield g_{iso} values which are in very good agreement with the experimentally determined value of 2.0116. The best agreement is obtained with r2scan-3c (0.004%dev.) and ωB97X (0.022%dev.) functional. The hyperfine coupling constants of the germanium A_{iso}(Ge) display a more pronounced functional dependency. BP86, B3LYP, PBE0 and TPSS consistently underestimate A_{iso}(Ge). The best results were obtained using r^2scan-3c (18.7% dev.) and ωB97X (22.8% dev.) functionals. The boron hyperfine coupling constant is strongly dependent on the utilized functional. Most functionals tend to overestimate A_{iso}(B), while only r^2scan-3c underestimates A_{iso}(B). ωB97X reproduces the experimental value remarkably well (15.6% dev.). The different behavior of r^2scan-3c is probably due to the implementation of D4 dispersion correction, which will be discussed in the next section.

Table SI10c. Computation of EPR parameters (g_{iso}, A_{iso}) based on structures obtained by optimization with different density functional methods.

structure	g_{iso}	% dev.	A_{iso}(Ge)	% dev.	A_{iso} (B)	% dev.
B3LYP/CPCM	2.0122	0.028	-41.78	29.9	-20.94	48.3
B3LYP/D30/CPCM	2.0115	0.006	-54.04	9.4	0.16	101.2
B3LYP/D3BJ/CPCM	2.0115	0.007	-51.20	14.2	-2.22	84.3
B3LYP/D4/CPCM	2.0115	0.003	-50.75	14.9	-3.06	78.3
B3LYP/NL/CPCM	2.0115	0.007	-50.60	15.2	-3.72	73.7
TPSS/CPCM	2.0121	0.027	-38.81	34.9	-24.47	73.3
TPSS/D3BJ/CPCM	2.0113	0.016	-48.49	18.7	-4.82	65.8
TPSS/D4/CPCM	2.0113	0.016	-48.81	18.2	-4.25	69.9
BP86/CPCM	2.0121	0.023	-38.30	35.8	-27.42	94.2
BP86/D4/CPCM	2.0112	0.022	-49.96	16.2	-3.04	78.4
ωB97X/CPCM	2.0120	0.022	-46.06	22.8	-11.91	15.6
ωB97X/D4/CPCM	2.0119	0.015	-47.91	19.7	-8.78	37.8
ωB97X-V/CPCM	2.0116	0.002	-49.75	16.6	-5.01	64.5
r^2scan-3c/CPCM	2.0117	0.004	-48.50	18.7	-6.96	50.7

The implementation of dispersion corrections (D3, D4, NL, VV10) leads to consistent improvements across all investigated functionals (Table SI10c). g_{iso} values generally benefit from dispersion, particularly when using the B3LYP and TPSS functionals. The impact on A_{iso}(Ge) is also substantial, the values improve systematically for all methods studied. B3LYP/D3(0) yields the best agreement with the experimental A_{iso}(Ge) value (9.4%dev.). In contrast, A_{iso}(B) is systematically underestimated upon inclusion of dispersion corrections. The improvement of A_{iso}(Ge) is accompanied by a loss in the accuracy of A_{iso}(B). ωB97X yields a balanced set of parameters with and without dispersive corrections.

Table SI10d. Computation of EPR parameters (g_{iso}, A_{iso}) based on structures obtained by optimization with different density functional methods.

structure	g_{iso}	% dev.	A_{iso}(Ge)	% dev.	A_{iso} (B)	% dev.
B3LYP/CPCM	2.0122	0.028	-41.78	29.9	-20.94	48.3
B3LYP/SMD	2.0117	0.004	-47.78	19.9	-8.06	42.9
ωB97X/CPCM	2.0120	0.022	-46.06	22.8	-11.91	15.6
ωB97X/SMD	2.0121	0.026	-45.12	24.3	-13.26	6.1
r^2scan-3c/CPCM	2.0117	0.004	-48.50	18.7	-6.96	50.7
r^2scan-3c/SMD	2.0120	0.018	-44.21	25.9	-14.23	0.8
ωB97X-V/CPCM	2.0116	0.002	-49.75	16.6	-5.01	64.5
ωB97X-V/SMD	2.0119	0.014	-50.88	14.7	-8.04	43.1

The usage of CPCM generally provides better agreement with the experimental g_{iso} value, although in the case of B3LYP, SMD slightly improves the results (Table SI10d). For A_{iso}(Ge), the use of SMD leads to better results when using B3LYP and ωB97X-V but leads to worse results when using ωB97X and r^2scan-3c. A_{iso}(B) consistently benefits from using SMD instead of CPCM across all tested functionals. The combination of r^2scan-3c and SMD yields excellent agreement with the experimental A_{iso}(B) value (0.8%dev.), while yielding moderately accurate results for A_{iso}(Ge).

Table SI10e. Computation of epr parameters (g_{iso}, A_{iso}) based on structures obtained by optimization with the ωB97X/CPCM method.

structure	single point	g_{iso}	% dev.	A_{iso}(Ge)	% dev.	A_{iso} (B)	% dev.
ωB97X/CPCM	BP86	2.012	0.018	-34.93	41.4	-5.71	59.6
ωB97X/CPCM	B3LYP	2.012	0.022	-46.06	22.8	-11.91	15.6
ωB97X/CPCM	CAM-B3LYP	2.0131	0.075	-54.93	7.9	-20.21	43.1
ωB97X/CPCM	BHLYP	2.0119	0.014	-70.5	18.2	-33.45	136.9

To validate the choice of functional for the EPR single-point calculations, a series of computations were performed based on the ωB97X/CPCM structure using exchange-correlation functionals with varying amounts of exact exchange (BP86: 0%, B3LYP: 20%, CAM-B3LYP: 19% (short range)/ 65% (long range), BHLYP: 50% exact exchange). The results (Table SI10e) illustrate that the range-seperated hybrid functional CAM-B3LYP delivers the most accurate results while BP86 and BHLYP deliver quite imbalanced results.

Table SI10f. Computation of EPR parameters (g_{iso}, A_{iso}) based on structures obtained by optimization with the ωB97X-V/CPCM method and a CAM-B3LYP epr single point.

structure	**single point**	g_{iso}	% dev.	A_{iso}(Ge)	% dev.	A_{iso} (B)	% dev.
ωB97X-V/CPCM	CAM-B3LYP	2.0127	0.056	-58.35	2.2	-14.12	0

Finally, a combined approach was carried out: a CAM-B3LYP single-point calculation was performed on the ωB97X-V/CPCM structure including dispersion corrections (Table SI10f). Despite a slight deterioration in the g_{iso} value, both A_{iso} values are in very good agreement with the experimental reference.

*Computation of EPR parameters of K[**1b**˙]*

Table SI10g. Computation of EPR parameters (g_{iso}, A_{iso}) of **1b**˙⁻ based on structures obtained by optimization with the ωB97X and ωB97X-V functional combined with a B3LYP and CAM-B3LYP epr single point.

geometry	**single point**	g_{iso}	% dev.	A_{iso}(Ge)	% dev.	A_{iso} (B)	% dev.
ωB97X/CPCM	B3LYP	2.013	0.150	-29.99	40.8	-25.78	82.3
ωB97X/CPCM	CAM-B3LYP	2.015	0.072	-37.54	25.9	-33.45	136.6
ωB97X-V/CPCM	B3LYP	2.0124	0.185	-33.93	33.1	-14.88	5.2
ωB97X-V/CPCM	CAM-B3LYP	2.0139	0.112	-41.87	17.4	-20.64	45.9

To evaluate the extent to which the results of the study are transferrable, the EPR parameters of **1b**˙⁻ were calculated using the most promising methods: the ωB97X and ωB97X-V functional combined with a B3LYP and CAM-B3LYP epr single point (Table SI10g). Overall, the values show a larger deviation, however, the results obtained using the ωB97X-V//CAM-B3LYP are still close to the experimentally determined values.

Spin density distribution and Mulliken atomic spin densities of K[**1a**˙]

Spin density distributions and Mulliken atomic spin densities were obtained from the optimized structures from the previous section.

Figure SI25 shows the spin density distribution of K[**1a**˙] obtained at the ωB97X-V/CPCM(Et_2O) level of theory. Independent on the functional, the spin density is mainly localized on the germanium atoms. A smaller negative spin density is localized on the boron atom. A certain spin delocalization is also localized on the xanthene backbone.

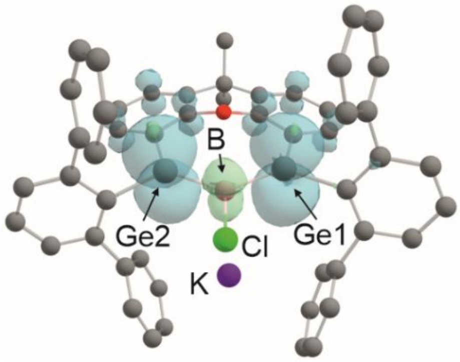

Figure SI39. Spin density distribution for K[**1a**˙] (isosurface = 0.002174, ωB97X-V/CPCM(Et_2O)).

The Mulliken atomic spin densities indicate significant spin density on both Ge centers and a negative spin density at the boron atom - analogous to the allyl radical (Ge 0.575[53]/0.583[54], B -0.170[54]/-0.179[53]). Especially the functional ωB97X-V yields an evenly distributed spin density on both germanium atoms, suggesting a well delocalized electronic structure.

Table SI11. Mulliken atomic spin densities of selected atoms in K[**1a**˙] at different level of theories.

**method**	**ρ(Ge1)**	**ρ(Ge2)**	**ρ(B)**
B3LYP/D4/CPCM	0.548	0.532	-0.248
ωB97X/CPCM	0.580	0.563	-0.341
ωB97X-V/CPCM	0.565	0.563	-0.224
BP86/D4/CPCM	0.536	0.520	-0.219

NMR shielding tensor computations

NMR computations were performed using the revPBE[55] functional in combination with ZORA[56-58] relativistic corrections, the ZORA-TZVP[59] basis set, and the D3(BJ)[31] dispersion correction.

Frontier orbital energies of **1**, **K[1]** and **K_2[1]**

Frontier orbital energies were computed at the r^{2}scan-3c/CPCM(diethylether) level of theory.

Table SI12. Frontier orbital energies of **1**, **K[1]** and **K_2[1]**.

**1a**	Eh	kcal/mol	**1b**	Eh	kcal/mol
HOMO	-0.15	-95.56	HOMO	-0.15	-96.93
LUMO	-0.10	-64.91	LUMO	-0.10	-62.86
LUMO+1	-0.06	-37.99	LUMO+1	-0.05	-33.21

**K[1a]**	Eh	kcal/mol	**K[1b]**	Eh	kcal/mol
HOMO-1	-0.16	-99.03	HOMO-1	-0.15	-93.25
SOMO	-0.11	-71.79	SOMO	-0.11	-67.95
LUMO	-0.05	-33.37	LUMO	-0.05	-30.97

**K_2[1a]**	Eh	kcal/mol	**K_2[1b]**	Eh	kcal/mol
HOMO-1	-0.14	-90.89	HOMO-1	-0.13	-80.25
HOMO	-0.10	-61.13	HOMO	-0.09	-57.57
LUMO	-0.05	-33.69	LUMO	-0.05	-28.88

Redox Potentials

Geometry optimizations were carried out using the r^2SCAN-3c[34] composite method. Solvent effects were included via the conductor-like polarizable continuum model (CPCM)[38] using tetrahydrofuran (THF) as the solvent. All molecules were optimized in the absence of the potassium counterion. The following compounds were computed:

- **1a**, K[**1a**˙], K_2[**1a**]
- **1b**, K[**1b**˙], K_2[**1b**]
- Ferrocene and ferrocenium (Fc and Fc^+)

Standard-free redox potentials (E^0) were derived from the calculated Gibbs free energies (G) according to the equation:

$$E^0{}_{Red/Ox} = -\frac{\Delta G^0{}_{Red/Ox}}{n_e F} - E^0\,(Fc/Fc^+)$$

where n_e is the number of electrons transferred, F is the Faraday constant, and $E^0\,(Fc/Fc^+)$ is the reference potential $E^0(Fc/Fc^+) = -\frac{\Delta G^0(Fc/Fc^+)}{F}$.[60] The redox potentials were subsequently converted to potentials with respect to Ag/Ag^+ by substraction of the empirical value 0.22 V.[61]

The following potentials were obtained for the **1a**/K[**1a**˙]/K_2[**1a**] redox couples:

$E_{1/2}^{RED1}$(r^2scan-3c/CPCM(THF)) = -1.80 V vs. Fc/Fc^+ ≈ -1.58 V vs. Ag/Ag^+

$E_{1/2}^{RED2}$(r^2scan-3c/ /CPCM(THF)) = -2.29 V vs. Fc/Fc^+ ≈ -2.07 V vs. Ag/Ag^+

The following potentials were obtained for the **1b**/K[**1b**˙]/K_2[**1b**] redox couples:

$E_{1/2}^{RED1}$(r^2scan-3c/CPCM(THF)) = -1.94 V vs. Fc/Fc^+ ≈ -1.72 V vs. Ag/Ag^+

$E_{1/2}^{RED2}$(r^2scan-3c/CPCM(THF)) = -2.45 V vs. Fc/Fc^+ ≈ -2.23 V vs. Ag/Ag^+

References

1. R. H. Kern, M. Schneider, K. Eichele, H. Schubert, H. F. Bettinger and L. Wesemann, *Angew. Chem. Int. Ed.*, 2023, **62**, e202301593.
2. W. Haubold and U. Kraatz, *Z. Anorg. Allg. Chem.*, 1976, **421**, 105–110.
3. I. Raabe, K. Wagner, K. Guttsche, M. Wang, M. Grätzel, G. Santiso-Quiñones and I. Krossing, *Chem. Eur. J.*, 2009, **15**, 1966–1976.
4. L. J. Farrugia, *Appl. Crystallogr.*, 1997, **30**, 565.
5. L. J. Farrugia, *Appl. Crystallogr.*, 1999, **32**, 837–838.
6. L. J. Farrugia, *J. Appl. Crystallogr.*, 2012, **45**, 849–854.
7. C. B. Hübschle, G. M. Sheldrick and B. Dittrich, *J Appl Crystallogr*, 2011, **44**, 1281–1284.
8. G. M. Sheldrick, *Journal*, 1997, Program of the Crystal Structure Refinement.
9. G. M. Sheldrick, *Acta Cryst. A*, 2008, **64**, 112–122.
10. *Journal*, 1996.
11. K. Jackowski, W. Makulski, A. Szyprowska, A. Antušek, M. Jaszuński and J. Jusélius, *J. Chem. Phys.*, 2009, **130**, 044309.
12. N. F. Ramsey, *Phys. Rev.*, 1951, **83**, 540–541.
13. N. F. Ramsey, *Phys. Rev.*, 1950, **78**, 699–703.
14. C. J. Jameson and H. S. Gutowsky, *J. Chem. Phys.*, 1964, **40**, 1714–1724.
15. J. B. Grutzner, in *Recent Advances in Organic NMR Spectroscopy*, eds. J. B. Lambert and R. Rittner, Norell Press, Landisville, NJ, 1987, pp. 17–42.
16. G. A. Olah, G. K. S. Prakash, K. Wade, A. Molnar and R. E. Williams, in *Hypercarbon Chemistry*, John Wiley & Sons, Ltd., Hoboken, NJ, 2011, DOI: https://doi.org/10.1002/9781118016466.ch5, ch. 5, pp. 185–293.
17. C. Mealli and D. M. Proserpio, *J. Chem. Educ.*, 1990, **67**, 399.
18. W. L. S. Jorgensen, L. , *The Organic Chemist's Book of Orbitals*, Academic Press, New York, 1973.
19. G. Olah and H. Mayr, *J. Am. Chem. Soc.*, 1976, **98**, 7333–7340.
20. H. Mayr and G. A. Olah, *J. Am. Chem. Soc.*, 1977, **99**, 510–513.
21. J. P. C. M. van Dongen, H. W. D. van Dijkman and M. J. A. de Bie, *Recueil des Travaux Chimiques des Pays-Bas*, 1974, **93**, 29–32.
22. D. H. O'Brien, A. J. Hart and C. R. Russell, *J. Am. Chem. Soc.*, 1975, **97**, 4410–4412.
23. S. Stoll and A. Schweiger, *Journal of Magnetic Resonance*, 2006, **178**, 42–55.
24. M. G. Trachioti, A. C. Lazanas and M. I. Prodromidis, *Michrochim. Acta*, 2023, **190**, 251.
25. W. Hayduk, H. Laudie and O. H. Smith, *Journal of Chemical & Engineering Data*, 1973, **18**, 373–376.
26. F. Neese, *WIREs Comput. Mol. Sci.*, 2022, **12**, e1606.
27. F. Neese, *J. Comput. Chem.*, 2003, **24**, 1740–1747.
28. D. Bykov, T. Petrenko, R. Izsák, S. Kossmann, U. Becker, E. Valeev and F. Neese, *Molecular Physics*, 2015, **113**, 1961–1977.
29. F. Neese, *J. Comput. Chem.*, 2023, **44**, 381–396.
30. S. Grimme, J. Antony, S. Ehrlich and H. Krieg, *J. Chem. Phys.*, 2010, **132**, 154104–154119.
31. S. Grimme, S. Ehrlich and L. Goerigk, *J. Comput. Chem.*, 2011, **32**, 1456–1465.
32. F. Neese, *WIREs Comput. Mol. Sci.*, 2012, **2**, 73–78.
33. F. Neese, F. Wennmohs, U. Becker and C. Riplinger, *J. Chem. Phys.*, 2020, **152**, 224108.
34. S. Grimme, A. Hansen, S. Ehlert and J.-M. Mewes, *J. Chem. Phys.*, 2021, **154**, 064103.
35. E. Caldeweyher, C. Bannwarth and S. Grimme, *J. Chem. Phys.*, 2017, **147**, 034112.
36. E. Caldeweyher, S. Ehlert, A. Hansen, H. Neugebauer, S. Spicher, C. Bannwarth and S. Grimme, *J. Chem. Phys.*, 2019, **150**, 154122.
37. H. Kruse and S. Grimme, *J. Chem. Phys.*, 2012, **136**, 154101.
38. V. Barone and M. Cossi, *J. Phys. Chem. A*, 1998, **102**, 1995–2001.
39. F. Weigend and R. Ahlrichs, *Phys. Chem. Chem. Phys.*, 2005, **7**, 3297–3305.

40. F. Weigend, *Phys. Chem. Chem. Phys.*, 2006, **8**, 1057–1065.
41. E. D. Glendening, J. K. Badenhoop, A. E. Reed, J. E. Carpenter, J. A. Bohmann, C. M. Morales, P. Karafiloglou, C. R. Landis and F. Weinhold, *Journal*, 2018.
42. A. D. Becke, *J. Chem. Phys.*, 1993, **98**, 5648–5652.
43. J.-D. Chai and M. Head-Gordon, *J. Chem. Phys.*, 2008, **128**.
44. N. Mardirossian and M. Head-Gordon, *Phys. Chem. Chem. Phys.*, 2014, **16**, 9904–9924.
45. T. Yanai, D. P. Tew and N. C. Handy, *Chem. Phys. Lett.*, 2004, **393**, 51–57.
46. C. Adamo and V. Barone, *J. Chem. Phys.*, 1999, **110**, 6158–6170.
47. J. Tao, J. P. Perdew, V. N. Staroverov and G. E. Scuseria, *Phys. Rev. Lett.*, 2003, **91**, 146401.
48. A. D. Becke, *Phys. Rev. A*, 1988, **38**, 3098–3100.
49. J. P. Perdew and W. Yue, *Phys. Rev. B*, 1986, **33**, 8800–8802.
50. W. Hujo and S. Grimme, *Journal of Chemical Theory and Computation*, 2011, **7**, 3866–3871.
51. A. V. Marenich, C. J. Cramer and D. G. Truhlar, *The Journal of Physical Chemistry B*, 2009, **113**, 6378–6396.
52. V. Barone, in *Recent Advances in Density Functional Methods*, ed. D. P. Chong, World Scientific, Singapore, 1995, vol. Part 1, p. 287.
53. F. Aquilante, K. P. Jensen and B. O. Roos, *Chem. Phys. Lett.*, 2003, **380**, 689–698.
54. D. Lazdins and M. Karplus, *J. Chem. Phys.*, 1966, **44**, 1600–1611.
55. Y. Zhang and W. Yang, *Phys. Rev. Lett.*, 1998, **80**, 890–890.
56. E. v. Lenthe, E. J. Baerends and J. G. Snijders, *J. Chem. Phys.*, 1993, **99**, 4597–4610.
57. E. v. Lenthe, E. J. Baerends and J. G. Snijders, *J. Chem. Phys.*, 1994, **101**, 9783–9792.
58. J. Radnik and H.-J. Ernst, *J. Chem. Phys.*, 1999, **110**, 10522–10525.
59. A. Schäfer, C. Huber and R. Ahlrichs, *J. Chem. Phys.*, 1994, **100**, 5829–5835.
60. H. Neugebauer, F. Bohle, M. Bursch, A. Hansen and S. Grimme, *J. Phys. Chem. A*, 2020, **124**, 7166–7176.
61. S. E. A. Jaspars, Master Thesis Master Thesis, Eindhoven university of technology, 2021.
