# Supplementary material for: Three reversibly interconvertible redox states of boradigermaallyl: syntheses of radical allyl anion and allyl dianion
Source: Chem Sci. 2026 Mar 2;17(17):8622–9. doi: 10.1039/d6sc00727a (PMC12969875; doi:10.1039/d6sc00727a)
Supplement: SC-017-D6SC00727A-s002 [file SC-017-D6SC00727A-s002.pdf]

Supplementary Information (SI) for Chemical Science.
This journal is © The Royal Society of Chemistry 2026

Three Reversibly Interconvertible Redox States of Boradigermaallyl: Syntheses of Radical Allyl Anion and Allyl Dianion

Stefan F. Miehe,[a] Klaus Eichele,[a] Hartmut Schubert,[a] Holger F. Bettinger,[b] Christian P. Sindlinger*[c] and Lars Wesemann*[a]

[a]Institut für Anorganische Chemie, Eberhard Karls Universität Tübingen, Auf der Morgenstelle 18, 72076 Tübingen, Germany. E-mail: lars.wesemann@uni-tuebingen.de
[b]Institut für Organische Chemie, Eberhard Karls Universität Tübingen, Auf der Morgenstelle 18, 72076 Tübingen, Germany. E-mail: holger.bettinger@uni-tuebingen.de
[c]Institut für Anorganische Chemie, Universität Stuttgart, Pfaffenwaldring 55, 70569 Stuttgart, Germany. E-mail: christian.sindlinger@iac.uni-stuttgart.de

Supporting Information **B**

Coordinates of optimized structures

K[1a$^{\cdot}$]_ωB97X-V_RIJCOSX_ CPCM(Et_2O) (epr study)

* xyz 0 2

C	-0.946012000	-1.468753000	-5.392377000
C	-0.366332000	0.699192000	-6.518395000
C	-0.489991000	-0.006592000	-5.164337000
C	2.105021000	0.214960000	-4.952488000
C	-2.539397000	1.538138000	-4.667386000
C	0.838578000	-0.007239000	-4.397288000
C	-1.519063000	0.674209000	-4.251230000
C	3.242700000	0.228153000	-4.133930000
C	-3.481558000	2.013084000	-3.746042000
C	-4.987039000	-1.069155000	-3.832947000
C	3.519111000	-3.349562000	-2.856820000
C	0.779984000	-0.218586000	-3.015567000
C	-1.462810000	0.368700000	-2.885262000
C	3.128880000	0.058465000	-2.751812000
C	-3.403067000	1.654159000	-2.397450000
C	-6.568827000	-2.205109000	-2.261009000
C	5.782319000	-3.698580000	-1.785032000
C	-5.259918000	-1.400745000	-2.359278000
C	4.416604000	-3.003948000	-1.660639000
C	1.874939000	-0.151759000	-2.148906000
C	1.296336000	3.229337000	-2.186074000
C	-2.358039000	0.842017000	-1.922755000
C	3.697728000	4.057896000	-2.201750000
C	-0.023260000	-4.389578000	-2.707845000
C	-3.377643000	-3.078948000	-2.452326000
C	-1.509817000	-4.772395000	-2.767853000
C	-4.114364000	-2.157199000	-1.689193000
C	2.663252000	3.175386000	-1.488165000
C	3.274278000	-4.636002000	-0.122382000
C	-3.042358000	4.494085000	-0.464282000
C	3.764327000	-3.336644000	-0.321327000
C	-2.348625000	-3.842404000	-1.902883000

C	3.330167000	-7.493244000	1.122598000
C	-5.293579000	4.708659000	0.690536000
C	-1.724891000	-6.245192000	-2.390348000
C	-4.030051000	3.851867000	0.520681000
C	-3.789244000	-1.983994000	-0.324917000
C	2.719936000	-5.039118000	1.094466000
C	2.210000000	-6.458964000	1.303358000
C	1.015584000	-6.770027000	0.391524000
C	3.678894000	-2.397181000	0.730769000
C	2.570245000	3.524837000	-0.010911000
C	-2.102506000	-3.722065000	-0.529344000
C	4.216815000	-0.993415000	0.589394000
C	-4.427068000	-0.899686000	0.512418000
C	5.605304000	-0.795072000	0.644933000
C	3.340707000	0.117918000	0.511034000
C	2.015930000	4.762044000	0.355895000
C	6.138230000	0.493632000	0.654345000
C	-5.741238000	-1.044338000	0.978461000
C	-3.664720000	0.238037000	0.891887000
C	-2.802824000	-2.817034000	0.272645000
C	3.882278000	1.417092000	0.677780000
C	5.276729000	1.589428000	0.714299000
C	2.662959000	-4.101125000	2.131774000
C	3.074831000	2.660661000	0.988219000
C	-6.322445000	-0.068397000	1.789123000
C	-3.386956000	3.563131000	1.871757000
C	3.132044000	-2.790096000	1.983335000
C	-4.217753000	1.155460000	1.815608000
C	0.215288000	7.002350000	1.283783000
C	-5.558145000	1.016653000	2.214393000
C	2.587619000	7.599962000	1.927606000
C	-2.771248000	4.619080000	2.563156000
C	1.958961000	5.184284000	1.689984000
C	-3.435939000	2.278240000	2.460924000
C	-2.532666000	-2.773012000	1.777153000
C	1.456215000	6.570045000	2.074564000
C	2.979834000	3.048302000	2.350776000
C	-1.172713000	-3.347147000	2.185818000
C	3.102927000	-1.837274000	3.182914000
C	2.436200000	4.303785000	2.670292000
C	-3.653858000	-3.498918000	2.541765000
C	-2.183553000	4.440928000	3.821087000
C	-2.849127000	2.076280000	3.740641000
C	4.492696000	-1.724088000	3.833882000
C	2.069721000	-2.208067000	4.253207000
C	3.499422000	2.168583000	3.488718000
C	-1.604821000	5.635352000	4.569997000
C	-2.222334000	3.157200000	4.381810000
C	-2.943867000	0.738124000	4.478571000
C	4.801516000	2.737737000	4.073021000
C	-2.703742000	6.307427000	5.409386000
C	2.451940000	1.961820000	4.593274000
C	-4.243647000	0.655518000	5.300385000

C	-0.391443000	5.285149000	5.438191000
C	-1.760563000	0.463872000	5.420517000
K	-0.211253000	2.964867000	1.769531000
Ge	1.534839000	-0.196788000	-0.224431000
Ge	-1.946383000	0.539422000	-0.032417000
Cl	-0.077648000	0.006931000	2.517228000
O	-0.445013000	-0.438117000	-2.420727000
H	-0.195897000	-2.014934000	-5.990114000
H	-1.908566000	-1.484937000	-5.932431000
H	0.362855000	0.179532000	-7.160228000
H	-1.330867000	0.685786000	-7.050494000
H	-1.081870000	-1.998961000	-4.435310000
H	2.214744000	0.386420000	-6.027348000
H	-2.621441000	1.829155000	-5.718839000
H	-0.045078000	1.748013000	-6.402035000
H	4.228069000	0.399060000	-4.580943000
H	-5.723208000	-0.332668000	-4.197716000
H	-4.291306000	2.666417000	-4.088602000
H	3.983524000	-2.998364000	-3.794620000
H	-5.080115000	-1.966066000	-4.469989000
H	3.365114000	-4.439058000	-2.948378000
H	-3.981331000	-0.647412000	-3.977340000
H	2.531696000	-2.868274000	-2.767608000
H	6.276052000	-3.418629000	-2.731865000
H	-7.401513000	-1.647495000	-2.724266000
H	1.382860000	2.855944000	-3.221581000
H	-6.464305000	-3.165433000	-2.796388000
H	3.805064000	3.744460000	-3.254780000
H	5.667229000	-4.796909000	-1.774883000
H	-3.599462000	-3.192780000	-3.518936000
H	0.129391000	-3.334794000	-2.994661000
H	-1.851522000	-4.637397000	-3.810946000
H	4.027880000	0.132689000	-2.128718000
H	0.572752000	-5.020972000	-3.390254000
H	4.593855000	-1.918185000	-1.684439000
H	-5.395308000	-0.449566000	-1.815158000
H	-3.516194000	4.633730000	-1.450357000
H	0.901174000	4.260184000	-2.231095000
H	-4.157442000	2.035179000	-1.701229000
H	3.340827000	-5.356990000	-0.945979000
H	3.395146000	5.120066000	-2.192153000
H	6.453338000	-3.423501000	-0.953912000
H	0.557200000	2.594725000	-1.663481000
H	-6.846971000	-2.431795000	-1.220933000
H	3.709027000	-7.490284000	0.085634000
H	-5.787900000	4.865997000	-0.283322000
H	3.024315000	2.141273000	-1.551436000
H	4.686977000	3.980687000	-1.718460000
H	0.385172000	-4.518415000	-1.689841000
H	-1.138129000	-6.907677000	-3.050135000
H	-2.155832000	3.850785000	-0.616875000
H	2.960656000	-8.509625000	1.341680000
H	-2.702505000	5.484717000	-0.114857000

H	1.305826000	-6.726977000	-0.673067000
H	-4.345170000	2.884598000	0.100026000
H	-2.787841000	-6.526932000	-2.476431000
H	4.179203000	-7.283130000	1.794523000
H	-5.049118000	5.699863000	1.111430000
H	1.650898000	5.425474000	-0.436053000
H	0.621196000	-7.781164000	0.592540000
H	-6.014940000	4.219870000	1.367212000
H	6.270226000	-1.664994000	0.692729000
H	-1.406171000	-6.436490000	-1.351205000
H	0.448730000	7.167909000	0.218223000
H	-6.314381000	-1.936724000	0.708776000
H	7.222940000	0.644015000	0.667898000
H	-1.321504000	-4.340864000	-0.078499000
H	2.910967000	7.671987000	0.874306000
H	0.198370000	-6.046054000	0.548814000
H	1.858594000	-6.521845000	2.349454000
H	5.686181000	2.601893000	0.810300000
H	-7.362290000	-0.171508000	2.116900000
H	-2.768900000	5.620913000	2.116310000
H	-0.589955000	6.248797000	1.339076000
H	-0.183949000	7.948810000	1.685123000
H	2.239837000	-4.416398000	3.091000000
H	-0.345278000	-2.880356000	1.624989000
H	-5.997689000	1.766330000	2.882000000
H	2.250544000	8.599233000	2.251786000
H	-2.542161000	-1.714132000	2.085941000
H	3.464136000	7.317447000	2.534004000
H	2.824622000	-0.841805000	2.799189000
H	-1.125323000	-4.440175000	2.033096000
H	5.256757000	-1.374110000	3.122921000
H	-4.644710000	-3.068931000	2.325632000
H	1.071421000	-2.371688000	3.819692000
H	-3.678337000	-4.567790000	2.263755000
H	1.181343000	6.526458000	3.144571000
H	3.728774000	1.177432000	3.066825000
H	-1.278479000	6.364706000	3.805718000
H	-1.004013000	-3.160517000	3.260040000
H	-2.965813000	-0.057605000	3.713702000
H	4.813958000	-2.705537000	4.226218000
H	5.577393000	2.846315000	3.297642000
H	-3.556795000	6.611215000	4.780209000
H	2.403989000	4.617938000	3.720402000
H	2.362916000	-3.116022000	4.808836000
H	-3.483533000	-3.434173000	3.631055000
H	4.463669000	-1.013434000	4.679311000
H	1.515291000	1.544449000	4.186025000
H	-5.143528000	0.744170000	4.676034000
H	1.989034000	-1.386334000	4.985889000
H	0.379418000	4.743197000	4.863866000
H	-0.788320000	0.675951000	4.951012000
H	4.630023000	3.730445000	4.525801000
H	-1.776592000	2.994700000	5.367257000

H	-2.312704000	7.205078000	5.917983000
H	5.193655000	2.069509000	4.859033000
H	-3.079830000	5.612355000	6.180512000
H	2.215880000	2.903062000	5.120114000
H	0.067843000	6.204012000	5.839361000
H	2.833145000	1.250264000	5.346012000
H	-4.268489000	1.461104000	6.055738000
H	-4.293136000	-0.309920000	5.832962000
H	-1.768133000	-0.596879000	5.723420000
H	-0.674777000	4.655855000	6.299102000
H	-1.833586000	1.065657000	6.343151000
B	-0.182318000	0.049242000	0.662338000

*

K[1b$^{\cdot}$]_ωB97X-V_RIJCOSX_CPCM(Et$_2$O) (epr study)

```
* xyz 0 2
C    -0.891179000    1.578896000   -6.552823000
C     1.740309000    1.019833000   -5.302619000
C    -0.718432000    1.841265000   -5.056596000
C    -0.773366000    3.365918000   -4.807998000
C    -3.052357000    0.733475000   -4.700148000
C     2.894116000    0.525115000   -4.693822000
C     0.613506000    1.300120000   -4.532942000
C    -1.820094000    1.177698000   -4.225107000
C     3.809159000   -3.917990000   -3.739360000
C    -3.998461000    0.215920000   -3.815487000
C     1.455394000   -3.002826000   -3.641174000
C     2.913017000    0.230232000   -3.335987000
C     0.710026000    1.053413000   -3.167604000
C     2.784472000   -3.152103000   -2.893352000
C    -4.673194000    3.403476000   -2.824047000
C     3.447817000    3.593820000   -2.743772000
C    -1.588711000    1.028784000   -2.861331000
C    -3.727553000    0.118767000   -2.454292000
C     1.785040000    0.444764000   -2.530576000
C     5.788304000    3.548709000   -1.827166000
C     4.397727000    2.901767000   -1.758965000
C    -2.481903000    0.500375000   -1.940319000
C     2.617841000   -3.798705000   -1.529913000
C     1.970670000   -5.031594000   -1.447342000
C    -2.233006000   -3.371329000   -1.707276000
C    -0.016934000   -7.278494000   -1.031725000
C    -5.069399000    3.150036000   -1.365132000
C    -4.605551000   -3.807225000   -0.959588000
C    -6.347835000    3.937044000   -1.039306000
C     5.358659000   -2.109135000   -0.491714000
C     6.211655000   -1.020992000   -0.370139000
C    -3.278722000    4.714819000   -0.577402000
C    -3.326336000   -2.999477000   -0.702463000
C     2.294024000   -8.151249000   -0.546038000
C     1.235222000   -7.105728000   -0.168816000
C     3.972465000   -1.949669000   -0.402162000
C     1.835144000   -5.711693000   -0.242312000
C    -3.962220000    3.509057000   -0.384774000
C     3.144605000   -3.211672000   -0.367657000
C     3.872669000    2.920674000   -0.330651000
C     5.684717000    0.231143000   -0.080808000
C    -0.132889000    6.206596000   -0.447057000
C     3.427252000   -0.660127000   -0.245031000
C    -1.569250000    6.466198000    0.026065000
C     4.304074000    0.421189000   -0.004913000
C    -2.300468000    5.162058000    0.296248000
C     3.498889000    4.155667000    0.213551000
C     3.811282000    1.767113000    0.472164000
C    -3.629088000    2.701724000    0.714399000
C    -1.579082000    7.407984000    1.233874000
```

C	2.346606000	-5.108043000	0.903119000
C	2.996298000	-3.874564000	0.868392000
C	-2.899500000	-3.148516000	0.747912000
C	-3.554253000	0.172889000	0.892813000
C	-4.295507000	1.374141000	0.969068000
C	-2.148193000	-4.250392000	1.138307000
C	-2.021230000	4.375950000	1.417898000
C	-5.622335000	1.351482000	1.398374000
C	-4.133514000	-1.005760000	1.396985000
C	3.083755000	4.291203000	1.532909000
C	-6.233549000	0.151956000	1.743231000
C	-2.663090000	3.161059000	1.645274000
C	-5.481571000	-1.013769000	1.767528000
C	-3.312124000	-2.222239000	1.723347000
C	3.386018000	1.886212000	1.819043000
C	2.836082000	5.672692000	2.115364000
C	0.147540000	-1.066930000	1.865008000
C	3.584726000	-3.317465000	2.160211000
C	-1.826457000	-7.001259000	2.563579000
C	3.018269000	3.136261000	2.313529000
C	4.942342000	-3.967620000	2.462796000
C	4.137697000	6.212966000	2.724705000
C	-1.810819000	-4.488871000	2.471163000
C	3.424163000	0.695061000	2.770266000
C	-1.002596000	-5.730564000	2.814157000
C	-2.386201000	2.399085000	2.937798000
C	4.840601000	0.514379000	3.337200000
C	-2.970433000	-2.433233000	3.070899000
C	1.708501000	5.712268000	3.148138000
C	2.655601000	-3.483254000	3.368958000
C	-0.970343000	2.582588000	3.484926000
C	-2.239003000	-3.572333000	3.421149000
C	-3.419642000	2.791113000	4.003827000
C	2.420600000	0.774037000	3.922054000
C	-3.381419000	-1.470297000	4.182692000
C	-0.439316000	-5.736809000	4.234267000
C	-4.498074000	-2.077984000	5.042073000
C	-2.204298000	-1.049602000	5.071404000
Ge	1.549564000	-0.250166000	-0.704026000
Ge	-1.840384000	0.255241000	-0.097105000
K	0.489288000	2.904964000	-0.048979000
O	-0.355790000	1.403993000	-2.356201000
H	-0.104183000	2.080953000	-7.121846000
H	-1.847010000	1.979236000	-6.901628000
H	-0.857151000	0.508741000	-6.779921000
H	1.731453000	1.189284000	-6.374492000
H	-3.291156000	0.800708000	-5.756709000
H	0.030099000	3.865121000	-5.359884000
H	-1.734522000	3.766734000	-5.147116000
H	3.778429000	0.336741000	-5.296927000
H	3.964574000	-3.414946000	-4.700341000
H	1.608215000	-2.440464000	-4.569179000
H	-4.962598000	-0.110299000	-4.196314000

H	3.463495000	-4.937937000	-3.942311000
H	1.030393000	-3.977520000	-3.905217000
H	-0.660203000	3.600599000	-3.744609000
H	3.829281000	3.490836000	-3.764638000
H	-5.424744000	2.971708000	-3.492473000
H	4.774272000	-3.983647000	-3.225469000
H	0.723086000	-2.462303000	-3.031834000
H	-4.617895000	4.475363000	-3.042334000
H	-3.707469000	2.950175000	-3.062173000
H	6.184917000	3.488107000	-2.846037000
H	3.813893000	-0.196581000	-2.901983000
H	3.181326000	-2.150223000	-2.729974000
H	3.355701000	4.664228000	-2.530718000
H	2.449406000	3.144161000	-2.722036000
H	1.585635000	-5.478861000	-2.360404000
H	0.220493000	-7.206230000	-2.098261000
H	-2.550034000	-3.094236000	-2.719211000
H	-4.951820000	-3.661923000	-1.989040000
H	4.496166000	1.858476000	-2.066536000
H	-4.491103000	-0.279552000	-1.789539000
H	-7.152981000	3.653891000	-1.725709000
H	-2.029659000	-4.448136000	-1.709508000
H	5.738396000	4.606469000	-1.545469000
H	-3.527421000	5.333237000	-1.436270000
H	-0.770318000	-6.519293000	-0.802018000
H	6.494897000	3.053740000	-1.154439000
H	2.615519000	-8.010105000	-1.584245000
H	-6.170043000	5.012756000	-1.151315000
H	-0.460293000	-8.264397000	-0.858228000
H	-5.284266000	2.083182000	-1.254243000
H	-4.420337000	-4.877177000	-0.808682000
H	-2.100459000	6.962882000	-0.794648000
H	-1.299767000	-2.843336000	-1.483356000
H	7.286324000	-1.156226000	-0.453311000
H	5.768076000	-3.104074000	-0.648888000
H	0.370572000	7.144529000	-0.701989000
H	-3.569132000	-1.947309000	-0.862351000
H	1.889460000	-9.164350000	-0.448682000
H	-5.408797000	-3.502691000	-0.280050000
H	-0.118645000	5.570200000	-1.341089000
H	3.572533000	5.050001000	-0.402818000
H	6.351417000	1.069493000	0.105276000
H	-6.691444000	3.758855000	-0.017029000
H	3.176470000	-8.068664000	0.096133000
H	0.952707000	-7.281769000	0.877116000
H	-1.114400000	8.364041000	0.972452000
H	-1.815196000	-4.957089000	0.382485000
H	-2.601465000	7.603320000	1.571055000
H	0.456864000	5.734337000	0.349360000
H	2.561565000	6.331625000	1.281568000
H	-6.177490000	2.281842000	1.475121000
H	-2.192743000	-7.045319000	1.533579000
H	2.251289000	-5.630782000	1.852892000

H	5.653279000	-3.817538000	1.645971000
H	4.940571000	6.237712000	1.981680000
H	-1.018976000	6.984454000	2.074025000
H	-7.277517000	0.136624000	2.043291000
H	-5.928785000	-1.941742000	2.115261000
H	3.753479000	-2.246140000	2.015429000
H	-0.331479000	-2.052821000	1.920249000
H	-1.303784000	4.737652000	2.149854000
H	3.179540000	-0.194876000	2.186294000
H	1.201203000	-1.198666000	2.134993000
H	5.584051000	0.391999000	2.545721000
H	-1.220456000	-7.895028000	2.748641000
H	-0.154118000	-5.742757000	2.117926000
H	4.820211000	-5.046435000	2.613881000
H	3.990510000	7.227645000	3.108773000
H	-0.211804000	2.335462000	2.732328000
H	-0.310351000	-0.458044000	2.654732000
H	-2.693243000	-7.031383000	3.233958000
H	-2.510452000	1.332878000	2.732820000
H	0.782429000	5.282972000	2.753014000
H	1.641152000	-3.136481000	3.152414000
H	4.461727000	5.575934000	3.555332000
H	5.374056000	-3.543073000	3.376150000
H	2.710392000	3.218076000	3.350927000
H	1.503016000	6.746499000	3.441054000
H	-0.787185000	3.600825000	3.844018000
H	5.120951000	1.386047000	3.940300000
H	2.596973000	-4.529187000	3.688276000
H	4.877526000	-0.371855000	3.980917000
H	1.407761000	0.970039000	3.563906000
H	-4.440119000	2.612087000	3.652314000
H	-3.775034000	-0.560333000	3.720137000
H	1.974304000	5.157963000	4.054047000
H	-3.325564000	3.854016000	4.255453000
H	3.044018000	-2.905151000	4.215100000
H	-0.816101000	1.906280000	4.332021000
H	-5.370244000	-2.341140000	4.436252000
H	0.214496000	-6.604393000	4.370334000
H	-1.987939000	-3.727817000	4.466007000
H	2.408661000	-0.182801000	4.454971000
H	2.690842000	1.548928000	4.647908000
H	-1.391956000	-0.616150000	4.481089000
H	0.144398000	-4.835123000	4.443129000
H	-1.240143000	-5.805968000	4.979192000
H	-3.264032000	2.206925000	4.917684000
H	-4.146851000	-2.988170000	5.541376000
H	-1.798285000	-1.896234000	5.634814000
H	-4.816946000	-1.369304000	5.814240000
H	-2.537912000	-0.299253000	5.796890000
B	-0.044296000	-0.413073000	0.411981000

*

K[1a$^{\cdot}$]_B3LYP_RI_D4_CPCM(Et_2O) (spin density)

```
* xyz 0 2
C    -0.945604000   -1.418333000   -5.349835000
C    -0.331530000    0.751472000   -6.455269000
C    -0.470972000    0.038524000   -5.106425000
C     2.126401000    0.192278000   -4.894567000
C    -2.517774000    1.585899000   -4.616447000
C     0.854150000    0.008347000   -4.336712000
C    -1.497471000    0.723421000   -4.195767000
C     3.264826000    0.171953000   -4.077581000
C    -3.467810000    2.056192000   -3.700533000
C    -4.929897000   -1.019477000   -3.880964000
C     3.482876000   -3.374289000   -2.807082000
C     0.791734000   -0.198042000   -2.956003000
C    -1.448624000    0.418351000   -2.830979000
C     3.148901000    0.011975000   -2.695393000
C    -3.396614000    1.697184000   -2.351973000
C    -6.511230000   -2.205389000   -2.344978000
C     5.725864000   -3.780840000   -1.708421000
C    -5.210741000   -1.381503000   -2.415833000
C     4.376483000   -3.049824000   -1.602649000
C     1.889864000   -0.155406000   -2.091175000
C     1.471189000    3.230219000   -2.209762000
C    -2.350160000    0.890423000   -1.875129000
C     3.900887000    3.976040000   -2.265206000
C     0.021070000   -4.408783000   -2.792191000
C    -3.321553000   -3.042952000   -2.511183000
C    -1.475776000   -4.751005000   -2.832240000
C    -4.060142000   -2.125890000   -1.748235000
C     2.840303000    3.152884000   -1.518619000
C     3.173724000   -4.630117000   -0.058515000
C    -3.275679000    4.540388000   -0.380961000
C     3.702729000   -3.349732000   -0.269988000
C    -2.286547000   -3.801322000   -1.967010000
C     3.174002000   -7.456784000    1.314823000
C    -5.547184000    4.645043000    0.754229000
C    -1.728455000   -6.218705000   -2.450877000
C    -4.245334000    3.845451000    0.586341000
C    -3.730704000   -1.949174000   -0.384623000
C     2.599583000   -5.001669000    1.159159000
C     2.056840000   -6.403406000    1.386379000
C     0.912676000   -6.741504000    0.420045000
C     3.634834000   -2.394275000    0.769398000
C     2.766008000    3.543104000   -0.053888000
C    -2.023478000   -3.664783000   -0.599016000
C     4.218684000   -1.020673000    0.615082000
C    -4.406871000   -0.906995000    0.459394000
C     5.613807000   -0.872933000    0.645532000
C     3.384116000    0.121346000    0.544610000
C     2.270076000    4.810393000    0.287209000
C     6.193484000    0.394482000    0.631862000
C    -5.730368000   -1.099498000    0.879446000
```

C	-3.690753000	0.239527000	0.895413000
C	-2.726353000	-2.765031000	0.205492000
C	3.973409000	1.401452000	0.678397000
C	5.374063000	1.521192000	0.689249000
C	2.565790000	-4.050578000	2.184650000
C	3.223749000	2.675771000	0.966228000
C	-6.367565000	-0.162174000	1.691679000
C	-3.602165000	3.560052000	1.935246000
C	3.073967000	-2.756939000	2.025078000
C	-4.303988000	1.124766000	1.810852000
C	0.270987000	6.869595000	1.510442000
C	-5.651609000	0.936353000	2.162149000
C	2.647959000	7.738735000	1.373035000
C	-3.035144000	4.626477000	2.655787000
C	2.227068000	5.260490000	1.614589000
C	-3.589767000	2.260005000	2.490809000
C	-2.457832000	-2.725444000	1.707207000
C	1.726512000	6.655763000	1.956700000
C	3.144640000	3.092694000	2.320622000
C	-1.080302000	-3.255568000	2.111403000
C	3.080598000	-1.792018000	3.210830000
C	2.660136000	4.379661000	2.614925000
C	-3.559474000	-3.487305000	2.467322000
C	-2.446454000	4.440450000	3.911292000
C	-3.008268000	2.051894000	3.773575000
C	4.480789000	-1.701888000	3.845297000
C	2.049003000	-2.116949000	4.296197000
C	3.610527000	2.201982000	3.468811000
C	-1.861105000	5.611180000	4.685459000
C	-2.438773000	3.142981000	4.444202000
C	-3.044314000	0.690536000	4.465587000
C	4.950412000	2.691255000	4.041977000
C	-2.733322000	5.957743000	5.903435000
C	2.553728000	2.070136000	4.576364000
C	-4.365814000	0.494974000	5.234394000
C	-0.403654000	5.362645000	5.102006000
C	-1.875986000	0.454836000	5.435087000
K	-0.233013000	3.073560000	1.754845000
Ge	1.553427000	-0.155296000	-0.165599000
Ge	-1.948636000	0.599928000	0.020928000
Cl	-0.057773000	0.131928000	2.580773000
O	-0.433458000	-0.380784000	-2.355380000
H	-0.202113000	-1.967184000	-5.949895000
H	-1.905773000	-1.419739000	-5.889922000
H	0.391369000	0.228840000	-7.098886000
H	-1.291629000	0.757842000	-6.992035000
H	-1.089317000	-1.955394000	-4.401879000
H	2.238725000	0.355492000	-5.967471000
H	-2.595386000	1.874794000	-5.665732000
H	0.003874000	1.793021000	-6.331880000
H	4.252644000	0.308375000	-4.525861000
H	-5.678028000	-0.296318000	-4.243078000
H	-4.277097000	2.704031000	-4.047693000

H	3.969512000	-3.049422000	-3.740876000
H	-4.992555000	-1.905348000	-4.534122000
H	3.287830000	-4.455636000	-2.891745000
H	-3.937499000	-0.572785000	-4.008487000
H	2.516780000	-2.856893000	-2.735154000
H	6.237254000	-3.519855000	-2.650229000
H	-7.346606000	-1.651200000	-2.805355000
H	1.536848000	2.833941000	-3.234711000
H	-6.391827000	-3.153813000	-2.895376000
H	3.988341000	3.629861000	-3.308309000
H	5.586106000	-4.874796000	-1.693120000
H	-3.551558000	-3.164126000	-3.571431000
H	0.199521000	-3.367658000	-3.100823000
H	-1.822346000	-4.613189000	-3.871468000
H	4.046836000	0.059029000	-2.077416000
H	0.592595000	-5.066543000	-3.467870000
H	4.585830000	-1.973362000	-1.634461000
H	-5.368228000	-0.444043000	-1.860326000
H	-3.745099000	4.679869000	-1.367259000
H	1.103187000	4.267976000	-2.272396000
H	-4.150216000	2.074076000	-1.658849000
H	3.224923000	-5.360481000	-0.869627000
H	3.642144000	5.048105000	-2.284951000
H	6.394634000	-3.518549000	-0.873470000
H	0.722124000	2.629230000	-1.670200000
H	-6.797605000	-2.454468000	-1.314254000
H	3.620300000	-7.493968000	0.306991000
H	-6.035829000	4.798968000	-0.221955000
H	3.164577000	2.109230000	-1.558927000
H	4.889423000	3.875491000	-1.788628000
H	0.438903000	-4.529650000	-1.780599000
H	-1.166685000	-6.898379000	-3.113415000
H	-2.369534000	3.931288000	-0.535822000
H	2.780381000	-8.461151000	1.543958000
H	-2.970673000	5.533639000	-0.013242000
H	1.258678000	-6.747576000	-0.626274000
H	-4.517039000	2.877029000	0.144883000
H	-2.798338000	-6.470864000	-2.528603000
H	3.980073000	-7.232591000	2.031869000
H	-5.354326000	5.637034000	1.195781000
H	1.933177000	5.473525000	-0.513476000
H	0.495184000	-7.738141000	0.639573000
H	-6.255100000	4.114699000	1.410851000
H	6.245129000	-1.763495000	0.690683000
H	-1.409137000	-6.419819000	-1.415956000
H	0.168364000	6.789323000	0.415803000
H	-6.264714000	-1.999180000	0.570921000
H	7.281020000	0.504923000	0.625712000
H	-1.239527000	-4.274901000	-0.152081000
H	2.649418000	7.708098000	0.270868000
H	0.097916000	-6.006141000	0.497903000
H	1.644505000	-6.424545000	2.410255000
H	5.820528000	2.516389000	0.760013000

H	-7.411641000	-0.304246000	1.982628000
H	-3.061333000	5.633863000	2.230944000
H	-0.410690000	6.134818000	1.969122000
H	-0.085893000	7.868857000	1.807030000
H	2.131141000	-4.341924000	3.142184000
H	-0.271569000	-2.758013000	1.557354000
H	-6.134145000	1.658458000	2.825171000
H	2.312079000	8.742524000	1.680916000
H	-2.504134000	-1.674795000	2.027301000
H	3.685958000	7.603714000	1.716084000
H	2.828995000	-0.796168000	2.819330000
H	-0.991305000	-4.341807000	1.948406000
H	5.244564000	-1.389218000	3.120703000
H	-4.559553000	-3.082996000	2.254529000
H	1.040864000	-2.238085000	3.878834000
H	-3.557541000	-4.553728000	2.184725000
H	1.756551000	6.748964000	3.055570000
H	3.775359000	1.195997000	3.061503000
H	-1.872981000	6.482575000	4.008495000
H	-0.914777000	-3.075231000	3.184411000
H	-2.992016000	-0.077565000	3.678663000
H	4.781740000	-2.680473000	4.256384000
H	5.727874000	2.731785000	3.264209000
H	-3.770416000	6.173197000	5.601116000
H	2.630745000	4.712394000	3.655914000
H	2.308458000	-3.034965000	4.849024000
H	-3.392404000	-3.424535000	3.555925000
H	4.480766000	-0.972144000	4.672379000
H	1.600456000	1.692770000	4.175725000
H	-5.243327000	0.553048000	4.578795000
H	2.012924000	-1.294247000	5.028098000
H	0.234483000	5.131065000	4.234680000
H	-0.905126000	0.713814000	4.992166000
H	4.851611000	3.700355000	4.476341000
H	-1.993632000	2.981732000	5.426821000
H	-2.337206000	6.842689000	6.428249000
H	5.299658000	2.013021000	4.837864000
H	-2.757521000	5.121637000	6.622013000
H	2.362122000	3.029727000	5.083737000
H	0.018669000	6.253946000	5.593147000
H	2.895919000	1.355252000	5.341984000
H	-4.471369000	1.265178000	6.017035000
H	-4.375822000	-0.491399000	5.726977000
H	-1.843615000	-0.607185000	5.725487000
H	-0.321892000	4.521914000	5.809772000
H	-1.994807000	1.040090000	6.361705000
B	-0.167213000	0.130137000	0.723453000

*

K[1a$^{\cdot}$]_ωB97X_RIJCOSX_CPCM(Et_2O) (spin density)

* xyz 0 2			
C	-0.885675000	-1.362439000	-5.458643000
C	-0.383717000	0.842061000	-6.532743000
C	-0.484078000	0.106012000	-5.196410000
C	2.104581000	0.349991000	-4.998453000
C	-2.599528000	1.543456000	-4.684387000
C	0.845273000	0.128075000	-4.434058000
C	-1.538619000	0.731396000	-4.275221000
C	3.247536000	0.351551000	-4.192683000
C	-3.554071000	1.975669000	-3.759502000
C	-4.987737000	-1.160460000	-3.787992000
C	3.368919000	-3.284614000	-2.858400000
C	0.799355000	-0.088412000	-3.055048000
C	-1.463979000	0.426425000	-2.912482000
C	3.145810000	0.177059000	-2.812836000
C	-3.450657000	1.623848000	-2.413692000
C	-6.595299000	-2.218523000	-2.197019000
C	5.676524000	-3.592168000	-1.886611000
C	-5.260778000	-1.468492000	-2.313240000
C	4.303198000	-2.938002000	-1.695910000
C	1.900050000	-0.023333000	-2.196888000
C	1.704569000	3.438709000	-2.299757000
C	-2.373908000	0.855675000	-1.944911000
C	4.118781000	4.148289000	-2.039546000
C	-0.204729000	-4.629176000	-2.785394000
C	-3.459766000	-3.224777000	-2.392665000
C	-1.686820000	-5.011922000	-2.703195000
C	-4.137294000	-2.253077000	-1.641759000
C	2.982661000	3.292021000	-1.470451000
C	3.247443000	-4.609195000	-0.148549000
C	-3.252725000	4.509511000	-0.505018000
C	3.713197000	-3.303018000	-0.339532000
C	-2.458298000	-4.021746000	-1.846993000
C	3.371005000	-7.492151000	1.020919000
C	-5.459210000	4.593257000	0.728197000
C	-1.861245000	-6.453112000	-2.216120000
C	-4.161155000	3.808581000	0.508184000
C	-3.778112000	-2.060451000	-0.290449000
C	2.745562000	-5.045388000	1.076648000
C	2.257220000	-6.471683000	1.271516000
C	1.030426000	-6.770805000	0.406131000
C	3.662241000	-2.387028000	0.732804000
C	2.764167000	3.591982000	0.002423000
C	-2.173119000	-3.870022000	-0.487381000
C	4.214756000	-0.986442000	0.612870000
C	-4.393749000	-0.962567000	0.544397000
C	5.602884000	-0.829353000	0.724249000
C	3.377208000	0.152433000	0.511486000
C	2.194267000	4.821859000	0.356048000
C	6.175962000	0.437193000	0.774646000
C	-5.690728000	-1.137989000	1.040917000

C	-3.654508000	0.198867000	0.893120000
C	-2.813582000	-2.916354000	0.304389000
C	3.955319000	1.429689000	0.722623000
C	5.349074000	1.555250000	0.818387000
C	2.716176000	-4.129541000	2.129982000
C	3.179223000	2.696890000	1.013686000
C	-6.283304000	-0.175343000	1.852508000
C	-3.464933000	3.543455000	1.835605000
C	3.166022000	-2.812813000	1.992213000
C	-4.219087000	1.109604000	1.817801000
C	0.233457000	6.957036000	1.276610000
C	-5.543017000	0.933294000	2.246446000
C	2.580925000	7.658713000	1.876648000
C	-2.858605000	4.619999000	2.497351000
C	2.041461000	5.216421000	1.687874000
C	-3.471445000	2.267066000	2.441921000
C	-2.509119000	-2.856838000	1.799613000
C	1.498371000	6.588640000	2.055542000
C	3.011737000	3.067813000	2.371814000
C	-1.130776000	-3.395763000	2.180568000
C	3.174479000	-1.896721000	3.216434000
C	2.455919000	4.319519000	2.676480000
C	-3.594341000	-3.597972000	2.592907000
C	-2.254356000	4.480219000	3.749200000
C	-2.874499000	2.105569000	3.720425000
C	4.553115000	-1.889525000	3.890538000
C	2.101397000	-2.230539000	4.254326000
C	3.476863000	2.185229000	3.528560000
C	-1.688867000	5.697828000	4.465814000
C	-2.268074000	3.210591000	4.333985000
C	-2.939205000	0.788919000	4.494997000
C	4.767459000	2.735614000	4.146244000
C	-2.793131000	6.393889000	5.270757000
C	2.401854000	1.996987000	4.603572000
C	-4.214635000	0.718287000	5.348221000
C	-0.485392000	5.385837000	5.355273000
C	-1.734045000	0.545157000	5.410224000
K	-0.191613000	3.003241000	1.722105000
Ge	1.570089000	-0.075883000	-0.269190000
Ge	-1.948503000	0.553595000	-0.052871000
Cl	-0.051248000	0.010618000	2.475637000
O	-0.412922000	-0.332334000	-2.455633000
H	-0.123368000	-1.863188000	-6.077027000
H	-1.850877000	-1.404134000	-5.988833000
H	0.363065000	0.364029000	-7.183936000
H	-1.344174000	0.806340000	-7.068115000
H	-0.989376000	-1.924385000	-4.516703000
H	2.204637000	0.523955000	-6.072818000
H	-2.700194000	1.830910000	-5.734039000
H	-0.100695000	1.897719000	-6.395994000
H	4.228777000	0.515079000	-4.647848000
H	-5.710960000	-0.416886000	-4.159249000
H	-4.391975000	2.592956000	-4.096623000

H	3.784090000	-2.906145000	-3.806719000
H	-5.097693000	-2.060728000	-4.414533000
H	3.237377000	-4.374197000	-2.965136000
H	-3.976800000	-0.756683000	-3.942537000
H	2.374089000	-2.831281000	-2.722502000
H	6.119630000	-3.293819000	-2.850704000
H	-7.405922000	-1.641436000	-2.671341000
H	1.876652000	3.079153000	-3.327599000
H	-6.530594000	-3.193038000	-2.709406000
H	4.312316000	3.877898000	-3.090322000
H	5.593564000	-4.691925000	-1.879382000
H	-3.712765000	-3.357684000	-3.448978000
H	-0.076483000	-3.604380000	-3.171020000
H	-2.106799000	-4.948921000	-3.722496000
H	4.052993000	0.235085000	-2.202396000
H	0.342580000	-5.316658000	-3.451451000
H	4.452721000	-1.849364000	-1.710880000
H	-5.359527000	-0.507870000	-1.781665000
H	-3.769034000	4.621228000	-1.471494000
H	1.375079000	4.489143000	-2.363230000
H	-4.214443000	1.975878000	-1.714004000
H	3.289791000	-5.310966000	-0.988964000
H	3.866355000	5.221460000	-2.007318000
H	6.376124000	-3.303475000	-1.086143000
H	0.880186000	2.842116000	-1.874172000
H	-6.881496000	-2.408905000	-1.153171000
H	3.708894000	-7.460952000	-0.028189000
H	-5.991517000	4.736388000	-0.225721000
H	3.302142000	2.246405000	-1.543973000
H	5.052261000	4.002400000	-1.472636000
H	0.273177000	-4.672001000	-1.791822000
H	-1.337780000	-7.155907000	-2.884498000
H	-2.334864000	3.925859000	-0.691890000
H	3.015763000	-8.514728000	1.226693000
H	-2.962082000	5.517873000	-0.167188000
H	1.274369000	-6.702027000	-0.667446000
H	-4.440351000	2.832429000	0.086644000
H	-2.924340000	-6.739081000	-2.183930000
H	4.244365000	-7.297355000	1.662916000
H	-5.254052000	5.589581000	1.153994000
H	1.891997000	5.505751000	-0.443805000
H	0.650527000	-7.787223000	0.599436000
H	-6.133229000	4.062124000	1.418935000
H	6.237615000	-1.719592000	0.783026000
H	-1.444949000	-6.583854000	-1.203535000
H	0.436836000	7.068038000	0.199371000
H	-6.242469000	-2.048347000	0.790365000
H	7.262139000	0.551486000	0.832955000
H	-1.411922000	-4.511466000	-0.036222000
H	2.884288000	7.732666000	0.819127000
H	0.216122000	-6.057138000	0.611696000
H	1.950401000	-6.562043000	2.328101000
H	5.786193000	2.550790000	0.948276000

H	-7.310888000	-0.305551000	2.203693000
H	-2.881591000	5.612247000	2.031911000
H	-0.559406000	6.198096000	1.392164000
H	-0.173932000	7.915177000	1.635680000
H	2.332839000	-4.466128000	3.097717000
H	-0.327321000	-2.920651000	1.595021000
H	-5.990298000	1.676142000	2.914360000
H	2.209511000	8.646355000	2.193291000
H	-2.534806000	-1.798530000	2.103191000
H	3.477484000	7.422477000	2.470311000
H	2.968591000	-0.875803000	2.859649000
H	-1.062007000	-4.487654000	2.041039000
H	5.351014000	-1.579867000	3.200116000
H	-4.597101000	-3.187369000	2.401404000
H	1.105561000	-2.326030000	3.797644000
H	-3.608431000	-4.666747000	2.320572000
H	1.236948000	6.553506000	3.128100000
H	3.703541000	1.189215000	3.120714000
H	-1.356731000	6.402804000	3.682768000
H	-0.937266000	-3.193612000	3.246230000
H	-2.974102000	-0.027449000	3.755094000
H	4.801763000	-2.895558000	4.268536000
H	5.568420000	2.823985000	3.396378000
H	-3.641986000	6.674029000	4.627991000
H	2.360717000	4.616259000	3.726852000
H	2.327064000	-3.165331000	4.793348000
H	-3.398902000	-3.526728000	3.675578000
H	4.558365000	-1.195629000	4.748059000
H	1.475338000	1.577313000	4.179396000
H	-5.130073000	0.786686000	4.746428000
H	2.051604000	-1.425959000	5.006292000
H	0.302745000	4.843076000	4.807123000
H	-0.775754000	0.730692000	4.905011000
H	4.600587000	3.734081000	4.584009000
H	-1.814398000	3.077143000	5.319031000
H	-2.409309000	7.308351000	5.750620000
H	5.124233000	2.070323000	4.948936000
H	-3.173062000	5.726764000	6.062238000
H	2.154958000	2.942709000	5.114086000
H	-0.045437000	6.318438000	5.741628000
H	2.758641000	1.294112000	5.373706000
H	-4.227588000	1.539794000	6.084095000
H	-4.246225000	-0.232397000	5.904462000
H	-1.737429000	-0.500650000	5.755475000
H	-0.769927000	4.773169000	6.225843000
H	-1.774366000	1.181303000	6.309517000
B	-0.160599000	0.071880000	0.620759000

*

K[1a$^{\cdot}$]_BP86_RI_D4_CPCM(Et_2O) (spin density)

```
* xyz 0 2
C     -0.963828000    -1.437849000    -5.330330000
C     -0.315036000     0.736287000    -6.429098000
C     -0.467811000     0.015135000    -5.082489000
C      2.139613000     0.137937000    -4.863965000
C     -2.506584000     1.587232000    -4.587642000
C      0.856831000    -0.038303000    -4.307716000
C     -1.488506000     0.708862000    -4.167624000
C      3.282599000     0.101289000    -4.041547000
C     -3.463136000     2.060789000    -3.668977000
C     -4.979684000    -0.998097000    -3.894902000
C      3.597593000    -3.425785000    -2.804872000
C      0.791333000    -0.255782000    -2.921796000
C     -1.447734000     0.395692000    -2.797406000
C      3.163879000    -0.067455000    -2.653399000
C     -3.400923000     1.692098000    -2.314813000
C     -6.514966000    -2.262682000    -2.361663000
C      5.792388000    -3.856530000    -1.608279000
C     -5.245335000    -1.384967000    -2.430439000
C      4.449729000    -3.098941000    -1.568139000
C      1.895124000    -0.230856000    -2.051345000
C      1.314173000     3.113900000    -2.183218000
C     -2.353640000     0.875088000    -1.838673000
C      3.734447000     3.902612000    -2.356251000
C      0.125878000    -4.274940000    -2.769280000
C     -3.289739000    -2.980112000    -2.543415000
C     -1.370782000    -4.614333000    -2.879088000
C     -4.065902000    -2.093718000    -1.770560000
C      2.714867000     3.081953000    -1.545984000
C      3.153292000    -4.642008000    -0.044541000
C     -3.168960000     4.507849000    -0.340701000
C      3.720881000    -3.370727000    -0.255747000
C     -2.216873000    -3.701807000    -2.003628000
C      3.063501000    -7.448870000     1.401538000
C     -5.456589000     4.736121000     0.764982000
C     -1.642457000    -6.099135000    -2.566528000
C     -4.189964000     3.872100000     0.620500000
C     -3.737944000    -1.911630000    -0.399328000
C      2.532636000    -4.987666000     1.166632000
C      1.954745000    -6.378095000     1.396793000
C      0.851189000    -6.720942000     0.381158000
C      3.639954000    -2.396864000     0.776058000
C      2.684705000     3.511916000    -0.088362000
C     -1.956748000    -3.566002000    -0.627770000
C      4.230494000    -1.027810000     0.615551000
C     -4.431291000    -0.882440000     0.445390000
C      5.631690000    -0.862328000     0.622092000
C      3.379247000     0.109719000     0.557858000
C      2.181022000     4.786699000     0.242963000
C      6.195651000     0.420030000     0.587022000
C     -5.774801000    -1.050390000     0.836688000
```

C	-3.700330000	0.249940000	0.912631000
C	-2.696158000	-2.696090000	0.188312000
C	3.952663000	1.404773000	0.664625000
C	5.359405000	1.543708000	0.648238000
C	2.495655000	-4.021547000	2.187921000
C	3.182244000	2.668418000	0.942959000
C	-6.409953000	-0.102122000	1.650833000
C	-3.569340000	3.568442000	1.978917000
C	3.039382000	-2.735591000	2.029018000
C	-4.311360000	1.143629000	1.827962000
C	0.189452000	6.854017000	1.483793000
C	-5.676428000	0.980194000	2.154228000
C	2.562053000	7.742744000	1.264672000
C	-2.986932000	4.621729000	2.719223000
C	2.165117000	5.262975000	1.570135000
C	-3.573636000	2.254664000	2.520130000
C	-2.430455000	-2.655761000	1.692972000
C	1.661657000	6.664115000	1.897380000
C	3.119404000	3.105058000	2.299268000
C	-1.054496000	-3.194668000	2.097847000
C	3.048909000	-1.757545000	3.206363000
C	2.626121000	4.398510000	2.582571000
C	-3.545907000	-3.402399000	2.454540000
C	-2.389363000	4.404935000	3.974273000
C	-2.978736000	2.012620000	3.798346000
C	4.459788000	-1.647675000	3.822144000
C	2.029431000	-2.082097000	4.306136000
C	3.605158000	2.224566000	3.449460000
C	-1.786103000	5.556369000	4.769278000
C	-2.385785000	3.090187000	4.484515000
C	-3.024454000	0.632260000	4.456025000
C	4.958111000	2.718993000	3.996260000
C	-2.628247000	5.862088000	6.023639000
C	2.560695000	2.103953000	4.573972000
C	-4.370130000	0.405524000	5.180021000
C	-0.313729000	5.294643000	5.135419000
C	-1.879007000	0.378878000	5.451073000
K	-0.244482000	3.073349000	1.752934000
Ge	1.548347000	-0.197555000	-0.129402000
Ge	-1.949662000	0.575474000	0.051733000
Cl	-0.042134000	0.169108000	2.617200000
O	-0.438133000	-0.412509000	-2.306287000
H	-0.220780000	-2.003191000	-5.929435000
H	-1.928190000	-1.425434000	-5.878147000
H	0.406387000	0.204055000	-7.079890000
H	-1.280397000	0.760194000	-6.972079000
H	-1.121997000	-1.974983000	-4.375534000
H	2.253726000	0.309564000	-5.943830000
H	-2.575957000	1.884560000	-5.643917000
H	0.037465000	1.779318000	-6.297613000
H	4.278991000	0.235131000	-4.490669000
H	-5.770918000	-0.312156000	-4.261018000
H	-4.272391000	2.720007000	-4.019343000

H	4.134518000	-3.134801000	-3.731161000
H	-4.988731000	-1.887831000	-4.558913000
H	3.373735000	-4.510597000	-2.875606000
H	-4.006218000	-0.490290000	-4.015661000
H	2.637681000	-2.876305000	-2.785075000
H	6.352869000	-3.615878000	-2.536094000
H	-7.384374000	-1.731418000	-2.803272000
H	1.346532000	2.697247000	-3.210011000
H	-6.363803000	-3.201644000	-2.935350000
H	3.790175000	3.526584000	-3.399020000
H	5.630258000	-4.955076000	-1.586076000
H	-3.517816000	-3.103115000	-3.613013000
H	0.316470000	-3.210957000	-3.015428000
H	-1.683890000	-4.431545000	-3.931050000
H	4.065840000	-0.026933000	-2.025068000
H	0.725369000	-4.899489000	-3.463344000
H	4.678308000	-2.016146000	-1.602551000
H	-5.436178000	-0.452713000	-1.857824000
H	-3.620554000	4.681825000	-1.338140000
H	0.912906000	4.147383000	-2.249285000
H	-4.157867000	2.072718000	-1.613132000
H	3.211751000	-5.388642000	-0.852469000
H	3.454913000	4.976810000	-2.396962000
H	6.435187000	-3.597007000	-0.742574000
H	0.599095000	2.498534000	-1.597401000
H	-6.779722000	-2.547581000	-1.325481000
H	3.562716000	-7.509922000	0.411472000
H	-5.931462000	4.902704000	-0.224087000
H	3.057903000	2.032264000	-1.566313000
H	4.748763000	3.832355000	-1.912435000
H	0.513938000	-4.451272000	-1.745328000
H	-1.046781000	-6.760763000	-3.230016000
H	-2.297791000	3.834446000	-0.492603000
H	2.645231000	-8.451548000	1.630077000
H	-2.799703000	5.484772000	0.035515000
H	1.249654000	-6.756668000	-0.653989000
H	-4.501924000	2.901667000	0.184055000
H	-2.714636000	-6.348225000	-2.703755000
H	3.841474000	-7.219338000	2.158187000
H	-5.220973000	5.730601000	1.199256000
H	1.812792000	5.435477000	-0.568051000
H	0.402478000	-7.712021000	0.601272000
H	-6.200746000	4.245669000	1.424992000
H	6.279987000	-1.751029000	0.663431000
H	-1.371685000	-6.339908000	-1.518056000
H	0.057070000	6.742410000	0.387328000
H	-6.327194000	-1.940366000	0.502470000
H	7.289056000	0.545594000	0.558446000
H	-1.139427000	-4.149046000	-0.181815000
H	2.534643000	7.686793000	0.156249000
H	0.039642000	-5.966241000	0.402380000
H	1.490916000	-6.368593000	2.407831000
H	5.795916000	2.553676000	0.695517000

H	-7.470924000	-0.223663000	1.918472000
H	-3.004762000	5.645105000	2.309384000
H	-0.477312000	6.120017000	1.982914000
H	-0.173930000	7.863817000	1.763871000
H	2.028076000	-4.296057000	3.144975000
H	-0.237036000	-2.683421000	1.551290000
H	-6.159079000	1.712310000	2.820101000
H	2.225975000	8.758353000	1.559541000
H	-2.468429000	-1.592475000	2.009962000
H	3.617001000	7.622095000	1.584174000
H	2.782728000	-0.759405000	2.799779000
H	-0.962534000	-4.285813000	1.916132000
H	5.214437000	-1.324869000	3.079990000
H	-4.547375000	-2.983243000	2.234627000
H	1.008203000	-2.201927000	3.898363000
H	-3.557866000	-4.477475000	2.175700000
H	1.720438000	6.779487000	3.001599000
H	3.763122000	1.207489000	3.039371000
H	-1.819163000	6.453902000	4.113500000
H	-0.891177000	-3.031284000	3.181751000
H	-2.944753000	-0.117728000	3.640227000
H	4.782550000	-2.627545000	4.233804000
H	5.728753000	2.746867000	3.200504000
H	-3.681534000	6.082643000	5.756080000
H	2.614052000	4.752109000	3.626437000
H	2.295577000	-3.008243000	4.858148000
H	-3.381425000	-3.335461000	3.550581000
H	4.463763000	-0.911246000	4.652992000
H	1.596300000	1.717311000	4.186001000
H	-5.232489000	0.473971000	4.492222000
H	2.002019000	-1.256154000	5.045700000
H	0.303124000	5.097737000	4.234568000
H	-0.889357000	0.639137000	5.029975000
H	4.868294000	3.741707000	4.420071000
H	-1.924962000	2.903150000	5.465378000
H	-2.219859000	6.737922000	6.569320000
H	5.321873000	2.047797000	4.801735000
H	-2.629131000	4.998541000	6.721468000
H	2.370623000	3.076262000	5.074451000
H	0.127237000	6.170432000	5.653971000
H	2.914328000	1.396035000	5.351416000
H	-4.509155000	1.159515000	5.983548000
H	-4.387969000	-0.600094000	5.649233000
H	-1.857312000	-0.692945000	5.734054000
H	-0.209978000	4.419090000	5.809249000
H	-2.016097000	0.959302000	6.387619000
B	-0.164261000	0.135073000	0.766373000

*

K[1a·]_BP86_RI_D3BJ (frontier orbitals)

* xyz 0 2

C	0.045098000	-1.522035000	-4.526685000
C	1.712049000	-5.496491000	-5.063465000
C	-1.433497000	-1.618268000	-4.940047000
C	-1.735174000	-2.945370000	-5.660754000
C	-0.776392000	2.420858000	-5.002135000
C	1.541992000	-5.634092000	-3.545343000
C	3.595257000	-0.737546000	-4.463448000
C	-4.835484000	2.099911000	-3.667934000
C	0.049640000	-5.751117000	-3.181093000
C	-3.334146000	-0.408527000	-3.758860000
C	-2.354150000	-1.408219000	-3.748471000
C	5.701850000	-2.018624000	-3.866363000
C	-6.548736000	0.311207000	-3.243920000
C	2.936506000	-3.482270000	-3.346219000
C	4.386166000	-1.417341000	-3.334381000
C	-0.257624000	3.352155000	-3.870013000
C	2.226190000	-4.527740000	-2.743219000
C	-5.224526000	0.931812000	-2.748192000
C	2.338696000	3.204630000	-3.536479000
C	-0.034581000	4.762694000	-4.428554000
C	-4.147756000	-0.141375000	-2.641368000
C	1.029103000	2.749962000	-3.290664000
C	-2.255201000	4.326563000	-2.474791000
C	-1.293906000	3.332578000	-2.738378000
C	-2.243555000	-2.210790000	-2.600861000
C	3.576605000	-2.476496000	-2.595317000
C	3.435786000	2.577839000	-2.915058000
C	-3.230170000	4.126479000	-1.478866000
C	0.888837000	1.660373000	-2.416442000
C	-1.323383000	2.189059000	-1.922841000
C	2.174549000	-4.553597000	-1.334302000
C	3.244427000	1.513419000	-2.021962000
C	-3.948020000	-0.895114000	-1.454041000
C	-3.018273000	-1.981844000	-1.455734000
C	1.945807000	1.034289000	-1.737096000
C	-3.236643000	2.953400000	-0.707870000
C	3.476562000	-2.500238000	-1.182834000
C	-2.240781000	1.970960000	-0.886607000
C	2.778871000	-3.571624000	-0.538720000
C	-4.585012000	-0.507709000	-0.152202000
C	4.113341000	-1.449013000	-0.326444000
C	-1.643743000	-3.799282000	-0.283336000
C	5.514558000	-1.384497000	-0.185854000
C	-5.965371000	-0.628714000	0.095008000
C	-2.888515000	-2.909590000	-0.248930000
C	-4.158969000	-3.765821000	-0.079154000
C	3.283418000	-0.565667000	0.414597000
C	-3.729379000	-0.045176000	0.889779000
C	2.736617000	-3.681423000	0.986420000
C	6.095711000	-0.460740000	0.693027000

C	1.366849000	3.524242000	0.448964000
C	-6.501261000	-0.284879000	1.345118000
C	1.647159000	-4.616672000	1.522684000
C	4.109545000	-4.091261000	1.555994000
C	2.759429000	3.055678000	0.908006000
C	3.794303000	4.191911000	0.866141000
C	3.866642000	0.271755000	1.397626000
C	5.274516000	0.333334000	1.505973000
C	-4.254458000	0.191779000	2.179875000
C	-2.422983000	3.834423000	2.431802000
C	-5.648889000	0.102597000	2.387414000
C	-3.624863000	3.014352000	2.936517000
C	-4.647730000	3.888418000	3.681424000
C	2.700509000	2.385846000	2.269705000
C	3.103437000	1.035623000	2.446234000
C	-3.388666000	0.493302000	3.369165000
C	-3.163540000	1.834294000	3.781415000
C	2.236928000	3.120841000	3.380915000
C	-3.132164000	-2.041195000	3.777544000
C	2.950987000	0.419446000	3.723626000
C	-2.882565000	-0.582891000	4.160742000
C	3.300963000	-1.050167000	3.942966000
C	-2.104245000	-3.022072000	4.363018000
C	-4.549321000	-2.495905000	4.187592000
C	-2.476337000	2.080988000	4.986587000
C	4.694247000	-1.202767000	4.581775000
C	2.236481000	-1.795738000	4.765212000
C	2.158776000	2.558236000	4.668378000
C	2.497030000	1.198068000	4.807727000
C	-2.173136000	-0.282076000	5.340919000
C	-1.976349000	1.037946000	5.787848000
C	0.652807000	4.397207000	5.606722000
C	1.771734000	3.382957000	5.888484000
C	3.017480000	4.076518000	6.476268000
C	-1.316786000	1.351375000	7.124537000
C	-0.235205000	0.340038000	7.529235000
C	-2.391172000	1.485325000	8.223538000
K	-0.133207000	1.135186000	3.181350000
Ge	1.489343000	-0.255716000	-0.347917000
Ge	-1.918477000	0.479404000	0.322744000
Cl	-0.157866000	-1.649942000	1.994958000
O	-0.373734000	1.199279000	-2.090487000
H	0.710196000	-1.601900000	-5.410417000
H	1.228068000	-6.346532000	-5.585210000
H	-1.083919000	-3.072941000	-6.550771000
H	2.778730000	-5.480247000	-5.364213000
H	-1.641586000	-0.793257000	-5.657317000
H	0.258676000	-0.561898000	-4.017631000
H	1.237810000	-4.564508000	-5.435798000
H	-0.025021000	2.343639000	-5.814140000
H	-1.722109000	2.818938000	-5.422726000
H	3.363223000	-1.445198000	-5.286476000
H	-4.873138000	1.804935000	-4.737498000

H	-2.791690000	-2.993546000	-5.993296000
H	-0.977565000	1.403125000	-4.616452000
H	0.332255000	-2.328318000	-3.821707000
H	-3.449354000	0.202915000	-4.665916000
H	-0.429012000	-6.588135000	-3.729665000
H	5.501126000	-2.815522000	-4.612930000
H	4.181036000	0.098129000	-4.897190000
H	-1.556355000	-3.809879000	-4.989098000
H	-6.420507000	-0.091240000	-4.270545000
H	3.006159000	-3.434435000	-4.441167000
H	6.322286000	-1.240846000	-4.358799000
H	-5.542041000	2.944743000	-3.539617000
H	-0.489114000	-4.818510000	-3.445843000
H	2.642482000	-0.320923000	-4.088040000
H	-3.818404000	2.471230000	-3.450711000
H	-7.357260000	1.071531000	-3.272940000
H	2.509445000	4.055607000	-4.210643000
H	2.029224000	-6.589499000	-3.238477000
H	0.701388000	4.745718000	-5.256397000
H	-2.267565000	5.251483000	-3.068395000
H	-0.975831000	5.172735000	-4.844633000
H	6.301950000	-2.473567000	-3.052551000
H	-0.102326000	-5.925026000	-2.098627000
H	-6.887656000	-0.527457000	-2.606109000
H	4.454854000	2.942049000	-3.115961000
H	-1.501263000	-3.018977000	-2.587384000
H	4.651641000	-0.632421000	-2.600733000
H	-3.999851000	4.895166000	-1.311087000
H	-5.389861000	1.338503000	-1.728356000
H	0.334265000	5.457838000	-3.647949000
H	1.645275000	-5.381232000	-0.841757000
H	4.111521000	1.078336000	-1.504223000
H	-4.007158000	2.810279000	0.064297000
H	-1.706696000	-4.560119000	-1.087232000
H	-6.626630000	-1.003498000	-0.698768000
H	6.146596000	-2.070748000	-0.769222000
H	-0.720605000	-3.206664000	-0.438973000
H	-4.307904000	-4.421994000	-0.962056000
H	1.410601000	3.912841000	-0.587569000
H	-5.064481000	-3.139663000	0.036782000
H	-2.800098000	-2.268387000	0.653154000
H	1.861662000	-5.681288000	1.290557000
H	3.867411000	4.600936000	-0.162275000
H	4.406056000	-5.091503000	1.176468000
H	0.652810000	-4.361752000	1.113590000
H	3.094507000	2.289120000	0.188005000
H	-1.543874000	-4.341000000	0.678015000
H	0.645367000	2.680083000	0.448992000
H	7.190720000	-0.386923000	0.776215000
H	4.904884000	-3.372939000	1.281751000
H	-7.586442000	-0.350906000	1.516659000
H	2.503101000	-2.666703000	1.372097000
H	-2.752897000	4.666750000	1.779463000

H	-4.075714000	-4.413305000	0.818773000
H	-4.133257000	2.590937000	2.047922000
H	0.963538000	4.330780000	1.097484000
H	-1.747641000	3.201267000	1.817404000
H	-5.017308000	4.702865000	3.025185000
H	4.799121000	3.828738000	1.163129000
H	1.587270000	-4.527114000	2.626220000
H	4.069017000	-4.145670000	2.664116000
H	3.520195000	5.026902000	1.545340000
H	5.722115000	1.017459000	2.243600000
H	-5.520511000	3.289170000	4.010226000
H	-1.842594000	4.272099000	3.271589000
H	-6.058941000	0.335634000	3.382324000
H	-3.064198000	-2.094484000	2.670485000
H	3.336785000	-1.523871000	2.941967000
H	-4.203780000	4.361352000	4.582523000
H	1.948834000	4.171882000	3.228779000
H	-2.217128000	-4.014217000	3.882697000
H	-5.337133000	-1.898947000	3.694340000
H	-1.062783000	-2.687475000	4.199128000
H	5.478310000	-0.738926000	3.953241000
H	-4.704467000	-3.557786000	3.906752000
H	1.228359000	-1.678132000	4.320379000
H	2.464114000	-2.880550000	4.789511000
H	4.949184000	-2.275055000	4.707520000
H	-2.332093000	3.121851000	5.318379000
H	0.989502000	5.202104000	4.921753000
H	-2.253582000	-3.169893000	5.453408000
H	-4.682716000	-2.409405000	5.286467000
H	-0.229830000	3.911743000	5.143050000
H	4.726546000	-0.723035000	5.582851000
H	3.445342000	4.794249000	5.745738000
H	2.198772000	-1.447246000	5.818411000
H	-1.780600000	-1.112195000	5.942431000
H	2.418659000	0.734264000	5.803255000
H	0.318481000	4.884814000	6.544578000
H	3.807179000	3.339495000	6.723875000
H	-0.830147000	2.345474000	7.015141000
H	1.397138000	2.666457000	6.651993000
H	0.502051000	0.184597000	6.716605000
H	2.763674000	4.636647000	7.400036000
H	-3.146731000	2.250883000	7.956543000
H	-0.670161000	-0.649849000	7.777474000
H	-2.924458000	0.522206000	8.364365000
H	0.311216000	0.690061000	8.428129000
H	-1.935406000	1.771526000	9.194045000
B	-0.219491000	-0.460132000	0.579324000

*

K_2[1a]_BP86_RI_D3BJ (frontier orbitals)

```
* xyz 0 1
C    -4.089388000   -0.689042000   -5.354448000
C    -2.146853000    2.865345000   -4.728031000
C    -1.713122000   -1.491137000   -4.935841000
C    -2.987620000   -0.912212000   -4.300479000
C     0.262028000    2.196993000   -4.497145000
C    -0.574055000   -5.636738000   -4.010855000
C     3.016383000   -2.005851000   -4.108926000
C    -1.045900000    2.437791000   -3.735014000
C     1.262052000   -7.375238000   -3.749547000
C    -3.527198000   -3.161269000   -3.234425000
C     5.031935000   -2.943164000   -2.930546000
C    -5.414048000   -6.084500000   -2.258726000
C    -3.513020000   -1.750445000   -3.137154000
C     0.349035000   -6.369056000   -3.021364000
C    -5.462988000    2.351166000   -2.404805000
C     1.924926000   -4.376455000   -2.780491000
C     3.581622000   -2.447560000   -2.750494000
C    -3.989904000   -5.499595000   -2.307030000
C    -5.325076000    0.955329000   -2.356893000
C    -0.928256000    3.425393000   -2.571718000
C     0.162155000    4.316210000   -2.515252000
C    -4.364095000    3.167160000   -2.101757000
C    -4.001786000   -3.979660000   -2.190873000
C     2.639528000    5.935008000   -2.313207000
C     3.269534000    1.963729000   -2.244929000
C     1.169470000   -5.401148000   -2.176739000
C    -4.013650000   -1.132653000   -1.953386000
C     2.731764000   -3.496103000   -2.033805000
C    -4.118047000    0.369625000   -1.914515000
C     5.375845000    0.740680000   -1.583728000
C     0.864195000    7.706413000   -1.977304000
C    -3.138556000    2.602006000   -1.681404000
C    -3.088371000   -6.140184000   -1.233342000
C    -1.939103000    3.498907000   -1.559945000
C     0.281191000    5.301749000   -1.514991000
C     1.401340000    6.338968000   -1.503829000
C    -3.034972000    1.193861000   -1.506888000
C     3.934603000    1.098745000   -1.171763000
C    -4.468533000   -3.347042000   -1.019001000
C    -4.483458000   -1.944942000   -0.880989000
C     1.223269000   -5.520912000   -0.773627000
C     2.768117000   -3.631787000   -0.612231000
C    -1.833734000    4.483204000   -0.536374000
C    -0.728230000    5.360889000   -0.535406000
C    -6.504958000   -1.494172000    0.574529000
C     2.004231000   -4.650507000    0.019862000
C     3.895943000    1.705680000    0.227899000
C    -3.713558000    5.912599000    0.370247000
C     3.787417000    3.101983000    0.382251000
```

C	3.679066000	-2.740737000	0.186030000
C	4.978665000	-3.212898000	0.470616000
C	-4.982948000	-1.328462000	0.417293000
C	-2.883748000	4.628662000	0.561180000
C	3.272156000	-1.449217000	0.618931000
C	5.900938000	-2.399580000	1.145495000
C	4.251154000	-0.589330000	1.200256000
C	5.548011000	-1.082729000	1.468377000
C	4.008536000	0.881154000	1.392932000
C	3.758358000	3.722035000	1.647902000
C	3.580638000	5.231186000	1.772706000
C	-4.217194000	-1.856421000	1.642710000
C	2.010420000	-4.816797000	1.536130000
C	-2.525256000	1.159574000	1.441999000
C	2.694746000	-6.131791000	1.951963000
C	4.672380000	5.877340000	2.643289000
C	-3.873370000	1.568375000	1.615814000
C	-2.271093000	4.573368000	1.971726000
C	0.600397000	-4.686126000	2.138807000
C	2.172991000	5.589150000	2.292975000
C	4.014551000	1.495004000	2.678329000
C	3.867692000	2.895868000	2.778999000
C	-1.784502000	1.035239000	2.634598000
C	1.177096000	-0.912512000	2.782807000
C	-4.421823000	1.724105000	2.898388000
C	0.173407000	-0.099453000	3.338707000
C	4.212882000	0.694029000	3.961376000
C	1.829500000	-1.774244000	3.698911000
C	5.651488000	0.858859000	4.493093000
C	-2.294447000	1.128479000	3.937013000
C	-3.656777000	1.472804000	4.057484000
C	-0.263059000	-0.120505000	4.674929000
C	3.197009000	1.069496000	5.051259000
C	1.431264000	-1.839740000	5.043243000
C	-1.309557000	0.926660000	5.100778000
C	-0.563307000	2.273452000	5.328304000
C	0.379110000	-1.036037000	5.531738000
C	-2.031187000	0.522010000	6.394073000
K	-0.937440000	-2.848646000	-0.838564000
K	0.811802000	2.572090000	0.847957000
Ge	-1.742179000	0.437233000	-0.207847000
Ge	1.322705000	-1.097837000	0.824002000
O	-0.435549000	0.774764000	2.451970000
B	0.194809000	0.001496000	-0.446007000
H	-3.717727000	-0.051346000	-6.183144000
H	-4.425546000	-1.654631000	-5.788063000
H	-2.221043000	2.129462000	-5.555183000
H	-1.311981000	-0.788808000	-5.694667000
H	-1.899427000	-2.456198000	-5.451731000
H	-1.906552000	3.853756000	-5.173224000
H	-4.971748000	-0.189193000	-4.908866000
H	0.121735000	1.369442000	-5.221053000
H	0.006794000	-5.082919000	-4.776657000

H	-3.140743000	2.936690000	-4.249821000
H	-1.232127000	-6.351360000	-4.546395000
H	0.576830000	3.090227000	-5.077580000
H	3.120260000	-2.800533000	-4.877651000
H	3.576273000	-1.123889000	-4.480976000
H	-3.159994000	-3.639322000	-4.156344000
H	1.934929000	-6.852582000	-4.461000000
H	-0.920850000	-1.646845000	-4.176152000
H	0.665101000	-8.113126000	-4.325068000
H	-6.053694000	-5.642922000	-3.048765000
H	-2.723775000	0.079980000	-3.883721000
H	5.052521000	-3.869533000	-3.542424000
H	-1.221895000	-4.899617000	-3.494763000
H	5.642477000	-2.176041000	-3.450516000
H	1.895824000	-4.266490000	-3.874073000
H	-1.345658000	1.466412000	-3.288879000
H	-5.397299000	-7.184960000	-2.400734000
H	1.084721000	1.906523000	-3.820699000
H	1.948462000	-1.724718000	-4.040250000
H	-3.556376000	-5.745745000	-3.300391000
H	-6.417454000	2.802524000	-2.716235000
H	2.423623000	5.903888000	-3.401182000
H	0.931393000	4.251998000	-3.295091000
H	3.194496000	1.391392000	-3.190008000
H	-6.164067000	0.303481000	-2.646412000
H	1.899454000	-7.928844000	-3.031459000
H	5.385834000	0.283242000	-2.594331000
H	0.524600000	7.643395000	-3.032171000
H	-4.440674000	4.259616000	-2.215545000
H	5.515623000	-3.159615000	-1.960171000
H	-5.898250000	-5.878724000	-1.281466000
H	3.846613000	2.886295000	-2.464847000
H	-0.293621000	-6.946460000	-2.320775000
H	3.459787000	6.665283000	-2.162008000
H	3.012640000	4.933839000	-2.021785000
H	3.607324000	-1.557428000	-2.089552000
H	6.012789000	1.649720000	-1.613464000
H	2.239171000	2.258716000	-1.962057000
H	-3.038922000	-7.241657000	-1.355555000
H	1.650464000	8.487267000	-1.914495000
H	5.834943000	0.020275000	-0.880404000
H	-0.000072000	8.036916000	-1.367483000
H	-2.048796000	-5.755888000	-1.291300000
H	3.352037000	0.156268000	-1.116265000
H	-3.468338000	-5.934029000	-0.211118000
H	-4.194599000	5.939952000	-0.627954000
H	-7.047474000	-1.055710000	-0.287546000
H	3.728611000	3.739584000	-0.511902000
H	-4.832683000	-3.962036000	-0.180172000
H	1.715259000	6.458737000	-0.442196000
H	0.640053000	-6.319971000	-0.285176000
H	-6.793784000	-2.564286000	0.645783000
H	5.257216000	-4.233120000	0.163533000

H	-3.079607000	6.820084000	0.459331000
H	-4.776310000	-0.244909000	0.355415000
H	-0.660421000	6.131400000	0.251056000
H	3.672694000	5.651137000	0.746753000
H	-3.569583000	3.764525000	0.474922000
H	-4.512304000	5.980896000	1.137467000
H	6.906886000	-2.778051000	1.382841000
H	-6.856390000	-0.986339000	1.496386000
H	-4.508534000	1.739455000	0.733026000
H	5.684101000	5.622709000	2.269435000
H	3.726905000	-6.189363000	1.551158000
H	2.138412000	-7.016187000	1.575814000
H	6.296178000	-0.409231000	1.912692000
H	-4.409105000	-2.935147000	1.824239000
H	-3.126086000	-1.701181000	1.508294000
H	4.572059000	6.982269000	2.648352000
H	-0.085532000	-5.477904000	1.768742000
H	1.376512000	5.243078000	1.601349000
H	2.614632000	-3.982578000	1.944540000
H	0.178317000	-3.687166000	1.894331000
H	-1.575011000	5.417592000	2.160378000
H	2.049107000	6.687003000	2.395193000
H	-1.731527000	3.619188000	2.132909000
H	-4.513572000	-1.297811000	2.553248000
H	-3.066452000	4.613009000	2.742287000
H	4.606581000	5.533507000	3.696381000
H	2.749695000	-6.213698000	3.057244000
H	-5.474991000	2.029057000	3.002440000
H	0.635281000	-4.760187000	3.244185000
H	1.984232000	5.134066000	3.287769000
H	6.410166000	0.556166000	3.745129000
H	3.853530000	3.350649000	3.781490000
H	2.641392000	-2.426808000	3.339179000
H	4.055127000	-0.373411000	3.709211000
H	5.853806000	1.917005000	4.762743000
H	-0.024656000	2.585360000	4.410947000
H	2.160794000	1.009772000	4.670035000
H	5.804183000	0.241896000	5.402758000
H	-4.120614000	1.567907000	5.049411000
H	3.368695000	2.095227000	5.440830000
H	-1.283105000	3.074855000	5.592933000
H	3.276749000	0.373594000	5.909340000
H	1.944232000	-2.531852000	5.729405000
H	0.183212000	2.172858000	6.142118000
H	-2.576945000	-0.434220000	6.268744000
H	0.082695000	-1.109853000	6.587538000
H	-2.756467000	1.300782000	6.701677000
H	-1.310838000	0.407574000	7.227869000
Cl	0.599220000	-0.409764000	-2.222983000

*

Ferrocene_r2scan-3c_ CPCM(THF) (redox potetntials)

```
* xyz 0 1
Fe    2.311645000    2.644430000   -1.897956000
C     4.194577000    2.943931000   -2.638902000
C     1.737077000    4.391287000   -1.001742000
C     3.290843000    2.602177000   -3.693408000
C     4.194592000    1.871993000   -1.692037000
C     1.737384000    3.319583000   -0.054622000
C     0.833089000    4.049158000   -2.055889000
C     2.732123000    1.319099000   -3.398102000
C     3.290724000    0.867791000   -2.161242000
C     0.833327000    2.315265000   -0.523232000
C     0.274462000    2.766125000   -1.760155000
H     4.754568000    3.866374000   -2.555168000
H     2.340614000    5.288131000   -0.947552000
H     3.047127000    3.220591000   -4.547601000
H     4.754522000    1.841185000   -0.766260000
H     2.341100000    3.263361000    0.841974000
H     0.632732000    4.641704000   -2.939215000
H     1.991620000    0.796516000   -3.989783000
H     3.046825000   -0.056031000   -1.652723000
H     0.632932000    1.366088000   -0.043238000
H    -0.423049000    2.217976000   -2.380249000
*
```

Ferrocenium_r2scan-3c_ CPCM(THF) (redox potetntials)

```
* xyz 1 1
Fe    2.312283000    2.643789000   -1.896637000
C     4.204247000    2.939059000   -2.644964000
C     1.728707000    4.397149000   -0.995223000
C     3.318779000    2.582444000   -3.711970000
C     4.241093000    1.842242000   -1.726929000
C     1.686265000    3.346997000   -0.024372000
C     0.803503000    4.063883000   -2.035733000
C     2.821710000    1.273897000   -3.448531000
C     3.389323000    0.819992000   -2.230528000
C     0.738680000    2.381168000   -0.464040000
C     0.197066000    2.819724000   -1.699363000
H     4.739556000    3.874067000   -2.544636000
H     2.355863000    5.278048000   -0.955970000
H     3.053836000    3.205180000   -4.556514000
H     4.791045000    1.808470000   -0.795374000
H     2.292805000    3.279852000    0.869502000
H     0.615104000    4.641449000   -2.931381000
H     2.089008000    0.742151000   -4.042287000
H     3.158266000   -0.114323000   -1.734604000
H     0.520321000    1.439431000    0.023532000
H    -0.508625000    2.272068000   -2.311077000
*
```

1a_r2scan-3c_CPCM(THF) (redox potetntials)

```
* xyz 0 1
Ge     0.027969000    -1.907211000    -0.239424000
Ge     0.376351000     1.595080000    -0.435866000
Cl    -1.465069000     0.144248000     1.772737000
O      1.894806000    -0.467169000    -1.939872000
C      3.850730000     4.277325000    -0.489915000
H      2.939001000     4.501137000    -1.051273000
C      4.224971000    -3.425858000    -0.124465000
H      4.978978000    -3.671087000    -0.864738000
C      0.675778000    -4.465772000     0.803650000
C      4.468010000    -2.402658000     0.784970000
C      3.442519000     3.709190000     0.857844000
C      0.666440000    -2.445641000    -2.005917000
C     -5.040777000     2.277083000    -1.308063000
C     -6.465982000     1.813202000    -1.544333000
H     -6.798347000     1.322959000    -0.617806000
C      4.674177000     3.278047000    -1.314178000
H      4.855346000     3.680384000    -2.317279000
H      4.148943000     2.323819000    -1.419562000
H      5.647355000     3.084584000    -0.849617000
C      3.501563000    -2.150286000     1.758936000
H      3.706778000    -1.388968000     2.505107000
C      5.716796000    -1.542551000     0.755283000
H      6.067919000    -1.458172000     1.795016000
C      2.104355000     3.674549000     1.297956000
C     -0.047078000     3.477645000    -0.033378000
C      1.805970000     3.177227000     2.597472000
C      2.027955000    -3.827019000     0.814691000
C     -2.480112000     3.364860000    -0.831683000
C     -3.452840000     3.365036000     0.196294000
C     -4.708450000     2.824352000    -0.067383000
H     -5.471626000     2.854221000     0.707496000
C      2.293411000    -2.837778000     1.805117000
C      0.993986000     4.272731000     0.489187000
C     -1.197830000     4.096709000    -0.560294000
C      2.929723000    -0.684572000    -4.523557000
C     -2.946682000    -3.261111000     0.895852000
C      1.454092000     1.826739000    -2.051075000
C     -1.212885000     5.487761000    -0.713004000
H     -2.090236000     5.958299000    -1.149331000
C     -1.740500000    -4.137560000     0.782018000
C      2.847562000     2.708984000     3.394680000
H      2.622889000     2.337989000     4.390092000
C      2.508102000     0.670194000    -3.957650000
C     -0.138608000     6.264435000    -0.295650000
H     -0.160570000     7.342631000    -0.428761000
C     -7.375768000     3.032945000    -1.771845000
H     -7.321629000     3.731758000    -0.930820000
H     -8.418629000     2.718586000    -1.893689000
H     -7.069439000     3.567408000    -2.679219000
C     -0.445968000    -3.637960000     0.573921000
```

C	-2.793040000	2.810657000	-2.084298000
C	-3.165602000	-3.078284000	-1.634295000
H	-2.157885000	-3.496864000	-1.557299000
C	-2.741598000	-3.504904000	3.450671000
H	-1.780130000	-3.939588000	3.156106000
C	3.031605000	-4.155267000	-0.120457000
C	0.250768000	-3.582317000	-2.709300000
H	-0.371950000	-4.330454000	-2.226520000
C	6.856903000	-2.117229000	-0.083741000
H	7.747389000	-1.489014000	0.021090000
H	6.593677000	-2.140642000	-1.148165000
H	7.118001000	-3.134563000	0.227212000
C	-1.900944000	-5.504499000	1.043503000
H	-2.905682000	-5.899693000	1.169122000
C	0.935036000	5.662664000	0.349545000
H	1.733668000	6.271857000	0.764966000
C	-3.440181000	-2.999383000	2.195206000
C	2.139879000	3.065878000	-4.017191000
H	2.215316000	4.009165000	-4.550747000
C	1.502918000	-1.564061000	-2.674823000
C	0.485144000	-5.811054000	1.118912000
H	1.342353000	-6.442051000	1.331165000
C	1.357144000	-2.619446000	2.987247000
H	0.323147000	-2.677996000	2.630683000
C	4.450092000	3.232534000	1.702357000
H	5.483655000	3.261949000	1.363576000
C	1.567796000	3.036098000	-2.749723000
H	1.176996000	3.952983000	-2.317703000
C	-3.197408000	4.015392000	1.548468000
H	-2.117235000	4.163835000	1.652484000
C	5.300712000	2.279354000	3.878997000
H	6.230301000	2.331365000	3.295193000
C	-0.800395000	-6.339346000	1.187790000
H	-0.943212000	-7.394764000	1.403085000
C	4.177536000	2.732018000	2.968157000
C	0.615812000	-3.748071000	-4.043027000
H	0.267698000	-4.619308000	-4.590409000
C	1.522122000	-1.261453000	3.667788000
H	0.713850000	-1.120499000	4.393052000
H	1.474905000	-0.443251000	2.940718000
H	2.469717000	-1.187442000	4.213599000
C	-3.660840000	3.157390000	2.731939000
H	-4.753091000	3.079515000	2.771358000
H	-3.330602000	3.616370000	3.670787000
H	-3.247786000	2.146017000	2.679235000
C	5.359275000	-0.125854000	0.275089000
H	4.576020000	0.325144000	0.893127000
H	4.996490000	-0.155530000	-0.759416000
H	6.238695000	0.527562000	0.310996000
C	1.981853000	0.699317000	-2.669362000
C	-2.440953000	-2.379523000	4.449899000
H	-3.360417000	-1.925611000	4.836315000
H	-1.839901000	-1.591700000	3.986785000

H	-1.883839000	-2.781396000	5.304083000
C	2.993132000	-0.675674000	-6.050478000
H	3.302896000	-1.656254000	-6.422820000
H	3.737220000	0.047060000	-6.396923000
H	2.023843000	-0.421607000	-6.492510000
C	-3.630841000	-2.765407000	-0.226275000
C	2.581350000	1.891723000	-4.629388000
H	2.976091000	1.936226000	-5.639459000
C	4.327426000	-1.047636000	-3.962055000
H	5.061488000	-0.296884000	-4.274678000
H	4.636831000	-2.027857000	-4.341489000
H	4.315422000	-1.089625000	-2.868509000
C	1.930655000	-1.710485000	-3.991368000
C	-6.443759000	0.219696000	2.256563000
H	-7.365770000	0.812279000	2.275796000
H	-5.638694000	0.843118000	1.856076000
H	-6.188110000	-0.038835000	3.290755000
C	-4.622328000	-2.275269000	2.333950000
H	-5.009421000	-2.088490000	3.333071000
C	-4.063461000	2.263494000	-2.297978000
H	-4.292573000	1.848261000	-3.275574000
C	0.105524000	4.698677000	3.647138000
H	-0.914904000	4.764480000	4.043048000
H	0.204386000	5.423590000	2.834558000
H	0.802155000	4.980554000	4.446071000
C	1.438640000	-2.818968000	-4.683065000
H	1.713623000	-2.981319000	-5.720523000
C	2.882981000	-5.298402000	-1.107691000
H	1.813808000	-5.518270000	-1.211464000
C	1.551051000	-3.752490000	4.009548000
H	2.575541000	-3.738750000	4.400453000
H	1.368304000	-4.733598000	3.559910000
H	0.859499000	-3.624652000	4.850460000
C	-6.623806000	0.817305000	-2.692150000
H	-7.650991000	0.438630000	-2.718823000
H	-5.947457000	-0.036102000	-2.583054000
H	-6.421456000	1.289052000	-3.660680000
C	4.629326000	5.591163000	-0.318604000
H	4.052999000	6.328199000	0.249920000
H	4.867990000	6.021209000	-1.298160000
H	5.571870000	5.417270000	0.213331000
C	0.398558000	3.262991000	3.176816000
H	-0.308972000	3.020044000	2.375993000
C	-5.325424000	-1.791642000	1.230543000
C	-3.086873000	-1.836046000	-2.527669000
H	-2.516589000	-1.035347000	-2.043321000
H	-4.083595000	-1.447354000	-2.762875000
H	-2.593300000	-2.083411000	-3.474735000
C	0.126023000	2.292320000	4.327279000
H	-0.941871000	2.314825000	4.570231000
H	0.674167000	2.571397000	5.234642000
H	0.389005000	1.265649000	4.062667000
C	-3.566308000	-4.610995000	4.126024000

H	-3.766274000	-5.437515000	3.436436000
H	-4.529924000	-4.217850000	4.470999000
H	-3.031238000	-5.008718000	4.996050000
C	-1.815483000	2.871183000	-3.240995000
H	-0.866974000	3.244918000	-2.847003000
C	-4.806652000	-2.033889000	-0.037509000
H	-5.348907000	-1.664760000	-0.905448000
C	-3.865951000	5.398066000	1.614085000
H	-4.954616000	5.299072000	1.526666000
H	-3.518332000	6.050877000	0.807664000
H	-3.642736000	5.883560000	2.571402000
C	-6.629586000	-1.041798000	1.405175000
H	-6.958834000	-0.732111000	0.403481000
C	-1.546271000	1.499492000	-3.870083000
H	-2.443528000	1.095420000	-4.352863000
H	-1.211540000	0.778053000	-3.116692000
H	-0.761672000	1.584233000	-4.631328000
C	-4.052589000	-4.157917000	-2.271101000
H	-3.678313000	-4.422369000	-3.267008000
H	-5.082010000	-3.795741000	-2.377096000
H	-4.071722000	-5.064380000	-1.656086000
C	5.120464000	0.831816000	4.349562000
H	4.213096000	0.722174000	4.954184000
H	5.973105000	0.520520000	4.963375000
H	5.045321000	0.150269000	3.496505000
C	5.442676000	3.226541000	5.079627000
H	5.591005000	4.260476000	4.750401000
H	6.297549000	2.934419000	5.699918000
H	4.543295000	3.194794000	5.705565000
C	-2.289262000	3.875124000	-4.302149000
H	-3.233010000	3.548722000	-4.754526000
H	-1.542390000	3.966030000	-5.099651000
H	-2.447836000	4.866024000	-3.862509000
C	3.439831000	-4.971243000	-2.499327000
H	3.115494000	-5.732506000	-3.217096000
H	4.535559000	-4.966881000	-2.499992000
H	3.091258000	-3.998440000	-2.853125000
C	-7.717651000	-1.947242000	1.998488000
H	-8.669020000	-1.407752000	2.068816000
H	-7.440706000	-2.276581000	3.006736000
H	-7.870071000	-2.838064000	1.379843000
B	-0.346696000	-0.053766000	0.339674000
C	3.588793000	-6.555892000	-0.568459000
H	4.666782000	-6.372049000	-0.486963000
H	3.437899000	-7.399356000	-1.252054000
H	3.225444000	-6.844707000	0.421634000

*

1a$^{\cdot-}$_r2scan-3c_CPCM(THF) (redox potetntials)

* xyz -1 2
C -0.894278000 1.529612000 -6.543010000
C 1.734795000 1.194026000 -5.211173000
C -0.792153000 1.802426000 -5.042533000
C -0.987533000 3.320188000 -4.795926000
C -3.058626000 0.546126000 -4.755188000
C 2.911889000 0.811826000 -4.561777000
C 0.562956000 1.385967000 -4.472321000
C -1.862143000 1.057448000 -4.244267000
C 3.375963000 -3.477485000 -3.844424000
C -4.008448000 -0.004957000 -3.890697000
C 1.056235000 -2.542400000 -3.472711000
C 2.928366000 0.569328000 -3.190971000
C 0.643465000 1.180045000 -3.098565000
C 2.442235000 -2.764077000 -2.856687000
C -4.939818000 3.150016000 -2.959428000
C 3.581185000 3.848482000 -2.417162000
C -1.658565000 0.927551000 -2.872441000
C -3.772684000 -0.080788000 -2.519961000
C 1.767569000 0.716471000 -2.414208000
C 5.972465000 3.759532000 -1.624004000
C 4.578824000 3.123934000 -1.503481000
C -2.554062000 0.350887000 -1.973300000
C 2.379431000 -3.499834000 -1.534756000
C 1.754845000 -4.749111000 -1.484191000
C -2.547745000 -3.581599000 -1.639996000
C -0.067322000 -7.139578000 -1.133795000
C -5.203676000 2.981036000 -1.457901000
C -4.860886000 -3.979658000 -0.709904000
C -6.392980000 3.871251000 -1.054865000
C 5.226267000 -1.985652000 -0.598808000
C 6.158063000 -0.961848000 -0.472535000
C -3.197907000 4.398395000 -0.947331000
C -3.564583000 -3.164657000 -0.575284000
C 2.298157000 -7.900233000 -0.729160000
C 1.190713000 -6.928005000 -0.289142000
C 3.857995000 -1.747992000 -0.408936000
C 1.721157000 -5.506685000 -0.315958000
C -3.993441000 3.299595000 -0.597742000
C 2.987320000 -2.976281000 -0.378864000
C 4.116788000 3.098682000 -0.057721000
C 5.733092000 0.298957000 -0.072332000
C 0.231158000 5.396477000 -0.907005000
C 3.405908000 -0.434359000 -0.153272000
C -1.193274000 5.910561000 -0.648960000
C 4.371062000 0.569593000 0.099928000
C -2.097726000 4.783763000 -0.193874000
C 3.808806000 4.316868000 0.558484000
C 3.990353000 1.904131000 0.675078000
C -3.651541000 2.540960000 0.538471000

C	-1.180296000	7.076280000	0.348660000
C	2.295171000	-4.958949000	0.830433000
C	2.927325000	-3.713864000	0.825689000
C	-3.041976000	-3.257807000	0.844463000
C	-3.640310000	0.043983000	0.876770000
C	-4.356874000	1.267068000	0.898890000
C	-2.258858000	-4.342071000	1.228904000
C	-1.837351000	4.088054000	0.988357000
C	-5.672991000	1.293368000	1.368069000
C	-4.225749000	-1.091615000	1.471362000
C	3.394194000	4.391933000	1.881633000
C	-6.292592000	0.129926000	1.813712000
C	-2.588049000	2.981945000	1.372662000
C	-5.560070000	-1.049196000	1.891121000
C	-3.410728000	-2.300046000	1.811765000
C	3.595376000	1.965563000	2.038063000
C	3.099832000	5.731341000	2.526725000
C	0.152343000	-0.975112000	1.909360000
C	3.595504000	-3.226655000	2.103272000
C	-1.788395000	-7.044307000	2.683667000
C	3.304137000	3.202102000	2.607624000
C	4.956076000	-3.913215000	2.304398000
C	4.226430000	6.127447000	3.493013000
C	-1.847181000	-4.532770000	2.550780000
C	3.546829000	0.712231000	2.899243000
C	-0.994664000	-5.743588000	2.887168000
C	-2.303847000	2.312962000	2.710515000
C	4.953026000	0.344290000	3.400865000
C	-3.009005000	-2.473992000	3.151138000
C	1.744531000	5.751410000	3.242361000
C	2.729095000	-3.432000000	3.353052000
C	-0.879838000	2.522486000	3.225880000
C	-2.246337000	-3.596189000	3.497236000
C	-3.320947000	2.791943000	3.760241000
C	2.582746000	0.796999000	4.084155000
C	-3.387116000	-1.489458000	4.249995000
C	-0.384915000	-5.712399000	4.287551000
C	-4.466909000	-2.088177000	5.164719000
C	-2.184977000	-1.032912000	5.087252000
Ge	1.547536000	0.187791000	-0.534191000
Ge	-1.933461000	0.025297000	-0.141485000
O	-0.477897000	1.371315000	-2.318051000
H	-0.133932000	2.097428000	-7.087372000
H	-1.868565000	1.850588000	-6.923551000
H	-0.763487000	0.465481000	-6.767772000
H	1.738145000	1.337215000	-6.287183000
H	-3.269076000	0.596028000	-5.818992000
H	-0.206362000	3.887315000	-5.315130000
H	-1.966901000	3.637099000	-5.173042000
H	3.821895000	0.676167000	-5.141492000
H	3.465289000	-2.896206000	-4.770203000
H	1.141232000	-1.914502000	-4.367809000
H	-4.949052000	-0.370873000	-4.294946000

H	2.990055000	-4.471019000	-4.102769000
H	0.586190000	-3.489082000	-3.765011000
H	-0.936563000	3.554748000	-3.727690000
H	3.894229000	3.754893000	-3.463426000
H	-5.764012000	2.711848000	-3.533353000
H	4.377918000	-3.602879000	-3.418459000
H	0.397322000	-2.030745000	-2.762684000
H	-4.874636000	4.208283000	-3.237090000
H	-4.014884000	2.652706000	-3.261056000
H	6.319025000	3.725750000	-2.663644000
H	3.847014000	0.220846000	-2.724721000
H	2.866509000	-1.778121000	-2.656968000
H	3.522028000	4.916409000	-2.178124000
H	2.580191000	3.415966000	-2.323579000
H	1.313954000	-5.146690000	-2.395048000
H	0.141194000	-7.015505000	-2.202307000
H	-2.924675000	-3.319729000	-2.635475000
H	-5.279448000	-3.871508000	-1.717702000
H	4.651574000	2.088390000	-1.848133000
H	-4.537028000	-0.505225000	-1.872818000
H	-7.282513000	3.610441000	-1.640713000
H	-2.366854000	-4.662473000	-1.630360000
H	5.949245000	4.809596000	-1.309070000
H	-3.431205000	4.958071000	-1.850102000
H	-0.855313000	-6.432220000	-0.859071000
H	6.704874000	3.236753000	-0.999753000
H	2.585750000	-7.700040000	-1.768437000
H	-6.154445000	4.923988000	-1.249830000
H	-0.452050000	-8.154980000	-0.988311000
H	-5.476075000	1.934572000	-1.276335000
H	-4.664144000	-5.044086000	-0.531924000
H	-1.595199000	6.284060000	-1.601474000
H	-1.592038000	-3.066747000	-1.490402000
H	7.214124000	-1.158805000	-0.638278000
H	5.555884000	-2.993773000	-0.839704000
H	0.865783000	6.196812000	-1.305638000
H	-3.815607000	-2.115834000	-0.755909000
H	1.951749000	-8.938241000	-0.662966000
H	-5.612930000	-3.646528000	0.014003000
H	0.227656000	4.568376000	-1.624676000
H	3.893969000	5.238075000	-0.015334000
H	6.460397000	1.084109000	0.121736000
H	-6.639593000	3.776929000	0.006390000
H	3.190151000	-7.791250000	-0.103122000
H	0.940633000	-7.162092000	0.755752000
H	-0.556835000	7.896045000	-0.026470000
H	-1.958584000	-5.069068000	0.478479000
H	-2.191703000	7.460994000	0.518745000
H	0.691919000	5.031938000	0.019123000
H	3.067594000	6.477662000	1.720195000
H	-6.209187000	2.237218000	1.405722000
H	-2.181555000	-7.120414000	1.664933000
H	2.275837000	-5.540803000	1.750446000

H	5.624369000	-3.734600000	1.457083000
H	5.194883000	6.147630000	2.981798000
H	-0.771115000	6.758884000	1.314555000
H	-7.326507000	0.155068000	2.147980000
H	-6.011515000	-1.945485000	2.309793000
H	3.777394000	-2.151814000	1.993069000
H	-0.397958000	-1.917133000	2.040934000
H	-1.006121000	4.404257000	1.610460000
H	3.198257000	-0.103191000	2.257020000
H	1.211029000	-1.186334000	2.107527000
H	5.650924000	0.197859000	2.571086000
H	-1.150826000	-7.916531000	2.869403000
H	-0.167107000	-5.745353000	2.162142000
H	4.821444000	-4.996389000	2.412357000
H	4.039271000	7.119236000	3.920853000
H	-0.138022000	2.243524000	2.468261000
H	-0.186041000	-0.309268000	2.715069000
H	-2.635456000	-7.083793000	3.379409000
H	-2.444632000	1.234088000	2.582430000
H	0.939683000	5.443383000	2.567313000
H	1.713724000	-3.050008000	3.210027000
H	4.292692000	5.407931000	4.317831000
H	5.442555000	-3.537246000	3.212491000
H	3.000888000	3.243663000	3.649688000
H	1.520349000	6.759414000	3.609382000
H	-0.698083000	3.561548000	3.524980000
H	5.345460000	1.141513000	4.044313000
H	2.658242000	-4.491955000	3.621746000
H	4.916601000	-0.582400000	3.986868000
H	1.588880000	1.127775000	3.770300000
H	-4.348327000	2.590683000	3.440206000
H	-3.808489000	-0.596916000	3.774459000
H	1.740238000	5.073569000	4.103442000
H	-3.217689000	3.871564000	3.925114000
H	3.177832000	-2.907945000	4.204895000
H	-0.718848000	1.896486000	4.110683000
H	-5.352515000	-2.386859000	4.594860000
H	0.297423000	-6.559847000	4.414270000
H	-1.943732000	-3.718255000	4.532658000
H	2.481406000	-0.193993000	4.540937000
H	2.946672000	1.479983000	4.860765000
H	-1.398604000	-0.609556000	4.455545000
H	0.180777000	-4.791664000	4.463382000
H	-1.159343000	-5.791665000	5.060139000
H	-3.151608000	2.280961000	4.715800000
H	-4.082294000	-2.975735000	5.681644000
H	-1.751376000	-1.859134000	5.661736000
H	-4.773971000	-1.359218000	5.923954000
H	-2.501678000	-0.263818000	5.801210000
B	-0.076706000	-0.341575000	0.456829000

*

$1a^{2-}$_r2scan-3c_CPCM(THF) (redox potetntials)

* xyz -2 1
Ge -0.846323000 -1.740198000 -0.616238000
Ge 0.881704000 1.604748000 -0.070783000
Cl -1.332308000 0.297435000 1.825746000
O 1.704606000 -0.496896000 -2.009177000
C 4.138332000 4.587894000 -0.113067000
H 3.242294000 4.812327000 -0.697757000
C 4.027357000 -3.278104000 -0.457378000
H 4.743691000 -3.572469000 -1.218285000
C 0.480043000 -4.197185000 0.626176000
C 4.359889000 -2.278012000 0.445546000
C 3.692128000 4.012723000 1.220090000
C 0.155592000 -2.252578000 -2.279309000
C -4.678037000 2.255196000 -1.357772000
C -6.033988000 1.666963000 -1.691818000
H -6.660695000 1.764430000 -0.794275000
C 4.980762000 3.591604000 -0.920486000
H 5.200986000 4.002174000 -1.912888000
H 4.442898000 2.648690000 -1.056734000
H 5.934630000 3.379852000 -0.423844000
C 3.435576000 -1.975757000 1.449806000
H 3.708743000 -1.229042000 2.190769000
C 5.654468000 -1.487223000 0.384926000
H 6.053740000 -1.448142000 1.410682000
C 2.339818000 3.922829000 1.596741000
C 0.267356000 3.531076000 0.161427000
C 2.013611000 3.441284000 2.894141000
C 1.827456000 -3.542995000 0.537106000
C -2.152693000 3.366575000 -0.743509000
C -3.178191000 3.350197000 0.226811000
C -4.417172000 2.788750000 -0.099075000
H -5.209937000 2.777538000 0.646371000
C 2.189098000 -2.586270000 1.523293000
C 1.232450000 4.413961000 0.708617000
C -0.885349000 4.112153000 -0.420410000
C 2.785877000 -0.646385000 -4.562884000
C -3.253597000 -3.447288000 0.940559000
C 1.690489000 1.843608000 -1.868309000
C -0.947993000 5.497268000 -0.627739000
H -1.822271000 5.914336000 -1.123092000
C -1.926798000 -4.140746000 0.838294000
C 3.031523000 2.984326000 3.725243000
H 2.777326000 2.588910000 4.704299000
C 2.548052000 0.706579000 -3.899160000
C 0.072770000 6.338606000 -0.199604000
H 0.016000000 7.409358000 -0.379364000
C -6.720939000 2.436816000 -2.828089000
H -6.817055000 3.500033000 -2.582314000
H -7.722783000 2.032941000 -3.015401000
H -6.145825000 2.354278000 -3.757574000
C -0.734813000 -3.479690000 0.452400000

C	-2.404681000	2.829950000	-2.020903000
C	-3.820868000	-4.145066000	-1.442471000
H	-2.750315000	-4.375449000	-1.413962000
C	-2.739254000	-2.948607000	3.401580000
H	-1.703019000	-2.973946000	3.042556000
C	2.786718000	-3.932431000	-0.417404000
C	-0.342761000	-3.256917000	-3.126370000
H	-1.129844000	-3.912908000	-2.758403000
C	6.725081000	-2.104645000	-0.513662000
H	7.652286000	-1.525985000	-0.442432000
H	6.410603000	-2.096550000	-1.564482000
H	6.944988000	-3.140282000	-0.231600000
C	-1.920711000	-5.493295000	1.197918000
H	-2.864236000	-5.978963000	1.437259000
C	1.133001000	5.798086000	0.514726000
H	1.894411000	6.449826000	0.938398000
C	-3.641243000	-2.879996000	2.176535000
C	2.546731000	3.119765000	-3.770332000
H	2.751726000	4.085802000	-4.227529000
C	1.161870000	-1.460052000	-2.836732000
C	0.459846000	-5.544959000	1.010353000
H	1.402481000	-6.066931000	1.149415000
C	1.269461000	-2.281671000	2.696144000
H	0.258094000	-2.178567000	2.288501000
C	4.680934000	3.540991000	2.092415000
H	5.724374000	3.583105000	1.784416000
C	1.995590000	3.065896000	-2.492740000
H	1.748885000	3.996032000	-1.985879000
C	-2.997081000	3.986952000	1.597808000
H	-1.920507000	4.091263000	1.772883000
C	5.458339000	2.435048000	4.226345000
H	6.418500000	2.614466000	3.722140000
C	-0.732808000	-6.211430000	1.252939000
H	-0.732752000	-7.264002000	1.525086000
C	4.373573000	3.001385000	3.333001000
C	0.087350000	-3.395335000	-4.443610000
H	-0.340065000	-4.171100000	-5.075380000
C	1.593974000	-0.981814000	3.429931000
H	0.810874000	-0.788670000	4.171193000
H	1.627099000	-0.131281000	2.739667000
H	2.548620000	-1.041775000	3.967913000
C	-3.576638000	3.149718000	2.745357000
H	-4.672221000	3.126694000	2.715919000
H	-3.284885000	3.590608000	3.705930000
H	-3.208770000	2.120483000	2.711801000
C	5.365912000	-0.039813000	-0.047816000
H	4.634257000	0.439824000	0.611651000
H	4.955611000	-0.021248000	-1.065245000
H	6.283613000	0.560330000	-0.036572000
C	2.018421000	0.704081000	-2.610764000
C	-2.882964000	-1.752980000	4.351676000
H	-3.826118000	-1.795310000	4.909223000
H	-2.838456000	-0.801497000	3.816378000

H	-2.069872000	-1.771176000	5.086570000
C	2.919170000	-0.527251000	-6.081670000
H	3.107437000	-1.508179000	-6.528171000
H	3.768998000	0.111429000	-6.341115000
H	2.014611000	-0.104792000	-6.533067000
C	-4.160006000	-3.456021000	-0.134103000
C	2.818731000	1.949214000	-4.483181000
H	3.226951000	2.012203000	-5.487044000
C	4.097113000	-1.250048000	-3.999809000
H	4.940422000	-0.583066000	-4.215383000
H	4.289559000	-2.226891000	-4.459008000
H	4.030253000	-1.388795000	-2.916215000
C	1.629878000	-1.557421000	-4.151416000
C	-7.006281000	-0.117855000	1.808620000
H	-7.978322000	0.388328000	1.782731000
H	-6.310680000	0.432839000	1.166869000
H	-6.627655000	-0.065288000	2.835894000
C	-4.898208000	-2.282861000	2.283375000
H	-5.188291000	-1.829953000	3.227642000
C	-3.655822000	2.283263000	-2.304534000
H	-3.837079000	1.875283000	-3.296315000
C	0.148709000	4.883415000	3.778789000
H	-0.897076000	4.889711000	4.110298000
H	0.245024000	5.575365000	2.937370000
H	0.770691000	5.254379000	4.603215000
C	1.053106000	-2.532491000	-4.968465000
H	1.366258000	-2.642274000	-6.002204000
C	2.562566000	-5.083276000	-1.386844000
H	1.494150000	-5.327702000	-1.377149000
C	1.271478000	-3.442742000	3.706199000
H	2.275850000	-3.577375000	4.127856000
H	0.963350000	-4.384915000	3.244480000
H	0.582642000	-3.224744000	4.532053000
C	-5.921935000	0.175968000	-2.032665000
H	-6.914650000	-0.264207000	-2.187229000
H	-5.422226000	-0.375273000	-1.229414000
H	-5.339977000	0.029239000	-2.950128000
C	4.908320000	5.902501000	0.085812000
H	4.310119000	6.634848000	0.638817000
H	5.177373000	6.338597000	-0.883681000
H	5.834373000	5.731257000	0.647585000
C	0.575671000	3.456227000	3.394834000
H	-0.055373000	3.123902000	2.562017000
C	-5.792234000	-2.248784000	1.213910000
C	-4.072153000	-3.257933000	-2.665851000
H	-3.516127000	-2.318342000	-2.569549000
H	-5.136982000	-3.027275000	-2.787914000
H	-3.735307000	-3.767517000	-3.576524000
C	0.301879000	2.516650000	4.570139000
H	-0.777115000	2.479974000	4.758123000
H	0.785217000	2.863134000	5.491895000
H	0.641463000	1.498782000	4.361392000
C	-2.996559000	-4.240952000	4.196442000

H	-2.822137000	-5.132992000	3.589970000
H	-4.034787000	-4.262440000	4.550790000
H	-2.336624000	-4.285652000	5.071525000
C	-1.357624000	2.887069000	-3.112450000
H	-0.403569000	3.110070000	-2.630985000
C	-5.413601000	-2.859783000	0.021913000
H	-6.112362000	-2.857843000	-0.812710000
C	-3.615812000	5.394157000	1.626381000
H	-4.697749000	5.335966000	1.454347000
H	-3.181757000	6.038595000	0.856266000
H	-3.450438000	5.864639000	2.603397000
C	-7.143298000	-1.574910000	1.346910000
H	-7.601968000	-1.571193000	0.347948000
C	-1.188774000	1.555729000	-3.852849000
H	-2.067979000	1.313101000	-4.461756000
H	-1.017748000	0.734639000	-3.147754000
H	-0.321761000	1.610820000	-4.521533000
C	-4.585402000	-5.472263000	-1.563829000
H	-4.296342000	-6.000140000	-2.480563000
H	-5.666971000	-5.293611000	-1.600592000
H	-4.379190000	-6.126115000	-0.709259000
C	5.291176000	0.917901000	4.394646000
H	4.351903000	0.687532000	4.910956000
H	6.115032000	0.498564000	4.984092000
H	5.270155000	0.415841000	3.420744000
C	5.505532000	3.131656000	5.592526000
H	5.634030000	4.213506000	5.478611000
H	6.338173000	2.745891000	6.192071000
H	4.580225000	2.957332000	6.153445000
C	-1.663710000	4.021714000	-4.101191000
H	-2.614152000	3.840940000	-4.619053000
H	-0.871413000	4.093426000	-4.856768000
H	-1.735723000	4.986313000	-3.586208000
C	2.949972000	-4.745430000	-2.829999000
H	2.642087000	-5.558447000	-3.498473000
H	4.034398000	-4.625909000	-2.937593000
H	2.465274000	-3.826904000	-3.163599000
C	-8.068441000	-2.357540000	2.289584000
H	-9.057069000	-1.886448000	2.342751000
H	-7.650590000	-2.383846000	3.302948000
H	-8.195298000	-3.390737000	1.948519000
B	-0.333470000	0.027135000	0.241928000
C	3.353084000	-6.324913000	-0.935525000
H	4.429863000	-6.121771000	-0.986888000
H	3.137819000	-7.176103000	-1.592864000
H	3.120392000	-6.617111000	0.092666000

*

1b_r2scan-3c_CPCM(THF) (redox potetntials)

```
* xyz 0 1
C     -0.107816000    2.643742000   -6.203365000
C     -2.460810000    1.487889000   -4.786125000
C      2.340068000    1.900616000   -4.711740000
C     -0.142036000    2.687990000   -4.676376000
C      3.461729000   -3.078914000   -4.237877000
C     -0.292045000    4.160333000   -4.218361000
C      3.406720000    1.287681000   -4.052038000
C     -3.502040000    0.848041000   -4.109207000
C     -1.313358000    1.891612000   -4.100911000
C      1.123910000    2.097788000   -4.055591000
C      1.267781000   -1.861721000   -3.926961000
C      2.512061000   -2.422317000   -3.226726000
C     -4.410210000    4.003488000   -2.957977000
C      1.319751000   -7.642772000   -2.607132000
C      3.258944000    0.805089000   -2.755978000
C     -3.416042000    0.585549000   -2.743164000
C      1.051189000    1.667469000   -2.733352000
C     -1.253484000    1.562710000   -2.749528000
C      1.376753000   -4.493114000   -2.402366000
C      2.154615000   -3.375518000   -2.106232000
C      0.331246000   -6.711052000   -1.883783000
C      2.049193000    0.964923000   -2.066686000
C     -2.251729000    0.914103000   -2.038316000
C     -2.155064000   -3.118919000   -1.938823000
C     -4.632493000   -3.441392000   -1.556633000
C      4.435303000    3.707281000   -1.546853000
C     -4.749861000    3.689419000   -1.496068000
C      1.055384000   -5.451144000   -1.443387000
C     -5.811777000    4.689014000   -1.001651000
C     -3.358009000   -2.731307000   -1.074896000
C     -0.374114000   -7.466884000   -0.759704000
C      5.124125000    2.696265000   -0.620129000
C      6.587542000    3.107733000   -0.388689000
C     -2.569373000    4.703295000   -0.778903000
C      2.622781000   -3.185988000   -0.790539000
C      4.981453000   -2.514935000   -0.527620000
C     -3.544802000    3.723284000   -0.574880000
C      3.637797000   -2.126073000   -0.450035000
C      1.000668000    5.198164000   -0.335639000
C      5.999321000   -1.666852000   -0.109616000
C     -0.383209000    5.855089000   -0.236501000
C      1.491865000   -5.233391000   -0.138601000
C      3.324707000   -0.829155000    0.008842000
C     -1.461209000    4.831279000    0.051681000
C      2.267452000   -4.120829000    0.207132000
C      5.681575000   -0.436268000    0.452916000
C     -3.151503000   -2.981939000    0.406777000
C      4.353610000   -0.006151000    0.520588000
C     -3.387067000    2.815728000    0.493923000
C      4.402876000    2.533676000    0.706181000
```

C	-3.629132000	0.347894000	0.614506000
C	-4.258390000	1.611970000	0.665753000
C	-0.380895000	6.973593000	0.815180000
C	-2.543153000	-4.160763000	0.832658000
C	-5.617231000	1.691580000	0.972961000
C	-6.350774000	0.532142000	1.209615000
C	-4.332330000	-0.799980000	1.011435000
C	-5.708086000	-0.699101000	1.253788000
C	4.048092000	1.277286000	1.237478000
C	4.132526000	3.688449000	1.448042000
C	-3.636727000	-2.074828000	1.369782000
C	-1.378189000	4.001585000	1.168049000
C	-2.317126000	3.002723000	1.417068000
C	2.771441000	-3.995460000	1.637336000
C	4.015012000	-4.872226000	1.858606000
C	-2.784399000	-6.957471000	2.272197000
C	-0.125987000	-1.203769000	1.817580000
C	-2.412136000	-4.483214000	2.185455000
C	-1.800123000	-5.815661000	2.576711000
C	3.480713000	1.208696000	2.538872000
C	3.545839000	3.641585000	2.705483000
C	-3.483899000	-2.364918000	2.742116000
C	1.707210000	-4.348035000	2.684581000
C	-2.262744000	2.240210000	2.737421000
C	-2.885838000	-3.570151000	3.120983000
C	3.325248000	4.912798000	3.500455000
C	3.234764000	2.388616000	3.237157000
C	3.253793000	-0.129047000	3.228055000
C	-0.888639000	2.245814000	3.405310000
C	4.337158000	5.009210000	4.652335000
C	4.575931000	-0.653389000	3.815138000
C	-3.949134000	-1.417299000	3.840122000
C	-3.319394000	2.815154000	3.696542000
C	-1.339458000	-5.892105000	4.032093000
C	-5.176989000	-1.983457000	4.569056000
C	1.892595000	5.035622000	4.029164000
C	2.185436000	-0.103482000	4.323125000
C	-2.836078000	-1.097903000	4.847566000
O	-0.118311000	1.855852000	-2.025443000
Ge	1.584167000	0.009201000	-0.416752000
Ge	-1.828891000	0.383848000	-0.198951000
B	-0.117158000	-0.324291000	0.488513000
H	-1.025951000	3.072709000	-6.615295000
H	0.724794000	3.242388000	-6.583653000
H	-0.001426000	1.618882000	-6.574804000
H	-2.563616000	1.689511000	-5.847909000
H	2.460748000	2.209146000	-5.745476000
H	3.757415000	-2.354779000	-5.006375000
H	1.556135000	-1.077617000	-4.636493000
H	2.977692000	-3.926355000	-4.737333000
H	4.350800000	1.150805000	-4.572222000
H	-4.397496000	0.564275000	-4.654987000
H	-1.212481000	4.586219000	-4.633254000

H	0.562210000	4.748420000	-4.571583000
H	0.735643000	-2.643293000	-4.481326000
H	4.367837000	-3.447391000	-3.744238000
H	-5.264231000	3.755905000	-3.597982000
H	1.795874000	-7.138341000	-3.454045000
H	0.805461000	-8.535719000	-2.980448000
H	1.030288000	-4.639594000	-3.424335000
H	0.572329000	-1.424020000	-3.201842000
H	-3.548112000	3.427986000	-3.301468000
H	-4.194648000	5.068510000	-3.098833000
H	-0.338878000	4.234478000	-3.127576000
H	3.047635000	-1.581691000	-2.781278000
H	-2.310241000	-2.775195000	-2.968095000
H	-4.821268000	-3.217804000	-2.613120000
H	4.918194000	3.701270000	-2.530401000
H	-0.430089000	-6.402708000	-2.615740000
H	2.108143000	-7.964292000	-1.915776000
H	4.079447000	0.266269000	-2.291322000
H	-4.248611000	0.103917000	-2.235979000
H	-2.009122000	-4.204558000	-1.972114000
H	-6.714627000	4.623356000	-1.619811000
H	-4.525510000	-4.527406000	-1.447758000
H	7.120501000	3.170157000	-1.344511000
H	3.378051000	3.462685000	-1.686032000
H	-5.176253000	2.679816000	-1.455433000
H	-2.662591000	5.377214000	-1.626947000
H	-0.935344000	-8.311502000	-1.173107000
H	-1.232612000	-2.663156000	-1.561446000
H	4.504543000	4.725467000	-1.148313000
H	-5.504308000	-3.124237000	-0.973871000
H	-5.421850000	5.711327000	-1.075098000
H	5.128909000	1.725390000	-1.123145000
H	-3.524542000	-1.656412000	-1.205056000
H	-0.616466000	6.306039000	-1.211302000
H	1.004743000	4.388034000	-1.073089000
H	5.220262000	-3.501827000	-0.916220000
H	1.755176000	5.936398000	-0.630556000
H	-1.076254000	-6.824639000	-0.221321000
H	0.342457000	-7.870666000	-0.034916000
H	6.640209000	4.090500000	0.094210000
H	7.036767000	-1.981247000	-0.185359000
H	-6.092534000	4.515853000	0.041025000
H	7.110684000	2.389965000	0.251297000
H	-2.169972000	-4.861157000	0.089006000
H	0.373785000	7.729263000	0.569551000
H	1.247167000	-5.955752000	0.634348000
H	1.308487000	4.773645000	0.627291000
H	6.467096000	0.198026000	0.855701000
H	-1.357993000	7.465192000	0.870830000
H	-6.101086000	2.660365000	1.046017000
H	4.402979000	4.658195000	1.035444000
H	-7.415937000	0.596539000	1.414613000
H	-3.098993000	-6.946503000	1.223678000

H	-6.263085000	-1.594705000	1.521121000
H	4.825173000	-4.604669000	1.174513000
H	-0.145636000	6.570661000	1.807128000
H	3.765244000	-5.927865000	1.697418000
H	-0.916743000	-5.956978000	1.937067000
H	-0.773706000	-2.084433000	1.721575000
H	3.066259000	-2.951164000	1.795188000
H	-0.550179000	4.130381000	1.857066000
H	-2.324026000	-7.929694000	2.483053000
H	0.873704000	-1.562848000	2.087567000
H	-3.680837000	-6.858723000	2.896273000
H	3.507695000	5.755313000	2.818610000
H	0.755609000	-3.847475000	2.482967000
H	2.927820000	-0.842434000	2.464164000
H	-2.529859000	1.194278000	2.551836000
H	1.520093000	-5.427155000	2.718294000
H	-0.501153000	-0.640282000	2.681612000
H	4.381010000	-4.760711000	2.886111000
H	5.365331000	4.951495000	4.279552000
H	-0.104430000	1.916764000	2.713561000
H	5.346853000	-0.758536000	3.046568000
H	-4.324742000	2.750455000	3.268270000
H	-4.244093000	-0.470509000	3.374955000
H	-5.996793000	-2.185581000	3.872381000
H	2.052147000	-4.045948000	3.680127000
H	-0.622781000	3.238878000	3.783897000
H	-0.802374000	-6.831320000	4.201526000
H	-2.781893000	-3.787489000	4.179530000
H	-3.103843000	3.868806000	3.911027000
H	4.216824000	5.955092000	5.192662000
H	1.165759000	4.962902000	3.213896000
H	2.807847000	2.331445000	4.234037000
H	-0.669702000	-5.065066000	4.289501000
H	4.419728000	-1.633585000	4.281282000
H	1.257242000	0.355308000	3.973179000
H	4.187133000	4.189165000	5.364467000
H	-0.898841000	1.563226000	4.261754000
H	4.947038000	0.036569000	4.582726000
H	-2.191584000	-5.871079000	4.721581000
H	-1.948363000	-0.701808000	4.345155000
H	1.752273000	6.000324000	4.529464000
H	-3.311509000	2.262260000	4.643068000
H	-4.925100000	-2.922322000	5.076273000
H	1.960330000	-1.129393000	4.635080000
H	-5.533145000	-1.274274000	5.325154000
H	1.667915000	4.246213000	4.755183000
H	2.527556000	0.440767000	5.210894000
H	-2.536716000	-1.984613000	5.417060000
H	-3.187698000	-0.346216000	5.563371000

*

1b$^{\cdot-}$_r2scan-3c_CPCM(THF) (redox potetntials)

```
* xyz -1 2
C    -0.894278000    1.529612000    -6.543010000
C     1.734795000    1.194026000    -5.211173000
C    -0.792153000    1.802426000    -5.042533000
C    -0.987533000    3.320188000    -4.795926000
C    -3.058626000    0.546126000    -4.755188000
C     2.911889000    0.811826000    -4.561777000
C     0.562956000    1.385967000    -4.472321000
C    -1.862143000    1.057448000    -4.244267000
C     3.375963000   -3.477485000    -3.844424000
C    -4.008448000   -0.004957000    -3.890697000
C     1.056235000   -2.542400000    -3.472711000
C     2.928366000    0.569328000    -3.190971000
C     0.643465000    1.180045000    -3.098565000
C     2.442235000   -2.764077000    -2.856687000
C    -4.939818000    3.150016000    -2.959428000
C     3.581185000    3.848482000    -2.417162000
C    -1.658565000    0.927551000    -2.872441000
C    -3.772684000   -0.080788000    -2.519961000
C     1.767569000    0.716471000    -2.414208000
C     5.972465000    3.759532000    -1.624004000
C     4.578824000    3.123934000    -1.503481000
C    -2.554062000    0.350887000    -1.973300000
C     2.379431000   -3.499834000    -1.534756000
C     1.754845000   -4.749111000    -1.484191000
C    -2.547745000   -3.581599000    -1.639996000
C    -0.067322000   -7.139578000    -1.133795000
C    -5.203676000    2.981036000    -1.457901000
C    -4.860886000   -3.979658000    -0.709904000
C    -6.392980000    3.871251000    -1.054865000
C     5.226267000   -1.985652000    -0.598808000
C     6.158063000   -0.961848000    -0.472535000
C    -3.197907000    4.398395000    -0.947331000
C    -3.564583000   -3.164657000    -0.575284000
C     2.298157000   -7.900233000    -0.729160000
C     1.190713000   -6.928005000    -0.289142000
C     3.857995000   -1.747992000    -0.408936000
C     1.721157000   -5.506685000    -0.315958000
C    -3.993441000    3.299595000    -0.597742000
C     2.987320000   -2.976281000    -0.378864000
C     4.116788000    3.098682000    -0.057721000
C     5.733092000    0.298957000    -0.072332000
C     0.231158000    5.396477000    -0.907005000
C     3.405908000   -0.434359000    -0.153272000
C    -1.193274000    5.910561000    -0.648960000
C     4.371062000    0.569593000     0.099928000
C    -2.097726000    4.783763000    -0.193874000
C     3.808806000    4.316868000     0.558484000
C     3.990353000    1.904131000     0.675078000
C    -3.651541000    2.540960000     0.538471000
C    -1.180296000    7.076280000     0.348660000
```

C	2.295171000	-4.958949000	0.830433000
C	2.927325000	-3.713864000	0.825689000
C	-3.041976000	-3.257807000	0.844463000
C	-3.640310000	0.043983000	0.876770000
C	-4.356874000	1.267068000	0.898890000
C	-2.258858000	-4.342071000	1.228904000
C	-1.837351000	4.088054000	0.988357000
C	-5.672991000	1.293368000	1.368069000
C	-4.225749000	-1.091615000	1.471362000
C	3.394194000	4.391933000	1.881633000
C	-6.292592000	0.129926000	1.813712000
C	-2.588049000	2.981945000	1.372662000
C	-5.560070000	-1.049196000	1.891121000
C	-3.410728000	-2.300046000	1.811765000
C	3.595376000	1.965563000	2.038063000
C	3.099832000	5.731341000	2.526725000
C	0.152343000	-0.975112000	1.909360000
C	3.595504000	-3.226655000	2.103272000
C	-1.788395000	-7.044307000	2.683667000
C	3.304137000	3.202102000	2.607624000
C	4.956076000	-3.913215000	2.304398000
C	4.226430000	6.127447000	3.493013000
C	-1.847181000	-4.532770000	2.550780000
C	3.546829000	0.712231000	2.899243000
C	-0.994664000	-5.743588000	2.887168000
C	-2.303847000	2.312962000	2.710515000
C	4.953026000	0.344290000	3.400865000
C	-3.009005000	-2.473992000	3.151138000
C	1.744531000	5.751410000	3.242361000
C	2.729095000	-3.432000000	3.353052000
C	-0.879838000	2.522486000	3.225880000
C	-2.246337000	-3.596189000	3.497236000
C	-3.320947000	2.791943000	3.760241000
C	2.582746000	0.796999000	4.084155000
C	-3.387116000	-1.489458000	4.249995000
C	-0.384915000	-5.712399000	4.287551000
C	-4.466909000	-2.088177000	5.164719000
C	-2.184977000	-1.032912000	5.087252000
Ge	1.547536000	0.187791000	-0.534191000
Ge	-1.933461000	0.025297000	-0.141485000
O	-0.477897000	1.371315000	-2.318051000
H	-0.133932000	2.097428000	-7.087372000
H	-1.868565000	1.850588000	-6.923551000
H	-0.763487000	0.465481000	-6.767772000
H	1.738145000	1.337215000	-6.287183000
H	-3.269076000	0.596028000	-5.818992000
H	-0.206362000	3.887315000	-5.315130000
H	-1.966901000	3.637099000	-5.173042000
H	3.821895000	0.676167000	-5.141492000
H	3.465289000	-2.896206000	-4.770203000
H	1.141232000	-1.914502000	-4.367809000
H	-4.949052000	-0.370873000	-4.294946000
H	2.990055000	-4.471019000	-4.102769000

H	0.586190000	-3.489082000	-3.765011000
H	-0.936563000	3.554748000	-3.727690000
H	3.894229000	3.754893000	-3.463426000
H	-5.764012000	2.711848000	-3.533353000
H	4.377918000	-3.602879000	-3.418459000
H	0.397322000	-2.030745000	-2.762684000
H	-4.874636000	4.208283000	-3.237090000
H	-4.014884000	2.652706000	-3.261056000
H	6.319025000	3.725750000	-2.663644000
H	3.847014000	0.220846000	-2.724721000
H	2.866509000	-1.778121000	-2.656968000
H	3.522028000	4.916409000	-2.178124000
H	2.580191000	3.415966000	-2.323579000
H	1.313954000	-5.146690000	-2.395048000
H	0.141194000	-7.015505000	-2.202307000
H	-2.924675000	-3.319729000	-2.635475000
H	-5.279448000	-3.871508000	-1.717702000
H	4.651574000	2.088390000	-1.848133000
H	-4.537028000	-0.505225000	-1.872818000
H	-7.282513000	3.610441000	-1.640713000
H	-2.366854000	-4.662473000	-1.630360000
H	5.949245000	4.809596000	-1.309070000
H	-3.431205000	4.958071000	-1.850102000
H	-0.855313000	-6.432220000	-0.859071000
H	6.704874000	3.236753000	-0.999753000
H	2.585750000	-7.700040000	-1.768437000
H	-6.154445000	4.923988000	-1.249830000
H	-0.452050000	-8.154980000	-0.988311000
H	-5.476075000	1.934572000	-1.276335000
H	-4.664144000	-5.044086000	-0.531924000
H	-1.595199000	6.284060000	-1.601474000
H	-1.592038000	-3.066747000	-1.490402000
H	7.214124000	-1.158805000	-0.638278000
H	5.555884000	-2.993773000	-0.839704000
H	0.865783000	6.196812000	-1.305638000
H	-3.815607000	-2.115834000	-0.755909000
H	1.951749000	-8.938241000	-0.662966000
H	-5.612930000	-3.646528000	0.014003000
H	0.227656000	4.568376000	-1.624676000
H	3.893969000	5.238075000	-0.015334000
H	6.460397000	1.084109000	0.121736000
H	-6.639593000	3.776929000	0.006390000
H	3.190151000	-7.791250000	-0.103122000
H	0.940633000	-7.162092000	0.755752000
H	-0.556835000	7.896045000	-0.026470000
H	-1.958584000	-5.069068000	0.478479000
H	-2.191703000	7.460994000	0.518745000
H	0.691919000	5.031938000	0.019123000
H	3.067594000	6.477662000	1.720195000
H	-6.209187000	2.237218000	1.405722000
H	-2.181555000	-7.120414000	1.664933000
H	2.275837000	-5.540803000	1.750446000
H	5.624369000	-3.734600000	1.457083000

H	5.194883000	6.147630000	2.981798000
H	-0.771115000	6.758884000	1.314555000
H	-7.326507000	0.155068000	2.147980000
H	-6.011515000	-1.945485000	2.309793000
H	3.777394000	-2.151814000	1.993069000
H	-0.397958000	-1.917133000	2.040934000
H	-1.006121000	4.404257000	1.610460000
H	3.198257000	-0.103191000	2.257020000
H	1.211029000	-1.186334000	2.107527000
H	5.650924000	0.197859000	2.571086000
H	-1.150826000	-7.916531000	2.869403000
H	-0.167107000	-5.745353000	2.162142000
H	4.821444000	-4.996389000	2.412357000
H	4.039271000	7.119236000	3.920853000
H	-0.138022000	2.243524000	2.468261000
H	-0.186041000	-0.309268000	2.715069000
H	-2.635456000	-7.083793000	3.379409000
H	-2.444632000	1.234088000	2.582430000
H	0.939683000	5.443383000	2.567313000
H	1.713724000	-3.050008000	3.210027000
H	4.292692000	5.407931000	4.317831000
H	5.442555000	-3.537246000	3.212491000
H	3.000888000	3.243663000	3.649688000
H	1.520349000	6.759414000	3.609382000
H	-0.698083000	3.561548000	3.524980000
H	5.345460000	1.141513000	4.044313000
H	2.658242000	-4.491955000	3.621746000
H	4.916601000	-0.582400000	3.986868000
H	1.588880000	1.127775000	3.770300000
H	-4.348327000	2.590683000	3.440206000
H	-3.808489000	-0.596916000	3.774459000
H	1.740238000	5.073569000	4.103442000
H	-3.217689000	3.871564000	3.925114000
H	3.177832000	-2.907945000	4.204895000
H	-0.718848000	1.896486000	4.110683000
H	-5.352515000	-2.386859000	4.594860000
H	0.297423000	-6.559847000	4.414270000
H	-1.943732000	-3.718255000	4.532658000
H	2.481406000	-0.193993000	4.540937000
H	2.946672000	1.479983000	4.860765000
H	-1.398604000	-0.609556000	4.455545000
H	0.180777000	-4.791664000	4.463382000
H	-1.159343000	-5.791665000	5.060139000
H	-3.151608000	2.280961000	4.715800000
H	-4.082294000	-2.975735000	5.681644000
H	-1.751376000	-1.859134000	5.661736000
H	-4.773971000	-1.359218000	5.923954000
H	-2.501678000	-0.263818000	5.801210000
B	-0.076706000	-0.341575000	0.456829000

*

1b^{2-}_r2scan-3c_CPCM(THF) (redox potetntials)

```
* xyz -2 1
C      0.359381000     1.245828000    -6.596780000
C      0.112405000     1.533061000    -5.115177000
C     -1.964101000    -0.016500000    -5.273175000
C     -0.334326000     3.009449000    -4.968329000
C      2.674995000     1.266157000    -4.766316000
C     -0.985959000     0.646010000    -4.527658000
C      1.365509000     1.313724000    -4.271503000
C     -2.993003000    -0.697861000    -4.615547000
C      3.734795000     1.039436000    -3.883097000
C     -4.182413000     2.533373000    -3.973334000
C      4.311793000     4.033094000    -1.529259000
C     -1.059604000     0.543809000    -3.134235000
C      1.189285000     1.137761000    -2.902115000
C     -3.024445000    -0.759735000    -3.224692000
C      3.504330000     0.832213000    -2.525553000
C      4.081965000    -3.202899000    -3.111970000
C      1.715226000    -2.350097000    -3.309781000
C     -3.819917000    -4.210594000    -1.751739000
C     -2.031245000    -0.167168000    -2.424767000
C      2.205379000     0.845934000    -1.982310000
C     -5.780063000     3.616228000    -2.387518000
C     -4.620352000     2.610747000    -2.506748000
C     -6.079981000    -3.972772000    -0.654533000
C      2.919814000    -2.518387000    -2.377541000
C      5.102348000     3.275495000    -0.455095000
C      6.498488000     3.892734000    -0.281087000
C     -4.630388000    -3.464055000    -0.687431000
C     -0.663902000    -6.205070000    -0.600062000
C      1.734714000     6.417057000     2.398592000
C     -3.489259000     2.987811000    -1.562917000
C      1.859671000    -4.448100000    -1.171995000
C      2.583218000    -3.257905000    -1.100759000
C      4.365119000     3.223005000     0.871834000
C      3.976939000     4.432117000     1.455524000
C     -2.662154000     4.060277000    -1.923577000
C     -4.009729000    -3.543497000     0.695236000
C     -5.442710000     1.420490000     0.405237000
C     -6.263768000     0.448139000     0.959847000
C     -4.099822000     1.159335000     0.102983000
C     -3.615992000    -4.783997000     1.201534000
C     -3.545146000    -0.133162000     0.326635000
C     -3.268399000     2.329720000    -0.336534000
C     -5.707500000    -0.782034000     1.291319000
C      3.484956000    -0.263567000     0.507384000
C      1.450701000    -7.517383000    -1.038282000
C      0.769557000    -6.485001000    -0.130388000
C     -4.366196000    -1.064228000     1.010456000
C      1.566886000    -5.199879000    -0.035425000
C      4.054520000     2.009624000     1.516963000
C      3.310786000     4.481115000     2.675445000
```

C	4.451412000	0.676750000	0.947838000
C	3.029001000	-2.785615000	0.149492000
C	2.839313000	5.795378000	3.265160000
C	3.933393000	-1.588459000	0.281986000
C	-1.645520000	4.518825000	-1.097991000
C	-3.867073000	-2.394200000	1.494331000
C	-0.712757000	5.626856000	-1.542060000
C	5.814754000	0.354289000	0.969050000
C	0.748431000	5.157039000	-1.533091000
C	5.304670000	-1.881500000	0.284118000
C	6.249912000	-0.907142000	0.585020000
C	3.992447000	6.786254000	3.469288000
C	-3.073950000	-4.917041000	2.477192000
C	-3.540752000	-6.778443000	4.111167000
C	-2.302033000	2.857041000	0.561530000
C	3.074862000	3.276611000	3.338998000
C	3.440026000	2.046683000	2.794519000
C	2.019534000	-4.727663000	1.192622000
C	-1.507480000	3.926328000	0.159402000
C	-2.622266000	-6.269407000	2.991333000
C	2.746535000	-3.538516000	1.309983000
C	-0.012636000	-0.850366000	1.984002000
C	-0.882206000	6.889697000	-0.685895000
C	-3.362345000	-2.516602000	2.809359000
C	-2.966711000	-3.774741000	3.268997000
C	-2.180729000	2.318362000	1.978973000
C	3.214757000	0.769453000	3.588943000
C	-1.161726000	-6.237098000	3.460210000
C	3.258814000	-3.128625000	2.683584000
C	-3.280984000	-1.318619000	3.747015000
C	-3.348060000	2.820279000	2.847375000
C	2.090862000	0.850510000	4.623024000
C	-4.605090000	-1.125363000	4.507373000
C	4.543933000	-3.895758000	3.035749000
C	-0.861239000	2.651386000	2.673667000
C	4.516061000	0.322281000	4.275496000
C	2.227566000	-3.322575000	3.804224000
C	-2.141601000	-1.400793000	4.771979000
Ge	-1.788368000	-0.746429000	-0.513951000
Ge	1.691261000	0.492830000	-0.103010000
O	-0.085929000	1.164050000	-2.376584000
B	-0.043169000	-0.353086000	0.455730000
H	-0.549126000	1.426667000	-7.179273000
H	1.131957000	1.912756000	-6.991676000
H	0.677871000	0.209888000	-6.757838000
H	-1.945765000	0.011456000	-6.358462000
H	2.873205000	1.393397000	-5.825989000
H	0.458353000	3.678876000	-5.323663000
H	-1.241473000	3.188443000	-5.558076000
H	-3.767739000	-1.191621000	-5.198653000
H	4.752573000	1.001633000	-4.267173000
H	-0.551916000	3.250163000	-3.923040000
H	-4.986601000	2.100824000	-4.580489000

H	4.832652000	3.974341000	-2.492255000
H	4.357153000	-2.631550000	-4.007307000
H	1.974035000	-1.669509000	-4.129155000
H	-4.257348000	-4.042150000	-2.743040000
H	-3.965222000	3.528254000	-4.379576000
H	4.198802000	5.092602000	-1.271510000
H	-3.295012000	1.909096000	-4.087705000
H	3.800508000	-4.215898000	-3.426391000
H	3.316104000	3.598321000	-1.656893000
H	-6.618058000	3.311725000	-3.026396000
H	-6.548594000	-3.864180000	-1.640118000
H	1.401981000	-3.304949000	-3.748985000
H	-3.810055000	-5.292289000	-1.573250000
H	-3.811007000	-1.337823000	-2.741104000
H	4.347462000	0.601702000	-1.877513000
H	7.046391000	3.874995000	-1.230818000
H	4.965572000	-3.280116000	-2.468456000
H	-2.786562000	-3.846650000	-1.764416000
H	-4.985407000	1.620014000	-2.211499000
H	0.863714000	-1.918475000	-2.772514000
H	-5.448227000	4.609105000	-2.715401000
H	-6.109782000	-5.033984000	-0.378353000
H	5.232222000	2.247599000	-0.805180000
H	3.246094000	-1.516482000	-2.091743000
H	-4.647270000	-2.405173000	-0.968421000
H	6.424464000	4.936637000	0.046941000
H	-0.666415000	-5.810149000	-1.622885000
H	1.518373000	-4.797152000	-2.143916000
H	-6.677953000	-3.415873000	0.074855000
H	-6.147370000	3.711197000	-1.361759000
H	2.121412000	6.675102000	1.405401000
H	4.193570000	5.360265000	0.930546000
H	-2.807829000	4.543147000	-2.887844000
H	0.904149000	5.715728000	2.261912000
H	7.085149000	3.346547000	0.465621000
H	-1.151818000	-5.465463000	0.043569000
H	-1.264807000	-7.122713000	-0.588627000
H	1.344242000	7.332080000	2.859737000
H	-3.718662000	-5.672480000	0.581306000
H	1.503243000	-7.154937000	-2.071270000
H	-5.836697000	2.418527000	0.233298000
H	-7.309243000	0.659773000	1.169980000
H	-0.980742000	5.880874000	-2.577948000
H	0.876153000	4.251731000	-2.137047000
H	0.886735000	-8.457399000	-1.041009000
H	-6.317165000	-1.544077000	1.772259000
H	-4.581108000	-6.833353000	3.772668000
H	4.438368000	7.070783000	2.509353000
H	6.533339000	1.102393000	1.298265000
H	1.409723000	5.935830000	-1.931264000
H	-2.691226000	-6.973870000	2.150124000
H	5.625799000	-2.895582000	0.055008000
H	2.471346000	-7.727478000	-0.700330000

H	0.717454000	-6.910326000	0.881838000
H	2.407584000	5.571272000	4.250897000
H	7.310814000	-1.144592000	0.567798000
H	3.632430000	7.700547000	3.955139000
H	-3.231552000	-7.776535000	4.443454000
H	1.080414000	4.923266000	-0.514481000
H	-1.916957000	7.248563000	-0.713351000
H	-0.550443000	-1.800901000	2.111801000
H	-0.227253000	7.692207000	-1.045541000
H	4.780422000	6.350757000	4.093212000
H	-3.500782000	-6.105711000	4.976077000
H	1.000508000	-0.990897000	2.387329000
H	2.579267000	3.308553000	4.306165000
H	-2.247026000	1.227066000	1.906418000
H	1.807269000	-5.311502000	2.085786000
H	-0.746648000	4.304472000	0.835856000
H	-0.511849000	-0.133806000	2.653396000
H	-4.317358000	2.547644000	2.420929000
H	2.934116000	-0.003690000	2.866063000
H	1.162794000	1.219088000	4.177974000
H	-0.621375000	6.685881000	0.359113000
H	-3.117409000	-0.429039000	3.125160000
H	-5.443510000	-0.960289000	3.826828000
H	3.509129000	-2.062903000	2.637574000
H	-0.813439000	-7.245583000	3.712060000
H	-0.510993000	-5.829058000	2.680053000
H	5.327163000	-3.727204000	2.291019000
H	-2.562078000	-3.866450000	4.273368000
H	-3.307741000	3.913058000	2.941923000
H	-0.002371000	2.308855000	2.083702000
H	5.322176000	0.177014000	3.550167000
H	1.261831000	-2.877470000	3.547251000
H	2.354362000	1.500023000	5.466389000
H	-1.048725000	-5.610093000	4.352232000
H	4.340912000	-4.972848000	3.082390000
H	-3.282704000	2.389454000	3.854454000
H	1.897103000	-0.149927000	5.026930000
H	-4.821802000	-2.013680000	5.113878000
H	4.839145000	1.076749000	5.003813000
H	-1.189502000	-1.671461000	4.308585000
H	2.066320000	-4.384432000	4.023790000
H	-0.755727000	3.727218000	2.863202000
H	-4.533360000	-0.262394000	5.180733000
H	-0.834778000	2.149394000	3.647413000
H	4.923794000	-3.577130000	4.014242000
H	4.358940000	-0.623349000	4.809410000
H	-2.364607000	-2.131599000	5.558439000
H	2.590519000	-2.850497000	4.724758000
H	-2.019064000	-0.426632000	5.259465000

*
